# Design, Synthesis and Anticancer Activity of a New Series of *N*-aryl-*N*′-[4-(pyridin-2-ylmethoxy)benzyl]urea Derivatives

**DOI:** 10.3390/molecules26123496

**Published:** 2021-06-08

**Authors:** Shicheng Hou, Shishao Liang, Chao Zhang, Yingmei Han, Jianhui Liang, Hongyu Hu, Xingeng Zhang, Chun Hu, Xiaoping Liu, Hong Zhang

**Affiliations:** 1 Key Laboratory of Structure-Based Drug Design & Discovery, Ministry of Education, Shenyang Pharmaceutical University, Shenyang 110016, China; houshicheng2333@163.com (S.H.); liangshishao2@163.com (S.L.); zhangchaoylh@126.com (C.Z.); hanyingmei0613@163.com (Y.H.); liangjianhui1502@163.com (J.L.); huhongyu64911@163.com (H.H.); zhangxg96@163.com (X.Z.); 2 School of Life Science and Biopharmaceutics, Shenyang Pharmaceutical University, Shenyang 110016, China

**Keywords:** anticancer agent, urea derivative, synthesis, molecular hybridization, *N*-aryl-*N*’-arylmethylurea, antiproliferative activity, cell cycle analysis

## Abstract

The development of cancer treatments requires continuous exploration and improvement, in which the discovery of new drugs for the treatment of cancer is still an important pathway. In this study, based on the molecular hybridization strategy, a new structural framework with an *N*-aryl-*N*’-arylmethylurea scaffold was designed, and 16 new target compounds were synthesized and evaluated for their antiproliferative activities against four different cancer cell lines A549, MCF7, HCT116, PC3, and human liver normal cell line HL7702. The results have shown seven compounds with 1-methylpiperidin-4-yl groups having excellent activities against all four cancer cell lines, and they exhibited scarcely any activities against HL7702. Among them, compound 9b and 9d showed greatly excellent activity against the four kinds of cells, and the IC_50_ for MCF7 and PC3 cell lines were even less than 3 μM.

## 1. Introduction

Nowadays, cancer has become a major challenge in human health, and a leading cause of death [1,2]. Cancer is caused by the uncontrolled proliferation of cells, a kind of behavior unusual for cells, mostly related to some abnormal signal transduction regulation mechanisms. In the diagnosis and treatment of cancer, many great developments have been made, such as in the field of surgery, drug therapy from toxic drugs to targeted drugs, etc. [3,4]. However, traditional chemotherapy drugs often have serious side effects and adverse reactions. The emergence of small-molecule targeted drugs has eased the severe side effects of chemotherapy drugs to a certain extent, but targeted drugs are also prone to drug resistance problems with the prolonged administration time [5,6,7]. Therefore, it is a great challenge and opportunity to continuously develop new candidate drug molecules to bring new drugs to cancer treatment.

**Figure 1:**
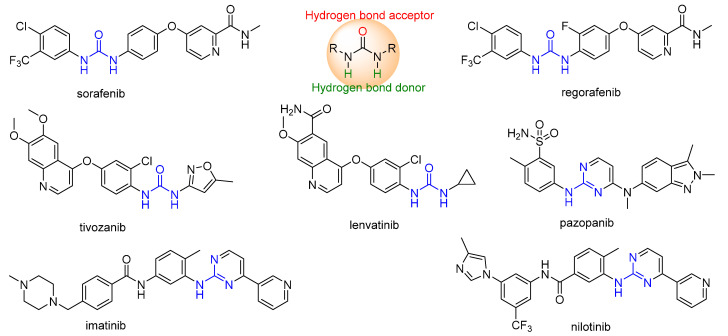
The application of urea and its isosteres in anticancer drugs.

Compared with earlier targeted drugs, multi-target inhibitors can act on a variety of different targets and inhibit different signal pathways at the same time [6,8,9]. The multi-target inhibitors have a broader anti-tumor spectra and better prospects in clinical practice [7]. Urea and urea isosteres are structures that possesses both a hydrogen bond acceptor and a hydrogen bond donor, which makes it easy to form better interactions with drug target proteins [10]. These kinds of structures are an excellent pharmacodynamic structure in drug molecules [11]. In many small-molecule targeted kinase inhibitors, urea and urea isosteres, including aminopyrimidine, have been used in drug structures, some of which showed really favorable anti-cancer activity (Figure 1) [12,13].

**Figure 2:**
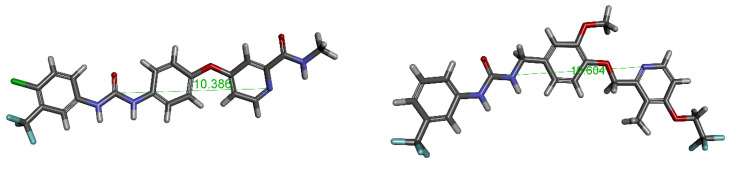
Spatial distance of the two nitrogen atoms in pyridine ring and urea moiety of sorafenib (**left**) and a target compound (**right**).

Among the currently clinically used multi-target kinase inhibitors, sorafenib and its derivative regorafenib with a diaryl urea structure were the prime representatives, because they had excellent inhibitory effects on a variety of solid tumors [14,15]. In sorafenib, the rigidity of the diaryl urea structure causes the molecular rotation to not happen freely, thus the poor solubility of sorafenib results in low bioavailability [16,17]. This study is based on the structure of sorafenib and the retention of the urea scaffold, in which, in order to enhance the molecular flexibility, a carbon atom was inserted into the diaryl urea structure, and the basic urea scaffold was changed to an *N*-aryl-*N*’-benzylurea scaffold. Moreover, the diaryl ether fragment with a pyridyl group was also modified to a 4-(pyridylmethoxy)phenyl fragment. The nitrogen atom in the pyridine ring is believed to continue to play a key role, for example, as a hydrogen bond acceptor with some proteins. Meanwhile, in order to keep its position relative to the core fragment urea unchanged, the position of the nitrogen atom in the pyridine ring linked to the core urea fragment was replaced from the 4-position to the 2-position. Validity has been verified by a simulation using the Discovery Studio 3.0 software [18], and the distances between the nitrogen atom in the pyridine ring and the urea moiety in sorafenib and a target compound are 10.386Å and 10.604Å, respectively (Figure 2).

**Figure 3:**
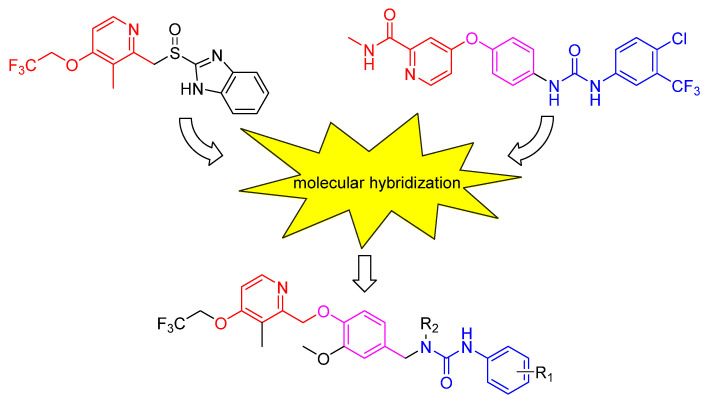
Design of target compounds with molecular hybridization strategy.

Furthermore, in previous reports, some proton pump inhibitors showed anticancer activity; lansoprazole was one of the drugs that performed well [19,20]. Based on the molecular hybridization and structural optimization strategies, the 3-methyl-4-(2,2,2-trifluoroethoxy)pyridin-2-ylmethyl moiety in lansoprazole and the *N*-aryl-*N*’-benzylurea scaffold were retained into the target compounds (Figure 3) [21]. Considering the extra space in some intracellular protein serine/threonine kinases such as BRAF kinase binding with sorafenib, a new series of *N*-aryl-*N*’-benzylurea derivatives modified with a 1-methylpiperidin-4-yl group on the 3-position of the urea scaffold were designed, which are expected to block intracellular signal transduction and enhance their antiproliferative activity [22,23].

## 2. Results and discussion

### 2.1. Chemistry

**Scheme 1:**
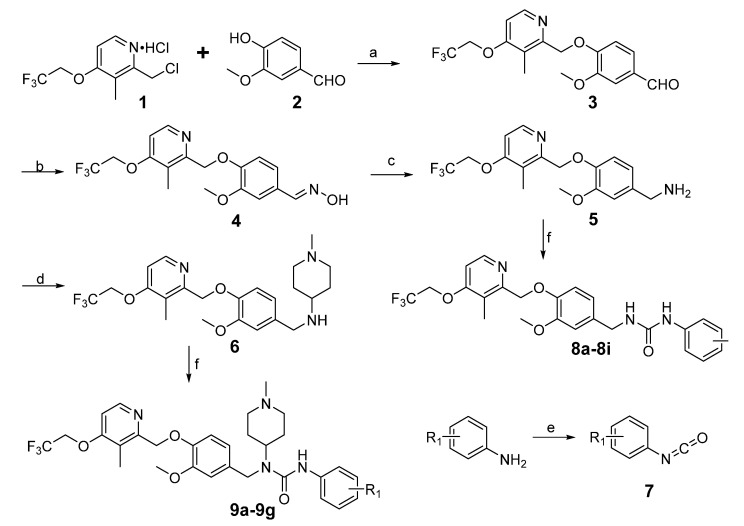
Synthetic route of the target compounds. Reagents and conditions: a. K_2_CO_3_, DMF, 80 °C, yield 97%; b. NH_2_OH·HCl, NaHCO_3_, EtOH, H_2_O, yield 98%; c. Ni-Al, NaOH, EtOH, H_2_O, yield 95%; d. 1-methylpiperidin-4-one, NaBH_3_CN, AcOH, MeOH yield 77%; e. amine, triphosgene, TEA, DCM; f. isocyanate 7, DCM.

As shown in Scheme 1, the target molecules were synthesized starting with 2-(chloromethyl)-3-methyl-4-(2,2,2-trifluoromethoxy)pyridine hydrochloride (**1**) and vanillin (**2**) via a Williamson reaction and obtained the ether compound **3,** which reacted with hydroxylamine to convert the oxime **4**. The oxime **4** was reduced by Ni-Al to obtain benzylamine **5**. The reaction between benzylamine **5** and 1-methylpiperidin-4-one through reductive amination yielded the key intermediate **6**. Finally, the different intermediate isocyanate **7**, prepared by a reaction between substituting aniline or benzylamine, triphosgene, and TEA, were mixed with compound **5** or compound **6** to yield the targeted compounds **8a**–**8i** and **9a**–**9g**.

### 2.2. Biological Activity Evaluation

The target compounds were evaluated for their antiproliferative activity against different human cancer cell lines, including A549 (non-small cell lung cancer cell line), MCF-7 (breast cancer cell line), HCT116 (colon cancer cell line), PC-3 (prostate cancer cell line) and HL7702 (human liver normal cell line) by using the MTT assay with sorafenib as the control drug. The evaluated results as IC_50_ values are shown in Table 1.

As shown in Table 1, all the target compounds exhibited weak cytotoxic activities against HL7702, and most of the target compounds exhibit excellent antiproliferative activity against the A549 cell line and HCT116 cell line. The IC_50_ values of target compounds **8c**, **9b,** and **9d** against the A549 cells line were less than 5 μM, and the IC_50_ values of compounds **9b**, **9d,** and **9g** against the HCT116 cell line were less than 3 μM. The antiproliferative activities against the MCF7 cell line and the PC3 cell line of target compounds **9a**–**9g** with the 1-methylpiperidin-4-yl group were significantly higher than that of compounds **8a**–**8i** without the 1-methylpiperidin-4-yl group. The IC_50_ values of compounds **9a**, **9b**, **9d**, and **9e** against the MCF7 cell line and the PC3 cell line were less than 3 μM and 5 μM, respectively. Among them, target compounds **9b** and **9d** have shown a more potent antiproliferative activity against the four cancer cell lines with excellent IC_50_ values (under 5 μM) compared to the control drug sorafenib.

The analyses of the structure-activity relationships of the target compounds with the 1-methylpiperidin-4-yl group were summarized as follows: (1) The introduction of fluorine atoms on the R_1_ substituent of the benzene ring was mostly beneficial to the antiproliferative activity. For example, Compounds **9a**, **9b**, **9d**, and **9e** with the fluorine atoms in substituent on the phenyl show a better antiproliferative activity against the MCF7 cell line and the PC3 cell line than the control drug sorafenib, especially in the MCF7 cell line. The inhibitory activities against several cell lines of compound **9f** with the nitro group were relatively weaker than that of several compounds with fluorine atoms on the R1 substituent of the benzene ring. (2) The introduction of electron-withdrawing group substituent R_1_ on the benzene ring results in an increase in antiproliferative activity. The antiproliferative activities against all four cancer cell lines of the target compounds **9b** and **9d** with a trifluoromethyl group were significantly higher than compound **9a** with a methoxy group and **9c** with a trifluoromethoxy group.

The antiproliferative activities of the target compounds without the 1-methylpiperidin-4-yl group have shown similar structure-activity relationships. For example, the antiproliferative activity against the HCT116 cell line of compounds **8a**, **8b**, **8c**, and **8d** with fluorine atoms in substituent on the phenyl were also better than that of the other compounds without fluorine atoms, and the IC_50_ value of compound **8c** was less than 5 μM against the A549 cell line.

### 2.3. Cell Cycle Analysis

**Figure 4:**
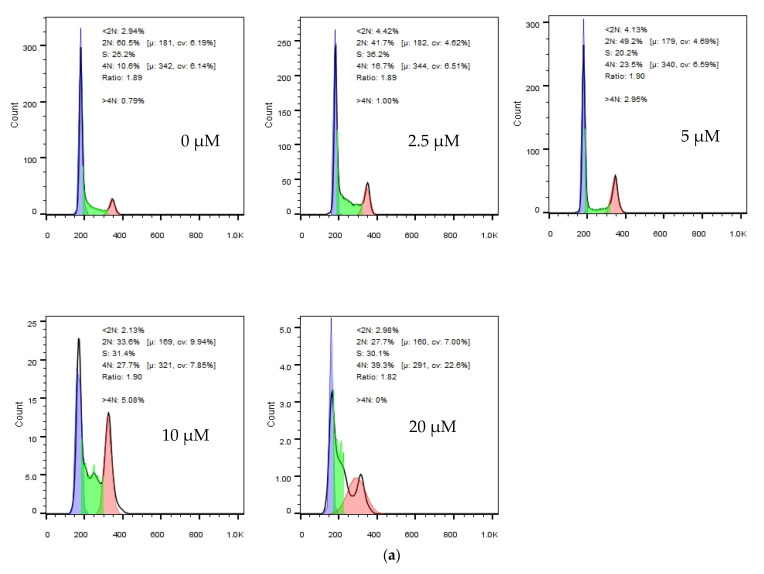

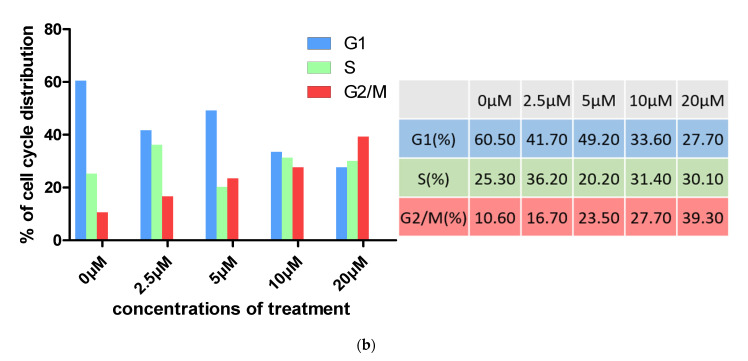
Effects of compound **9b** on MCF7 cell cycle progress for 24 h. (**a**) Treatment of MCF7 cells with compound **9b** at different concentrations (0 μM, 2.5 μM, 5 μM, 10 μM, 20 μM) for 24 h. (**b**) Quantitative analysis of cell cycle.

The effect of compound **9b** on the cell cycle was assayed. After treatment of MCF-7 cells with compound **9b** for 72 h at different concentrations (2.5, 5, 10, 20 μM), the percentages of cells in the G_2_/M phase were 16.7%, 23.5%, 27.7%, and 39.3%, respectively (Figure 4), indicating that compound **9b** could cause an obvious G2/M arrest in a concentration-dependent manner with a concomitant decrease in terms of the number of cells in other phases of the cell cycle.

## 3. Materials and Methods

### 3.1. Chemistry

#### 3.1.1. General Information

All reagents were obtained from commercial suppliers and used without further purification. The progress of the reactions was monitored by thin-layer chromatography (TLC) on silica gel plates and the spots visualized under ultraviolet (UV) light (254 nm). The column chromatography was performed using 200–300 mesh silica gel (Qingdao Haiyang, Qingdao, China). Mass spectra were measured with an electrospray (ESI-MS) on an Agilent 1100 Series LC/MSD Trap (Agilent Corporation, Santa Clara, CA, USA). ^1^H-NMR and ^13^C-NMR spectra were recorded on Bruker NMR spectrophotometers (Karlsruhe, Germany) using DMSO-*d*_6_ as the solvent. The IR spectra were measured using a Bruker Fourier number infrared spectrometer (Agilent Corporation, Santa Clara, CA, USA). ^1^H-NMR, ^13^C-NMR, ESI-MS and HRMS spectra of the target compounds are available in the Supplementary Material (Figures S1–S80).

#### 3.1.2. Synthesis of 3-methoxy-4-[(3-methyl-4-(2,2,2-trifluoroethoxy)pyridin-2-yl)methoxy] benzaldehyde (**3**)

A mixture of 2-(chloromethyl)-3-methyl-4-(2,2,2-trifluoroethoxy)pyridine hydrochloride **1** (27.6 g, 100 mmol), vanillin **2** (15.22 g, 100 mmol), K_2_CO_3_ (69 g, 0.5 mol) and DMF(100 mL) was stirred at 80 °C overnight. The reaction was monitored by TLC (PE:EA = 3:1). The mixture was poured into 750 mL of water and stirred for 30 min until the K_2_CO_3_ was completely dissolved. The solids that precipitated out were filtered, washed with 2 mol/L NaOH aqueous solution and water, then dried to obtain a white solid 34.54 g. The yield was 97%, ESI-MS: 356.3([M + H]^+^).

#### 3.1.3. Synthesis of (E)-3-methoxy-4-((3-methyl-4-(2,2,2-trifluoroethoxy)pyridin-2-yl) methoxy)benzaldehyde oxime (**4**)

A mixture of hydroxylamine hydrochloride (3.06 g, 44 mmol) and NaHCO_3_ (3.69 g, 44 mmol) in water (50 mL) was stirred at room temperature until no gas was released. A solution of 3-methoxy-4-((3-methyl-4-(2,2,2-trifluoroethoxy)pyridin-2-yl)methoxy)benzaldehyde **3** (14.21 g,40 mmol) in EtOH (50 mL) was added into the mixture and continued string for 3 h. The progress of the reaction can be confirmed by TLC. EtOH was removed under reduced pressure, and the white solid was precipitated, filtered off with suction, and washed with water. After drying, the white solid obtained was 14.6 g, with a yield of 98%, ESI-MS: 371.3([M + H]^+^).

#### 3.1.4. Synthesis of (3-methoxy-4-((3-methyl-4-(2,2,2-trifluoroethoxy)pyridin-2-yl)methoxy)phenyl)methanamine (**5**)

An aqueous solution of NaOH (5 mol/L, 60 mL) was added into the solution of 3-methoxy-4-((3-methyl-4-(2,2,2-trifluoroethoxy)pyridin-2-yl)methoxy)benzaldehyde oxime **4** (11.10 g,30 mmol) in EtOH (50 mL) under an ice bath. Nickel-aluminum alloy (10 g) was slowly added into the mixture in several times, during which a lot of gas was generated. Then slowly returned to room temperature and stirred overnight. The progress of the reaction was monitored by TLC. After removing the solid by suction filtration, EtOH was distilled off under reduced pressure. The residual solution was extracted by EA, and the organic phase was washed by water and brine, then dried by Na_2_SO_4_. After the solvent was removed under reduced pressure, the white solid obtained was 10.14 g, with a yield of 95%, ESI-MS: 357.2([M + H]^+^).

#### 3.1.5. Synthesis of N-(3-methoxy-4-((3-methyl-4-(2,2,2-trifluoroethoxy)pyridin-2-yl) methoxy)benzyl)-1-methylpiperidin-4-amine (**6**)

A mixture of 1-methylpiperidin-4-one (2.26 g, 20 mmol), (3-methoxy-4-((3-methyl-4-(2,2,2-trifluoroethoxy)pyridin-2-yl)methoxy)phenyl)methanamine(7.12 g, 20 mmol) **5**, AcOH and MeOH was stirred for 1h at room temperature. NaBH_3_CN was added in 3 times, during which a lot of gas was generated and stirring was continued for 3 h. The progress of the reaction was monitored by TLC. After MeOH was distilled off under reduced pressure, a paste mixture was obtained. An aqueous solution of NaOH (2 mol/L) was added to the mixture and stirred until the paste dissolved. The solution was extracted by EA, and the organic phase was washed by water and brine, then dried by Na_2_SO_4_. After the solvent was removed under reduced pressure, a yellowish oil of 7.02 g was obtained, with a yield of 77%, 454.2([M + H]^+^).

#### 3.1.6. General Procedure for the Synthesis of the Target Urea Derivatives

Triphosgene (0.20 g, 0.67 mmol) was dissolved in 10 mL DCM, a solution of substituted aniline or benzylamine (2 mmol) in 10 mL DCM was slowly dropped in during stirring. There were solids that gradually precipitated out. Then a solution of TEA (0.4 g, 4 mmol) in DCM (10 mL) was slowly dropped into the mixture, the solids gradually dissolved, and the solution of substituted isocyanate 7 was obtained. The solution of **5** or **6** (2 mmol) in 10 mL DCM was added. After the reaction was completed, the mixture was washed by water and brine and dried by Na_2_SO_4_. After DCM was distilled off under reduced pressure, the mixture was purified by silica gel chromatography (DCM:EA = 5:1, *v*/*v*) to afford **8a**–**8i** and silica gel chromatography (DCM:MeOH = 20:1, *v*/*v*) to afford **9a**–**9g**.

 *1-(3-methoxy-4-((3-methyl-4-(2,2,2-trifluoroethoxy)pyridin-2-yl)methoxy)benzyl)-3-(4-(trifluoromethoxy)phenyl)urea* (**8a**), white powder 0.99 g, yield 89%; m.p.: 158.8–159.3 °C; MS: 560.4([M + H]^+^); ^1^H NMR (400 MHz, DMSO-*d*_6_) δ 8.73 (s, 1H), 8.34 (d, *J* = 5.7 Hz, 1H), 7.54–7.47 (m, 2H), 7.23 (d, *J* = 8.6 Hz, 2H), 7.14 (d, *J* = 5.7 Hz, 1H), 7.05 (d, *J* = 8.2 Hz, 1H), 6.94 (d, *J* = 2.0 Hz, 1H), 6.81 (dd, *J* = 8.2, 2.0 Hz, 1H), 6.59 (t, *J* = 5.9 Hz, 1H), 5.14 (s, 2H), 4.92 (q, *J* = 8.8 Hz, 2H), 4.23 (d, *J* = 5.7 Hz, 2H), 3.74 (s, 3H), 2.22 (s, 3H).; ^13^C NMR (101 MHz, DMSO-*d*_6_) δ 161.82, 155.94, 155.51, 149.63, 148.02, 147.18, 142.55, 140.26, 133.67, 124.25 (q, *J* = 277.9 Hz), 122.08, 121.99, 120.69 (q, *J* = 254.9 Hz), 119.69, 119.19, 114.27, 112.07, 108.02, 71.56, 65.11 (q, *J* = 34.7 Hz), 55.95, 43.10, 10.30; IR: 3399, 3053, 3008, 2971, 2829, 1706, 1556, 1507, 1454, 1416, 1256, 1220, 1194, 1154, 1015, 911, 847, 796, 672, 645, 544; HRMS: 558.146914([M − H]^−^) for C_25_H_22_F_6_N_3_O_5_, 560.161467([M + H]^+^) for C_25_H_24_F_6_N_3_O_5_.

 *1-(3-chloro-4-fluorophenyl)-3-(3-methoxy-4-((3-methyl-4-(2,2,2-trifluoroethoxy)pyridin-2-yl)methoxy)benzyl)urea* (**8b**), white powder 0.86 g, yield 82%; m.p.: 167.8–169.1 °C; MS:528.3([M + H]^+^); ^1^H NMR (400 MHz, DMSO-*d*_6_) δ 8.72 (s, 1H), 8.34 (d, *J* = 5.7 Hz, 1H), 7.77 (dd, *J* = 6.8, 2.4 Hz, 1H), 7.32–7.18 (m, 2H), 7.14 (d, *J* = 5.7 Hz, 1H), 7.04 (d, *J* = 8.2 Hz, 1H), 6.93 (d, *J* = 2.0 Hz, 1H), 6.80 (dd, *J* = 8.2, 1.9 Hz, 1H), 6.63 (t, *J* = 5.8 Hz, 1H), 5.13 (s, 2H), 4.91 (q, *J* = 8.7 Hz, 2H), 4.22 (d, *J* = 5.7 Hz, 2H), 3.74 (s, 3H), 2.22 (s, 3H).; ^13^C NMR (101 MHz, DMSO-*d*_6_) δ 161.81, 155.94, 155.44, 152.33 (d, *J* = 240.0 Hz), 149.59, 148.04, 147.15, 138.26 (d, *J* = 2.8 Hz), 124.27 (q, *J* = 277.4 Hz), 122.05, 119.69, 119.44 (d, *J* = 18.9 Hz), 119.31, 118.23 (d, *J* = 6.5 Hz), 117.13 (d, *J* = 21.3 Hz), 114.26, 112.08, 108.06, 71.55, 65.08 (q, *J* = 34.2 Hz), 56.00, 43.09, 10.35; IR: 3313, 2944, 2883, 1641, 1564, 1500, 1477, 1420, 1390, 1308, 1258, 1209, 1164, 1131, 1008, 970, 911, 862, 800, 757, 647, 576, 445; HRMS: 526.116220([M − H]^−^) for C_24_H_21_ClF_4_N_3_O_4_, 528.130773([M + H]^+^) for C_24_H_23_ClF_4_N_3_O_4_.

 *1-(3-methoxy-4-((3-methyl-4-(2,2,2-trifluoroethoxy)pyridin-2-yl)methoxy)benzyl)-3-(4-(trifluoromethyl)phenyl)urea* (**8c**), white powder 0.78 g, yield 72%; m.p: 169.9–171.0 °C; MS:544.5([M + H]^+^), 566.1([M + Na]^+^); ^1^H NMR (400 MHz, DMSO-*d*_6_) δ 8.95 (s, 1H), 8.34 (d, *J* = 5.7 Hz, 1H), 7.59 (q, *J* = 8.8 Hz, 4H), 7.14 (d, *J* = 5.7 Hz, 1H), 7.05 (d, *J* = 8.2 Hz, 1H), 6.95 (d, *J* = 2.0 Hz, 1H), 6.82 (dd, *J* = 8.2, 1.9 Hz, 1H), 6.69 (t, *J* = 5.8 Hz, 1H), 5.14 (s, 2H), 4.91 (q, *J* = 8.7 Hz, 2H), 4.24 (d, *J* = 5.7 Hz, 2H), 3.74 (s, 3H), 2.22 (s, 3H). ^13^C NMR (101 MHz, DMSO-*d*_6_) δ 161.81, 155.93, 155.25, 149.61, 148.03, 147.19, 144.64, 133.47, 126.37, 125.09 (q, *J* = 270.6 Hz), 124.26 (q, *J* = 277.5 Hz), 122.06, 119.73, 117.72, 114.26, 112.10, 108.05, 71.55, 65.09 (q, *J* = 35.0 Hz), 55.98, 43.10, 10.31; IR: 3414, 3376, 2940, 2886, 1703, 1686, 1581, 1534, 1512, 1477, 1408, 1321, 1256, 1220, 1180, 1155, 1135, 1102, 1063, 1008, 979, 862, 842, 812, 595, 554;HRMS: 542.151999 ([M − H]^−^) for C_25_H_22_F_6_N_3_O_4_, 544.166552([M + H]^+^)^+^ for C_25_H_24_F_6_N_3_O_4_.

 *1-(3-methoxy-4-((3-methyl-4-(2,2,2-trifluoroethoxy)pyridin-2-yl)methoxy)benzyl)-3-(3-(trifluoromethyl)phenyl)urea* (**8d**), white powder 0.83 g, yield 76%; m.p.: 153.2–154.2 °C; MS:544.2([M + H]^+^), 542.0([M − H]^−^); ^1^H NMR (400 MHz, DMSO-*d*_6_) δ 8.90 (s, 1H), 8.34 (d, *J* = 5.7 Hz, 1H), 7.98 (s, 1H), 7.52 (d, *J* = 8.3 Hz, 1H), 7.45 (t, *J* = 7.9 Hz, 1H), 7.23 (d, *J* = 7.6 Hz, 1H), 7.14 (d, *J* = 5.7 Hz, 1H), 7.04 (d, *J* = 8.2 Hz, 1H), 6.94 (d, *J* = 1.9 Hz, 1H), 6.81 (dd, *J* = 8.2, 1.9 Hz, 1H), 6.68 (t, *J* = 5.9 Hz, 1H), 5.13 (s, 2H), 4.91 (q, *J* = 8.7 Hz, 2H), 4.23 (d, *J* = 5.8 Hz, 2H), 3.74 (s, 3H), 2.22 (s, 3H). ^13^C NMR (101 MHz, DMSO-*d*_6_) δ 161.82, 155.94, 155.46, 149.62, 148.02, 147.18, 141.80, 133.59, 130.11, 124.73 (q, *J* = 272.3 Hz), 124.25 (q, *J* = 277.4 Hz), 122.07, 121.62, 119.69, 117.68, 114.28, 114.06, 112.08, 108.02 (d, *J* = 4.4 Hz), 71.56, 65.10 (q, *J* = 34.2 Hz), 55.97 (d, *J* = 3.2 Hz), 43.08, 10.33; IR: 3412, 3374, 2940, 2876, 1702, 1582, 1551, 1514, 1477, 1442, 1380, 1341, 1312, 1255, 1221, 1182, 1158, 1111, 1067, 1028, 1007, 979, 891, 864, 813, 796, 702, 664, 597, 552; HRMS: 542.151999([M − H]^−^) for C_25_H_22_F_6_N_3_O_4_^+^, 544.161552 ([M + H]^+^) for C_25_H_24_F_6_N_3_O_4_.

 *1-(3-methoxy-4-((3-methyl-4-(2,2,2-trifluoroethoxy)pyridin-2-yl)methoxy)benzyl)-3-(4-methoxyphenyl)urea* (**8e**), white powder 0.59 g, yield 58%; m.p.: 179.5–180.3 °C; MS:566.2([M + H]^+^), 504.0([M − H]^−^); ^1^H NMR (400 MHz, DMSO-*d*_6_) δ 8.28 (s, 1H), 7.29 (d, *J* = 8.9 Hz, 2H), 7.14 (d, *J* = 5.7 Hz, 1H), 6.93 (d, *J* = 1.9 Hz, 1H), 6.81 (dd, *J* = 9.0, 7.1 Hz, 3H), 6.40 (t, *J* = 5.9 Hz, 1H), 5.13 (s, 2H), 4.92 (q, *J* = 8.8 Hz, 2H), 4.20 (d, *J* = 5.8 Hz, 2H), 3.70 (s, 3H), 2.22 (s, 3H). ^13^C NMR (101 MHz, DMSO-*d*_6_) δ 161.80, 155.96, 155.86, 154.40, 149.57, 148.05, 147.08, 134.08, 133.97, 124.28 (d, *J* = 277.9 Hz), 122.05, 119.91, 119.64, 114.33, 114.27, 112.06, 108.07, 71.58, 65.08 (q, *J* = 34.5 Hz), 55.99, 55.59, 43.09, 10.36; IR: 3312, 2940, 2839, 1631, 1571, 1508, 1467, 1417, 1376, 1363, 1308, 1271, 1241, 1160, 1136, 1030, 973, 862, 827, 669, 578, 524, 423; HRMS: 504.175179 ([M − H]^−^) for C_25_H_25_F_3_N_3_O_5_, 506.189732 ([M + H]^+^) for C_25_H_27_F_3_N_3_O_5_.

 *1-(3-methoxy-4-((3-methyl-4-(2,2,2-trifluoroethoxy)pyridin-2-yl)methoxy)benzyl)-3-(3-nitrophenyl)urea* (**8f**), yellow powder 0.31 g, yield 30%; m.p.: 168.9–170.5 °C; MS: 521.2([M + H]^+^), 519.0([M − H]^−^); ^1^H NMR (400 MHz, DMSO-*d*_6_) δ 9.08 (s, 1H), 8.52 (t, *J* = 2.2 Hz, 1H), 8.33 (d, *J* = 5.8 Hz, 1H), 7.75 (d, *J* = 8.3 Hz, 1H), 7.66 (d, *J* = 8.4 Hz, 1H), 7.50 (dd, *J* = 9.1, 7.3 Hz, 1H), 7.13 (d, *J* = 5.9 Hz, 1H), 7.04 (d, *J* = 8.2 Hz, 1H), 6.96–6.92 (m, 1H), 6.85–6.71 (m, 2H), 5.13 (s, 2H), 4.91 (q, *J* = 8.8 Hz, 2H), 4.24 (d, *J* = 5.9 Hz, 2H), 3.74 (s, 3H), 2.21 (s, 3H). ^13^C NMR (101 MHz, DMSO-*d*_6_) δ 161.83, 155.93, 155.35, 149.63, 148.59, 148.02, 147.20, 142.28, 133.51, 130.31, 124.26 (q, *J* = 277.8 Hz), 124.16, 122.07, 119.74, 115.95, 114.32, 112.14, 112.06, 108.05, 71.56, 65.11 (q, *J* = 34.4 Hz), 56.02, 43.11, 10.34; IR: 3410, 3010, 2943, 2882, 2832, 1701, 1584, 1527, 1503, 1480, 1383, 1347, 1318, 1264, 1207, 1161, 1138, 1122, 1033, 1000, 980, 868, 814, 794, 735, 671, 612, 585, 557, 445; HRMS: 519.149693 ([M − H]^−^) for C_24_H_22_F_3_N_4_O_6_, 521.164246([M + H]^+^) for C_24_H_24_F_3_N_4_O_6_.

 *1-(3-methoxy-4-((3-methyl-4-(2,2,2-trifluoroethoxy)pyridin-2-yl)methoxy)benzyl)-3-(4-methoxybenzyl)urea* (**8g**), white powder 0.39 g, yield 38%; m.p.: 153.0–154.2 °C; MS: 520.2([M + H]^+^), 517.9([M − H]^−^); ^1^H NMR (400 MHz, DMSO-*d*_6_) δ 9.08 (s, 1H), 8.52 (t, *J* = 2.2 Hz, 1H), 8.33 (d, *J* = 5.8 Hz, 1H), 7.75 (d, *J* = 8.3 Hz, 1H), 7.66 (d, *J* = 8.4 Hz, 1H), 7.50 (dd, *J* = 9.1, 7.3 Hz, 1H), 7.13 (d, *J*= 5.9 Hz, 1H), 7.04 (d, *J* = 8.2 Hz, 1H), 6.96–6.92 (m, 1H), 6.85–6.71 (m, 2H), 5.13 (s, 2H), 4.91 (q, *J* = 8.8 Hz, 2H), 4.24 (d, *J* = 5.9 Hz, 2H), 3.74 (s, 3H), 2.21 (s, 3H); ^13^C NMR (101 MHz, DMSO-*d*_6_) δ 161.82, 158.54, 158.46, 155.99, 149.57, 148.04, 146.99, 134.47, 133.30, 124.28 (q, *J* = 277.7 Hz), 122.06, 119.46, 114.26, 114.07, 111.86, 108.08, 71.61, 65.10 (q, *J* = 34.9 Hz), 55.92, 55.51, 43.25, 42.90, 10.36; IR: 3349, 3301, 2949, 2925, 2884, 2832, 1605, 1579, 1561, 1515, 1468, 1424, 1363, 1363, 1363, 1275, 1251, 1169, 1156, 1133, 1103, 1038, 975, 863, 816, 728, 637, 561; HRMS: 518.190829 ([M − H]^−^) for C_26_H_27_F_3_N_3_O_5_, 520.205382 ([M + H]^+^) for C_26_H_29_F_3_N_3_O_5_.

 *1-(4-ethoxybenzyl)-3-(3-methoxy-4-((3-methyl-4-(2,2,2-trifluoroethoxy)pyridin-2-yl)methoxy)benzyl)urea* (**8h**), white powder 0.57 g, yield 53%; m.p.: 148.9–149.9 °C; MS: 534.3 ([M + H]^+^); ^1^H NMR (400 MHz, DMSO-*d*_6_) δ 8.34 (d, *J* = 5.7 Hz, 1H), 7.20–7.11 (m, 3H), 7.02 (d, *J* = 8.2 Hz, 1H), 6.90–6.81 (m, 3H), 6.75 (dd, *J* = 8.3, 1.9 Hz, 1H), 6.30 (td, *J* = 6.1, 2.3 Hz, 2H), 5.13 (s, 2H), 4.92 (q, *J* = 8.8 Hz, 2H), 4.15 (d, *J* = 5.8 Hz, 4H), 3.99 (q, *J* = 7.0 Hz, 2H), 3.71 (s, 3H), 2.22 (s, 3H), 1.31 (t, *J* = 7.0 Hz, 3H); ^13^C NMR (101 MHz, DMSO-*d*_6_) δ 161.82, 158.48, 157.77, 155.96, 149.54, 148.02, 146.96, 134.46, 133.16, 128.76, 124.28 (q, *J*= 277.7, 277.3 Hz), 122.06, 119.44, 114.56, 114.21, 111.81, 108.08, 71.57, 65.08 (q, *J* = 34.5 Hz), 63.39, 55.89, 43.24, 42.89, 15.11, 10.36; IR: 3331, 2978, 2927, 2883, 1609, 1579, 1556, 1516, 1471, 1423, 1390, 1275, 1249, 1148, 1025, 974, 861, 815, 753, 728, 639, 574, 548; HRMS: 532.206479 ([M − H]^−^) for C_27_H_29_F_3_N_3_O_5_, 534.221032([M + H]^+^) for C_27_H_31_F_3_N_3_O_5_.

 *1-(4-(dimethylamino)benzyl)-3-(3-methoxy-4-((3-methyl-4-(2,2,2-trifluoroethoxy)pyridin-2-yl)methoxy)benzyl)urea* (**8i**), white powder 0.73 g, yield 69%; m.p.: 159.4–161.4 °C; MS:533.9([M + H]^+^), 555.4([M + Na]^+^); ^1^H NMR (400 MHz, DMSO-*d*_6_) δ 8.34 (d, *J* = 5.6 Hz, 1H), 7.14 (d, *J* = 5.7 Hz, 1H), 7.11–7.06 (m, 2H), 7.02 (d, *J* = 8.2 Hz, 1H), 6.87 (d, *J* = 2.0 Hz, 1H), 6.74 (dd, *J* = 8.2, 1.9 Hz, 1H), 6.69–6.65 (m, 2H), 6.22 (dt, *J* = 21.9, 5.9 Hz, 2H), 5.12 (s, 2H), 4.92 (q, *J* = 8.7 Hz, 2H), 4.15 (d, *J* = 5.9 Hz, 2H), 4.10 (d, *J* = 5.8 Hz, 2H), 3.71 (s, 3H), 2.85 (s, 6H), 2.22 (s, 3H). ^13^C NMR (101 MHz, DMSO-*d*_6_) δ 161.81, 158.46, 155.98, 149.97, 149.55, 148.04, 146.96, 134.50, 128.77, 128.49, 124.28 (q, *J* = 277.7 Hz), 122.05, 119.44, 114.22, 112.85, 111.82, 108.08, 71.59, 65.08 (q, *J* = 34.3 Hz), 55.92, 43.23, 43.06, 10.36; IR: 3336, 2940, 2918, 2879, 1613, 1570, 1517, 1468, 1421, 1308, 1256, 1233, 1175, 1137, 1039, 1011, 969, 922, 854, 810, 736, 650, 564; HRMS: 531.222464 ([M − H]^−^) for C_27_H_30_F_3_N_4_O_4_, 522.237017 ([M + H]^+^) for C_27_H_32_F_3_N_4_O_4_.

 *1-(3-methoxy-4-((3-methyl-4-(2,2,2-trifluoroethoxy)pyridin-2-yl)methoxy)benzyl)-1-(1-methylpiperidin-4-yl)-3-(4-(trifluoromethoxy)phenyl)urea* (**9a**), white powder 0.79 g, yield 60%; m.p.: 127.3–129.4 °C; MS:657.2([M + H]^+^); 1H NMR (400 MHz, DMSO-*d*_6_) δ 8.52 (s, 1H), 8.33 (d, *J* = 5.6 Hz, 1H), 7.58–7.51 (m, 2H), 7.23 (d, *J* = 8.6 Hz, 2H), 7.14 (d, *J* = 5.7 Hz, 1H), 7.03 (d, *J* = 8.3 Hz, 1H), 6.90 (d, *J* = 2.0 Hz, 1H), 6.75 (dd, *J* = 8.2, 1.9 Hz, 1H), 5.11 (s, 2H), 4.91 (q, *J* = 8.7 Hz, 2H), 4.52 (s, 2H), 4.10 (tt, *J* = 12.0, 4.0 Hz, 1H), 3.70 (s, 3H), 2.83 (d, *J* = 11.6 Hz, 2H), 2.20 (d, *J* = 5.4 Hz, 6H), 2.06 (d, *J* = 10.0 Hz, 2H), 1.70 (tt, *J* = 12.4, 6.7 Hz, 2H), 1.55 (d, *J* = 14.4 Hz, 2H). ^13^C NMR (101 MHz, DMSO-*d*_6_) δ 161.81, 155.73, 149.48, 148.05, 146.93, 143.15, 140.30, 133.64, 124.26(q, *J* =276.1Hz), 121.50, 118.77, 120.66 (q, *J* = 253.5 Hz), 111.28, 108.07, 71.55, 65.11 (q, *J* = 34.8, 34.0 Hz), 55.95, 55.08, 53.09, 45.99, 45.70, 45.16, 39.69, 29.90, 10.36; IR: 3397, 2950, 2848, 2799, 1648, 1584, 1513, 1470, 1416, 1377, 1293, 1256, 1227, 1204, 1159, 1132, 1031, 982, 921, 847, 825, 800, 754, 536; HRMS: 655.236063 ([M − H]^−^) for C_31_H_33_F_6_N_4_O_5_, 657.250616 ([M + H]^+^) for C_31_H_35_F_6_N_4_O_5_.

 *1-(3-methoxy-4-((3-methyl-4-(2,2,2-trifluoroethoxy)pyridin-2-yl)methoxy)benzyl)-1-(1-methylpiperidin-4-yl)-3-(4-(trifluoromethyl)phenyl)urea* (**9b**), white powder 0.73 g, yield 57%; m.p.: 138.4–140.5 °C; MS: 641.1([M + H]^+^), 321.5 ([M + 2H]^2+^), 639.5([M − H]^−^); ^1^H NMR (400 MHz, DMSO-*d*_6_) δ 8.73 (s, 1H), 8.33 (d, *J* = 5.6 Hz, 1H), 7.67 (d, *J* = 8.6 Hz, 2H), 7.58 (d, *J* = 8.7 Hz, 2H), 7.14 (d, *J* = 5.7 Hz, 1H), 7.03 (d, *J* = 8.3 Hz, 1H), 6.89 (d, *J* = 2.0 Hz, 1H), 6.75 (dd, *J* = 8.3, 2.0 Hz, 1H), 5.10 (s, 2H), 4.91 (q, *J* = 8.7 Hz, 2H), 4.54 (s, 2H), 4.16–4.07 (m, 1H), 3.70 (s, 3H), 2.84 (d, *J* = 10.9 Hz, 2H), 2.19 (s, 6H), 2.06 (t, *J* = 10.0 Hz, 2H), 1.72 (q, *J* = 12.6, 11.8 Hz, 2H), 1.57 (d, *J* = 11.4 Hz, 2H). ^13^C NMR (101 MHz, DMSO-*d*_6_) δ 161.81, 155.92, 155.50, 149.49, 148.05, 146.97, 144.86, 133.47, 125.91 (q, *J* = 3.9 Hz), 125.08 (q, *J* = 270.9 Hz), 124.26 (q, *J* = 277.6, 277.1 Hz), 122.16 (q, *J* = 31.8 Hz), 122.08, 118.76, 114.21, 111.26, 108.06, 71.54, 65.11 (q, *J* = 34.4 Hz), 55.94, 55.06, 53.24, 45.70, 45.25, 29.88, 10.34; IR: 3387, 2943, 2846, 2802, 1650, 1584, 1513, 1468, 1416, 1378, 1313, 1250, 1224, 1162, 1131, 1063, 1030, 1016, 981, 862, 843, 811, 753, 577; HRMS: 639.241148 ([M − H]^−^) for C_31_H_33_F_6_N_4_O_4_, 641.255701([M + H]^+^) for C_31_H_35_F_6_N_4_O_4_.

 *1-(3-methoxy-4-((3-methyl-4-(2,2,2-trifluoroethoxy)pyridin-2-yl)methoxy)benzyl)-3-(4-methoxyphenyl)-1-(1-methylpiperidin-4-yl)urea* (**9c**), white powder 0.71 g, yield 59%; m.p.: 179.1–180.8; MS:603.2([M + H]^+^); ^1^H NMR (400 MHz, DMSO-*d*_6_) δ 8.33 (d, *J* = 5.7 Hz, 1H), 8.12 (s, 1H), 7.33–7.26 (m, 2H), 7.14 (d, *J* = 5.7 Hz, 1H), 7.04 (d, *J* = 8.3 Hz, 1H), 6.90 (d, *J* = 2.0 Hz, 1H), 6.84–6.74 (m, 3H), 5.11 (s, 2H), 4.91 (q, *J* = 8.7 Hz, 2H), 4.49 (s, 2H), 4.08 (tt, *J* = 11.3, 3.9 Hz, 1H), 3.71 (d, *J* = 1.9 Hz, 6H), 2.80 (d, *J* = 11.0 Hz, 2H), 2.21 (s, 3H), 2.17 (s, 3H), 2.06–1.95 (m, 2H), 1.67 (qd, *J* = 12.1, 3.9 Hz, 2H), 1.54 (d, *J* = 12.0 Hz, 2H). ^13^C NMR (101 MHz, DMSO-*d*_6_) δ 161.81, 156.08, 155.96, 155.07, 149.46, 148.06, 146.87, 133.99, 133.88, 124.27 (q, *J* = 278.0 Hz), 122.70, 122.06, 118.80, 114.19, 113.89, 111.29, 108.08, 71.57, 65.10 (q, *J* = 33.8 Hz), 55.96, 55.59, 55.28, 52.98, 46.03, 45.03, 30.19, 10.38; IR: 3403, 2943, 2836, 2788, 2759, 1642, 1583, 1511, 1467, 1446, 1416, 1373, 1295, 1250, 1220, 1159, 1128, 1034, 1011, 962, 860, 824, 737, 666, 576, 542, 441; HRMS: 637.241006 ([M + Cl]^−^) for C_31_H_37_ClF_3_N_4_O_5_, 603.278881 ([M + H]^+^) for C_31_H_38_F_3_N_4_O_5_.

 *1-(3-methoxy-4-((3-methyl-4-(2,2,2-trifluoroethoxy)pyridin-2-yl)methoxy)benzyl)-1-(1-methylpiperidin-4-yl)-3-(3-(trifluoromethyl)phenyl)urea* (**9d**), white powder 0.75 g, yield 59%; m.p.: 160.4–162.2 °C; MS: 641.2([M + H]^+^); ^1^H NMR (400 MHz, DMSO-*d*_6_) δ 8.69 (s, 1H), 8.33 (d, *J* = 5.7 Hz, 1H), 7.91 (d, *J* = 2.0 Hz, 1H), 7.74 (dd, *J* = 8.2, 2.0 Hz, 1H), 7.46 (t, *J* = 8.0 Hz, 1H), 7.27 (d, *J* = 7.8 Hz, 1H), 7.14 (d, *J* = 5.7 Hz, 1H), 7.04 (d, *J* = 8.3 Hz, 1H), 6.90 (d, *J* = 2.0 Hz, 1H), 6.76 (dd, *J* = 8.3, 2.0 Hz, 1H), 5.11 (s, 2H), 4.91 (q, *J* = 8.7 Hz, 2H), 4.54 (s, 2H), 4.11 (tt, *J* = 12.1, 3.9 Hz, 1H), 3.70 (s, 3H), 2.84 (d, *J* = 11.2 Hz, 2H), 2.20 (s, 6H), 2.05 (d, *J* = 13.2 Hz, 2H), 1.72 (tt, *J* = 12.4, 6.8 Hz, 2H), 1.57 (d, *J* = 11.8 Hz, 2H). ^13^C NMR (101 MHz, DMSO-*d*_6_) δ 161.81, 155.93, 155.63, 149.48, 148.06, 146.94, 141.89, 133.50, 129.78, 129.51 (q, *J* = 32.3, 31.7 Hz), 124.76 (d, *J* = 272.5 Hz), 124.26 (q, *J* = 278.9, 278.5 Hz), 123.84, 118.75, 118.39 (d, *J* = 3.9 Hz), 116.39 (d, *J* = 4.4 Hz), 114.22, 111.25, 108.08, 71.54, 65.10 (q, *J* = 34.6 Hz), 55.94, 55.05, 53.16, 46.04, 45.68, 45.21, 29.82, 10.35; IR: 3385, 2940, 2839, 2791, 2771, 2735, 1645, 1583, 1513, 1493, 1468, 1444, 1376, 1326, 1247, 1222, 1152, 1122, 1030, 1000, 972, 909, 835, 788, 749, 701, 667, 540, 458; HRMS: 639.241148 ([M − H]^−^) for C_31_H_33_F_6_N_4_O_4_, 641.255701 ([M + H]^+^) for C_31_H_35_F_6_N_4_O_4_.

 *3-(3-chloro-4-fluorophenyl)-1-(3-methoxy-4-((3-methyl-4-(2,2,2-trifluoroethoxy)pyridin-2-yl)methoxy)benzyl)-1-(1-methylpiperidin-4-yl)urea* (**9e**), white powder 0.69 g, yield 55%; m.p.: 176.2–177.7 °C; MS:625.2([M + H]^+^); ^1^H NMR (400 MHz, DMSO-*d*_6_) δ 8.54 (s, 1H), 8.33 (d, *J* = 5.6 Hz, 1H), 7.74 (dd, *J* = 6.9, 2.6 Hz, 1H), 7.41 (ddd, *J* = 9.2, 4.4, 2.6 Hz, 1H), 7.27 (t, *J* = 9.1 Hz, 1H), 7.14 (d, *J* = 5.7 Hz, 1H), 7.04 (d, *J* = 8.3 Hz, 1H), 6.89 (d, *J* = 2.0 Hz, 1H), 6.75 (dd, *J* = 8.2, 1.9 Hz, 1H), 5.11 (s, 2H), 4.91 (q, *J* = 8.7 Hz, 2H), 4.51 (s, 2H), 4.08 (tt, *J* = 12.1, 3.9 Hz, 1H), 3.71 (s, 3H), 2.83 (d, *J* = 11.2 Hz, 2H), 2.20 (d, *J* = 2.9 Hz, 6H), 2.05 (d, *J* = 11.7 Hz, 2H), 1.71 (qd, *J* = 12.3, 3.7 Hz, 2H), 1.59–1.51 (m, 2H). ^13^C NMR (101 MHz, DMSO-*d*_6_) δ 161.81, 155.93, 155.62, 152.83 (d, *J* = 240.9 Hz), 149.46, 148.06, 146.93, 138.30, 133.52, 124.27 (q, *J* = 277.7 Hz), 122.06, 121.79, 120.59 (d, *J* = 6.5 Hz), 119.01 (d, *J* = 18.2 Hz), 118.75, 116.71 (d, *J* = 21.4 Hz), 114.20, 111.26, 108.08, 71.54, 65.10 (q, *J* = 34.6 Hz), 55.97, 55.09, 53.16, 45.77, 45.19, 29.89, 10.37; IR: 3403, 3365, 2940, 2881, 2822, 1702, 1598, 1515, 1448, 1407, 1370, 1319, 1260, 1217, 1151, 1107, 1063, 965, 906, 842, 785, 713, 593, 509, 455; HRMS: 623.205307 ([M − H]^−^) for C_30_H_33_ClF_4_N_4_O_4_, 625.219923 ([M + H]^+^) for C_30_H_35_ClF_4_N_4_O_4_.

 *1-(3-methoxy-4-((3-methyl-4-(2,2,2-trifluoroethoxy)pyridin-2-yl)methoxy)benzyl)-1-(1-methylpiperidin-4-yl)-3-(3-nitrophenyl)urea* (**9f**), yellow powder 0.79 g, yield 64%; m.p.: 157.5–159.1 °C; MS:618.2([M + H]^+^); ^1^H NMR (400 MHz, DMSO-*d*_6_) δ 8.86 (s, 1H), 8.47 (t, *J* = 2.3 Hz, 1H), 8.33 (d, *J* = 5.7 Hz, 1H), 7.92 (dd, *J* = 8.2, 2.0 Hz, 1H), 7.79 (dd, *J* = 8.1, 2.2 Hz, 1H), 7.51 (t, *J* = 8.2 Hz, 1H), 7.13 (d, *J* = 5.7 Hz, 1H), 7.04 (d, *J* = 8.3 Hz, 1H), 6.91 (d, *J* = 2.0 Hz, 1H), 6.76 (dd, *J* = 8.3, 2.0 Hz, 1H), 5.10 (s, 2H), 4.91 (q, *J* = 8.7 Hz, 2H), 4.55 (s, 2H), 4.10 (tt, *J* = 11.9, 4.0 Hz, 1H), 3.71 (s, 3H), 2.80 (d, *J* = 11.0 Hz, 2H), 2.20 (s, 3H), 2.16 (s, 3H), 2.00 (t, *J* = 11.3 Hz, 2H), 1.71 (qd, *J* = 12.1, 3.8 Hz, 2H), 1.61–1.52 (m, 2H); ^13^C NMR (101 MHz, DMSO-*d*_6_) δ 161.80, 155.93, 155.52, 149.48, 148.28, 148.06, 146.95, 142.46, 133.45, 129.97, 126.24, 124.27 (q, *J* = 277.6 Hz), 122.06, 118.74, 116.58, 114.26, 114.21, 111.25, 108.07, 71.54, 65.10 (q, *J* = 34.4 Hz), 55.97, 55.21, 53.48, 46.01, 45.24, 30.08, 10.37; IR: 3366, 2940, 2842, 2781, 1657, 1585, 1512, 1467, 1426, 1376, 1343, 1248, 1222, 1161, 1131, 1032, 1011, 967, 861, 824, 737, 667, 584, 454; HRMS: 616.238842 ([M − H]^−^) for C_30_H_33_F_3_N_5_O_6_, 618.253395 ([M + H]^+^) for C_30_H_35_F_3_N_5_O_6_.

 *3-(4-ethoxybenzyl)-1-(3-methoxy-4-((3-methyl-4-(2,2,2-trifluoroethoxy)pyridin-2-yl)methoxy)benzyl)-1-(1-methylpiperidin-4-yl)urea* (**9g**), white powder 0.41 g, yield 33%; m.p.: 119.0–121.2 °C; MS 631.3([M + H]^+^); ^1^H NMR (400 MHz, DMSO-*d*_6_) δ 8.34 (d, *J* = 5.7 Hz, 1H), 7.13 (dd, *J* = 15.2, 7.1 Hz, 3H), 7.01 (d, *J* = 8.3 Hz, 1H), 6.84–6.75 (m, 4H), 6.72 (dd, *J* = 8.3, 2.0 Hz, 1H), 5.11 (s, 2H), 4.92 (q, *J* = 8.7 Hz, 2H), 4.36 (s, 2H), 4.18 (d, *J* = 5.6 Hz, 2H), 3.98 (q, *J* = 6.9 Hz, 3H), 3.63 (s, 3H), 2.79 (d, *J* = 11.0 Hz, 2H), 2.21 (s, 3H), 2.17 (s, 3H), 2.00 (t, *J* = 11.8 Hz, 2H), 1.61 (qd, *J* = 12.2, 3.8 Hz, 2H), 1.48 (d, *J* = 9.9 Hz, 2H), 1.32 (d, *J* = 7.0 Hz, 3H). ^13^C NMR (101 MHz, DMSO-*d*_6_) δ 161.82, 157.98, 157.62, 155.99, 149.46, 148.06, 146.82, 134.24, 133.60, 128.63, 124.28 (q, *J* = 277.9 Hz), 122.08, 118.80, 114.39, 114.08, 111.14, 108.09, 71.61, 65.12 (q, *J* = 34.4 Hz), 63.37, 55.79, 55.18, 52.47, 45.79, 44.88, 43.52, 29.99, 15.12, 10.38; IR: 3348, 2937, 2881, 2837, 2793, 1612, 1584, 1509, 1477, 1447, 1395, 1374, 1292, 1253, 1162, 1131, 1033, 1003, 971, 916, 804, 772, 577; HRMS: 665.272306 ([M + Cl]^−^) for C_33_H_41_ClF_3_N_4_O_5_, 631.310182 ([M + H]^+^) for C_33_H_42_F_3_N_4_O_5_.

### 3.2. Biological Evaluation

#### 3.2.1. Antiproliferative Activity Assays

The antiproliferative activities of target compounds were determined using a standard MTT assay [24,25,26,27]. Exponentially growing cells A549 (1.5 × 10^3^ cells/well), MCF-7 (2.2 × 10^3^ cells/well), HCT-116 (800 cells/well), PC-3 (2.0 × 10^3^ cells/well) and HL7702 (5.0 × 10^3^ cells/well) were seeded into 96-well plates and incubated for 24 h to allow the cells to attach. Then, a fresh medium containing various concentrations of the candidate compounds was added to each well. The cells were then incubated for 96 h, thereafter MTT assays were performed, and cell viability was assessed at 570 nm by a microplate reader (ThermoFisher Scientific (Shanghai) Instrument Co., Ltd., Shanghai, China). The optical densities (OD) at 570 nm were measured, and the IC_50_ of the target compounds was calculated by using GraphPad Prism 5.0 software to perform nonlinear fitting with the cell survival rate under different concentrations of the compounds.

#### 3.2.2. Cell Cycle Analysis

As for the flow cytometric analysis of DNA content, 1 × 10^5^ MCF-7 cells in exponential growth were treated with different concentrations of compound **9b** for 24 h. After an incubation period, the cells were collected, centrifuged, and fixed with ice-cold ethanol (70%). The cells were then treated with buffer containing RNAse A and 0.1% Triton X-100 and then stained with the propidium iodide (PI). The samples were analyzed on a flow cytometer (Becton, Dickinson, Franklin Lakes, NJ, USA) [28]. Data were analyzed using Flowjo software v9.0.

## 4. Conclusions

In summary, based on the structure of sorafenib and lansoprazole, 16 target *N*-aryl-*N*’-arylmethylurea derivatives were designed with molecular hybridization and synthesized, and their antiproliferative activities were assayed. The target compounds **9b** and **9d** have shown excellent antiproliferative activities against all four kinds of tumor cell lines (non-small cell lung cancer A549, breast cancer MCF-7, colon cancer HCT116, prostate cancer PC-3). All target compounds have demonstrated weak cytotoxic activities against human liver normal cell line HL7702. The biological assay results showed these target compounds with the 1-methylpiperidin-4-yl group on the 3-position of urea in the target compounds and substituents containing fluorine atoms on the phenyl ring exhibit potently antiproliferative activities. The cell cycle evaluation has shown that compound **9b** could cause an obvious G2/M arrest in a concentration-dependent manner.

## Figures and Tables

**Table 1:** The chemical structures and inhibitory activities of the target compounds.

No.	Structure		IC_50_(μM)			
		A549	MCF7	HCT116	PC3	HL7702
**8a**	** 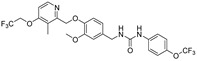 **	5.30 ± 1.45	>50	7.25 ± 0.87	>50	>50
**8b**	** 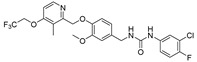 **	17.65 ± 5.65	10.98 ± 1.68	9.33 ± 1.38	29.13 ± 5.81	>50
**8c**	** 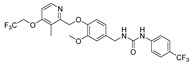 **	4.88 ± 1.94	>50	11.38 ± 3.28	>50	>50
**8d**	** 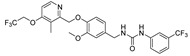 **	26.47 ± 5.66	>50	9.44 ± 1.22	>50	>50
**8e**	** 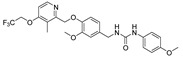 **	29.80 ± 5.09	>50	28.81 ± 3.11	>50	>50
**8f**	** 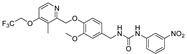 **	12.40 ± 0.60	13.26 ± 2.27	13.35 ± 2.78	15.87 ± 0.73	>50
**8g**	** 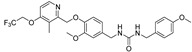 **	>50	>50	7.84 ± 1.40	>50	>50
**8h**	** 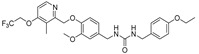 **	10.93 ± 2.02	>50	23.31 ± 3.12	>50	>50
**8i**	** 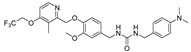 **	9.85 ± 4.40	>50	10.88 ± 2.85	>50	>50
**9a**	** 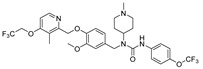 **	17.53 ± 2.95	2.59 ± 0.29	4.41 ± 0.14	4.10 ± 0.19	>50
**9b**	** 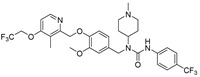 **	4.93 ± 0.46	2.56 ± 0.07	2.90 ± 0.16	3.36 ± 0.17	>50
**9c**	** 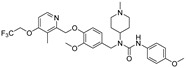 **	15.76 ± 1.51	4.65 ± 0.73	12.90 ± 1.59	12.35 ± 1.75	>50
**9d**	** 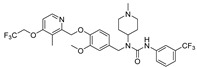 **	3.17 ± 0.22	2.63 ± 0.08	2.56 ± 0.26	3.62 ± 0.27	>50
**9e**	** 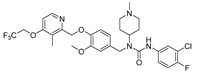 **	5.48 ± 4.36	2.56 ± 0.16	5.82 ± 0.21	4.53 ± 0.33	>50
**9f**	** 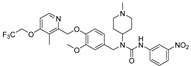 **	6.09 ± 0.29	3.18 ± 0.30	5.18 ± 0.33	7.39 ± 0.63	>50
**9g**	** 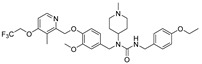 **	6.04 ± 0.41	4.23 ± 0.47	2.93 ± 0.26	6.25 ± 0.43	>50
**sorafenib**		6.16 ± 0.46	3.54 ± 0.19	3.88 ± 0.36	5.26 ± 0.46	>50

## Data Availability

The data presented in this study are available on request from the corresponding authors.

## Supplementary Materials

The following are available online, ^1^H-NMR, ^13^C-NMR, ESI-MS and HRMS of the target compounds (Figures S1–S80).

## Sample Availability

Samples of all target compounds are available from the authors.
