# Supplementary material for: Design, Synthesis and Anticancer Activity of a New Series of N-aryl-N′-[4-(pyridin-2-ylmethoxy)benzyl]urea Derivatives
Source: Molecules. 2021 Jun 8;26(12):3496. doi: 10.3390/molecules26123496 (PMC8226862; doi:10.3390/molecules26123496)
Supplement: Supplementary file 1 [file molecules-26-03496-s001.zip › molecules-1214842-supplementary.pdf]

molecules

Supplementary Materials

Design, synthesis and anticancer activity of a new series of N-aryl-N'-[4-(pyridin-2-ylmethoxy)benzyl]urea derivatives

Shicheng Hou [1], Shishao Liang [1], Chao Zhang [1], Yingmei Han [1], Jianhui Liang [1], Hongyu Hu [1], Xingeng Zhang [1], Chun Hu [1,*], Xiaoping Liu [1,*] and Hong Zhang [2,*]

[1] Key Laboratory of Structure-Based Drug Design & Discovery, Ministry of Education; Shenyang Pharmaceutical University, Shenyang 110016, China; houshicheng2333@163.com (S. Hou); liangshishao2@163.com (S. Liang); zhangchaoylh@126.com (C. Zhang); hanyingmei0613@163.com (Y. Han); liangjianhui1502@163.com (J. Liang); huhongyu64911@163.com (H. Hu); zhangxg96@163.com (X. Zhang); lxp19730107@163.com (X. Liu); chunhu@syphu.edu.cn (C. Hu).

[2] School of Life Science and Biopharmaceutics, Shenyang Pharmaceutical University, Shenyang 110016, China; song0688@sina.com (H. Zhang).

* Correspondences: chunhu@syphu.edu.cn (C. Hu), lxp19730107@163.com (X. Liu) & song0688@sina.com (H. Zhang); Tel.: +86-24-43520246 (C. Hu).

molecules MDPI

Mass Spectrum SmartFormula Report

Analysis Info

Analysis Name	D:\data\HSC-G-19 neg_000001.d	Acquisition Date	3/17/2021 3:20:38 PM
Method	100-1200_neg_20210312	Operator	
Sample Name		Instrument	solariX
Comment			

Acquisition Parameter

Acquisition Mode	Single MS	Acquired Scans	4	Calibration Date	Fri Mar 12 09:12:31 2021
Polarity	Negative	No. of Cell Fills	1	Data Acquisition Size	1048576
Broadband Low Mass	100.3 m/z	No. of Laser Shots	200	Data Processing Size (SI)	2097152
Broadband High Mass	1200.0 m/z	Laser Power	20.0 lp	Apodization	Full-Sine
Source Accumulation	0.000 sec	Laser Shot Frequency	0.001 sec		
Ion Accumulation Time	0.050 sec				

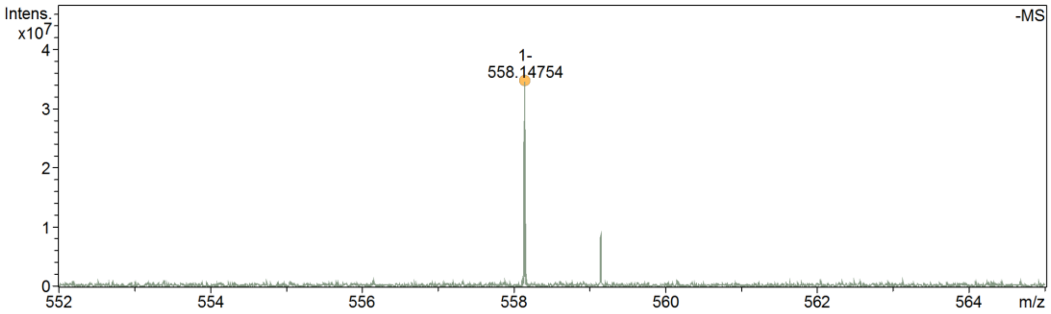

Meas. m/z	#	Ion Formula	Score	m/z	err [ppm]
558.147543	1	C25H22F6N3O5	78.89	558.146914	-1.1

Bruker Compass DataAnalysis 4.4 printed: 3/17/2021 3:24:58 PM by: demo Page 1 of 1

Figure S01：HRMS negative ion spectra of the target compound 8a

molecules MDPI

Mass Spectrum SmartFormula Report

Analysis Info

Analysis Name	D:\data\HSC-G-19 pos_000001.d	Acquisition Date	3/17/2021 3:26:51 PM
Method	100-1200_pos_20210312	Operator	
Sample Name		Instrument	solariX
Comment			

Acquisition Parameter

Acquisition Mode	Single MS	Acquired Scans	2	Calibration Date	Fri Mar 12 09:07:46 2021
Polarity	Positive	No. of Cell Fills	1	Data Acquisition Size	1048576
Broadband Low Mass	100.3 m/z	No. of Laser Shots	500	Data Processing Size (SI)	2097152
Broadband High Mass	1200.0 m/z	Laser Power	20.0 lp	Apodization	Full-Sine
Source Accumulation	0.000 sec	Laser Shot Frequency	0.001 sec		
Ion Accumulation Time	0.050 sec				

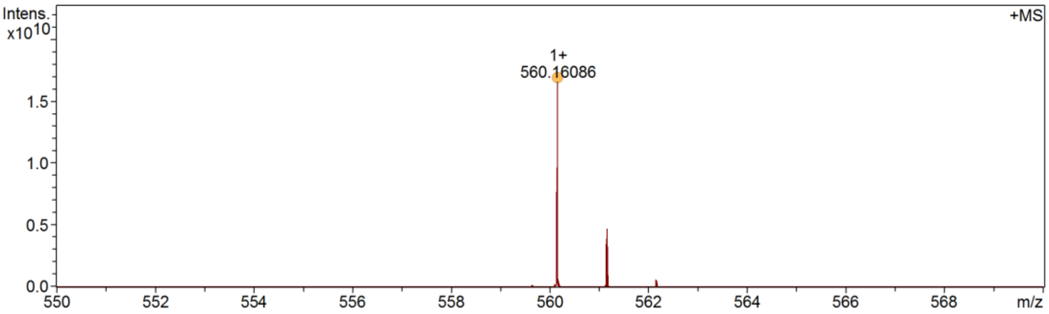

Meas. m/z	#	Ion Formula	Score	m/z	err [ppm]
560.160863	1	C25H24F6N3O5	83.02	560.161467	1.1

Figure S02： HRMS positive ion spectra of the target compound 8a

molecules

MDPI

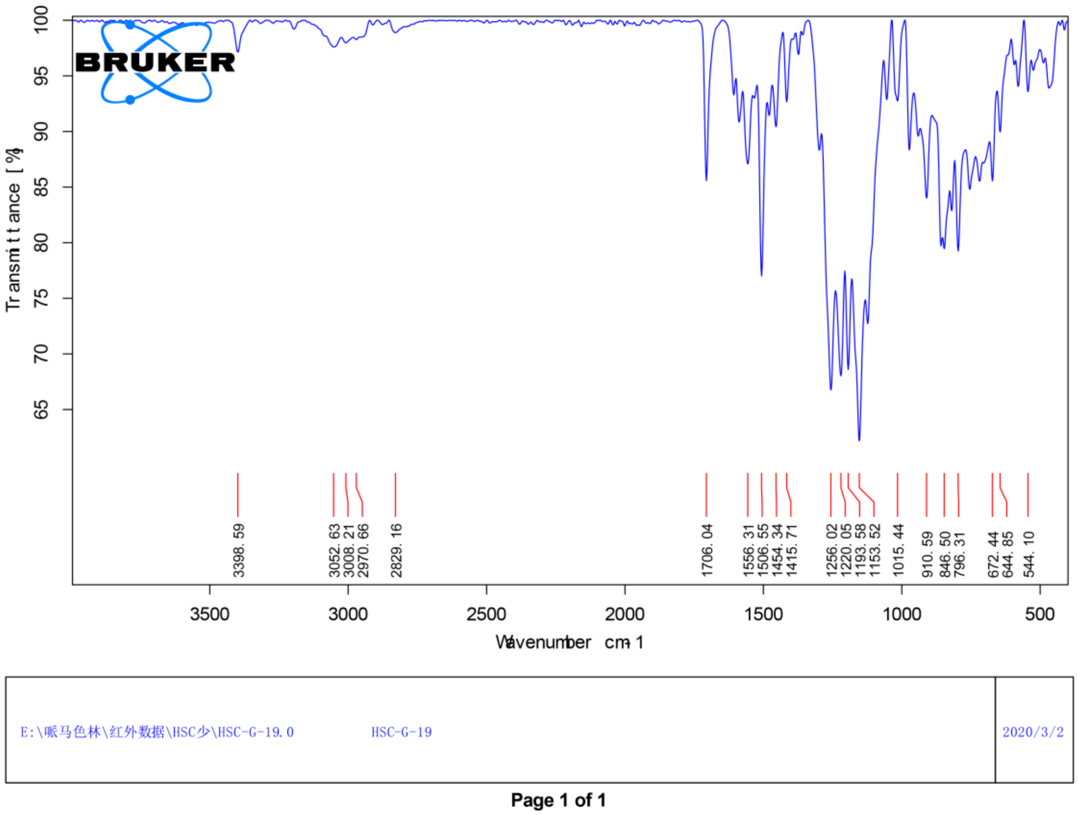

Figure S03: IR spectra of the target compound 8a

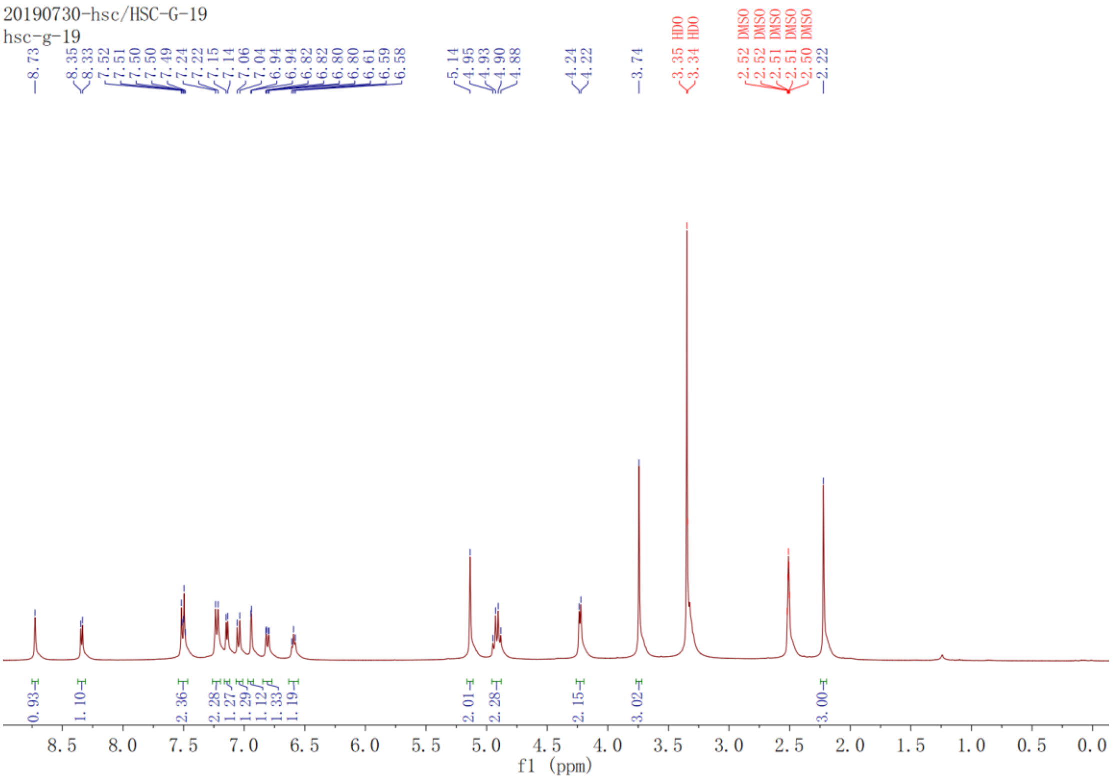

Figure S04: ^{1}H-NMR spectra of the target compound 8a

molecules MDPI

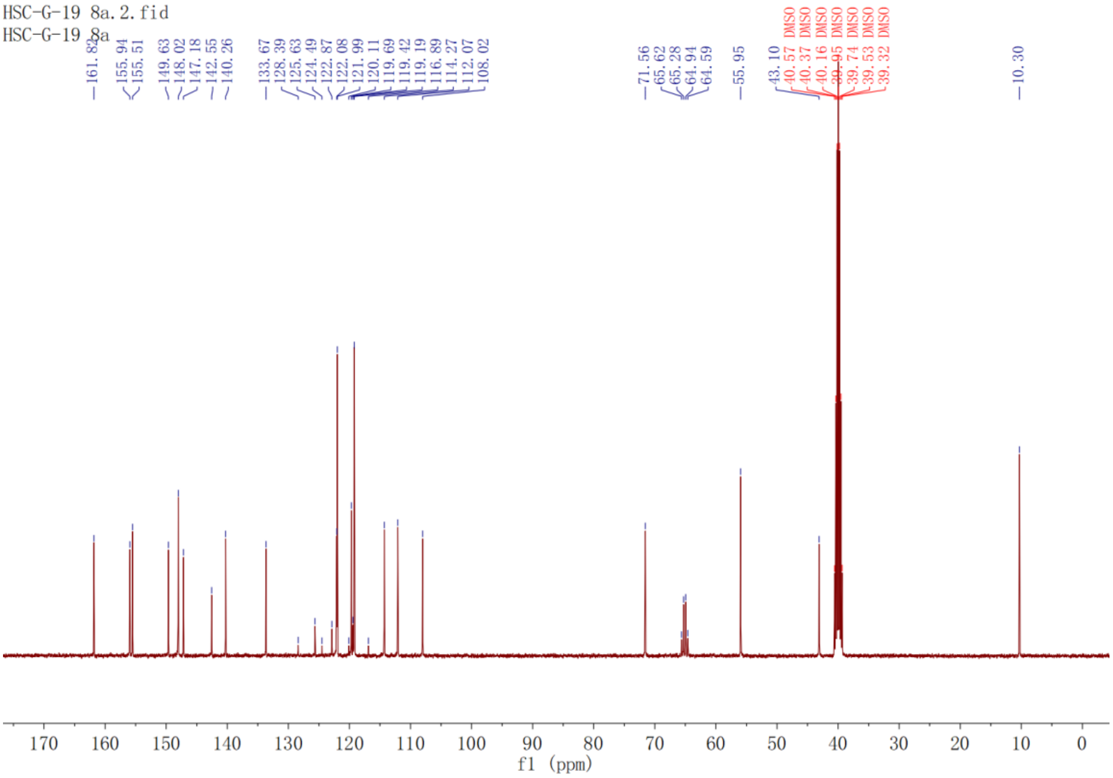

Figure S05: ^{13}C-NMR spectra of the target compound 8a

molecules MDPI

Mass Spectrum SmartFormula Report

Analysis Info

Analysis Name	D:\data\HSC-G-21 neg_000001.d	Acquisition Date	3/17/2021 6:08:31 PM
Method	100-1200_neg_20210312	Operator	
Sample Name		Instrument	solariX
Comment			

Acquisition Parameter

Acquisition Mode	Single MS	Acquired Scans	3	Calibration Date	Fri Mar 12 09:12:31 2021
Polarity	Negative	No. of Cell Fills	1	Data Acquisition Size	1048576
Broadband Low Mass	100.3 m/z	No. of Laser Shots	200	Data Processing Size (SI)	2097152
Broadband High Mass	1200.0 m/z	Laser Power	20.0 lp	Apodization	Full-Sine
Source Accumulation	0.000 sec	Laser Shot Frequency	0.001 sec		
Ion Accumulation Time	0.050 sec				

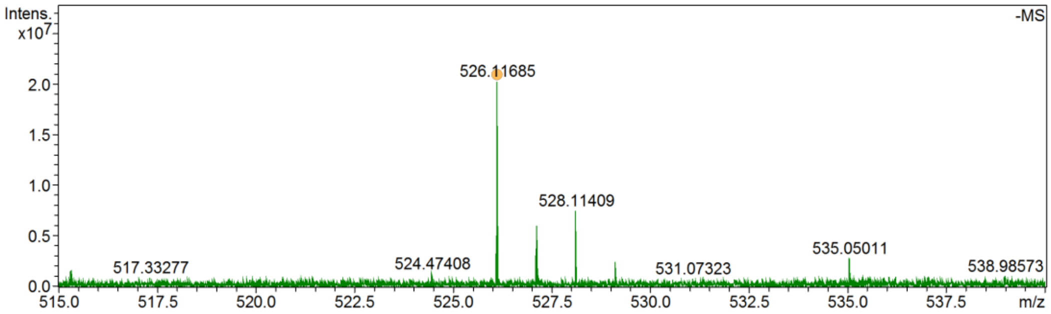

Meas. m/z	#	Ion Formula	Score	m/z	err [ppm]
526.116854	1	C24H21ClF4N3O4	100.00	526.116220	-1.2

Figure S06: HRMS negative ion spectra of the target compound 8b

Mass Spectrum SmartFormula Report

Analysis Info

Analysis Name	D:\data\HSC-G-21 pos_000001.d	Acquisition Date	3/17/2021 6:05:07 PM
Method	100-1200_pos_20210312	Operator	
Sample Name		Instrument	solariX
Comment			

Acquisition Parameter

Acquisition Mode	Single MS	Acquired Scans	2	Calibration Date	Fri Mar 12 09:07:46 2021
Polarity	Positive	No. of Cell Fills	1	Data Acquisition Size	1048576
Broadband Low Mass	100.3 m/z	No. of Laser Shots	500	Data Processing Size (SI)	2097152
Broadband High Mass	1200.0 m/z	Laser Power	20.0 lp	Apodization	Full-Sine
Source Accumulation	0.000 sec	Laser Shot Frequency	0.001 sec		
Ion Accumulation Time	0.050 sec				

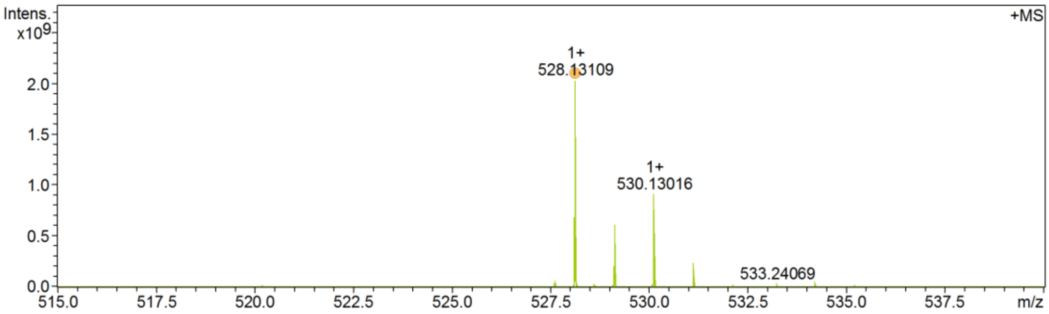

Meas. m/z	#	Ion Formula	Score	m/z	err [ppm]
528.131093	1	C24H23ClF4N3O4	100.00	528.130773	-0.6

Figure S07: HRMS positive ion spectra of the target compound 8b

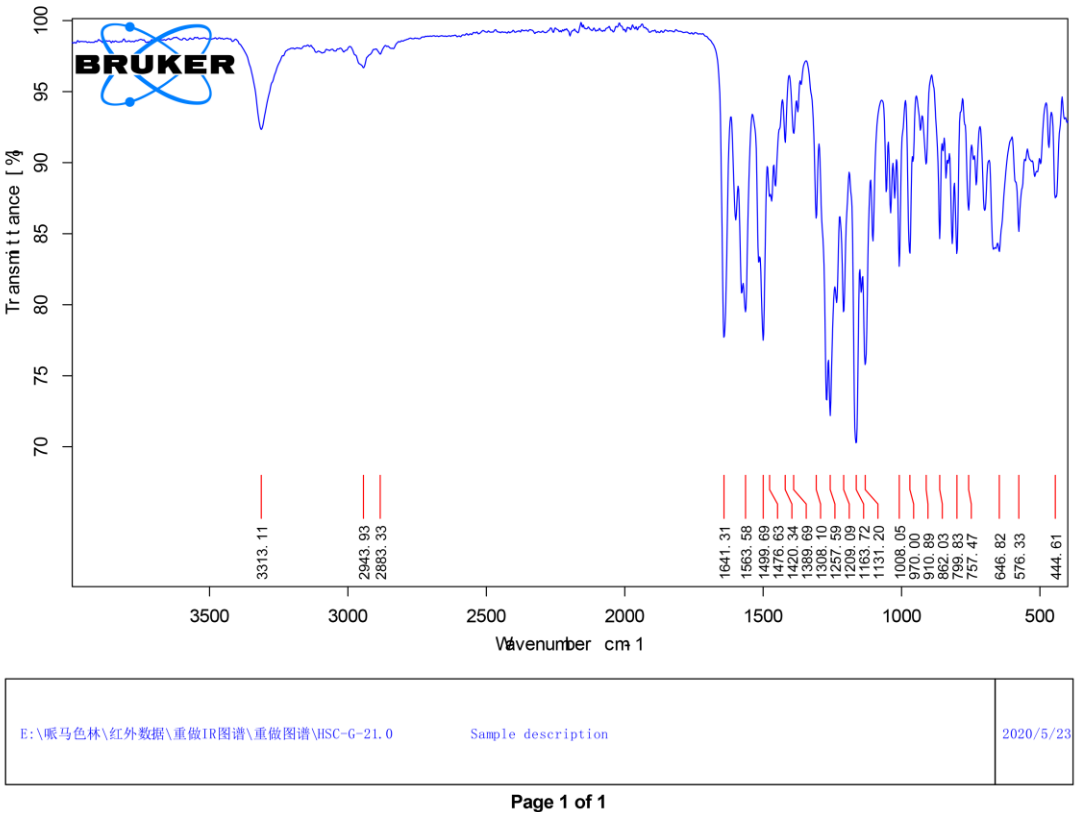

Figure S08: IR spectra of the target compound 8b

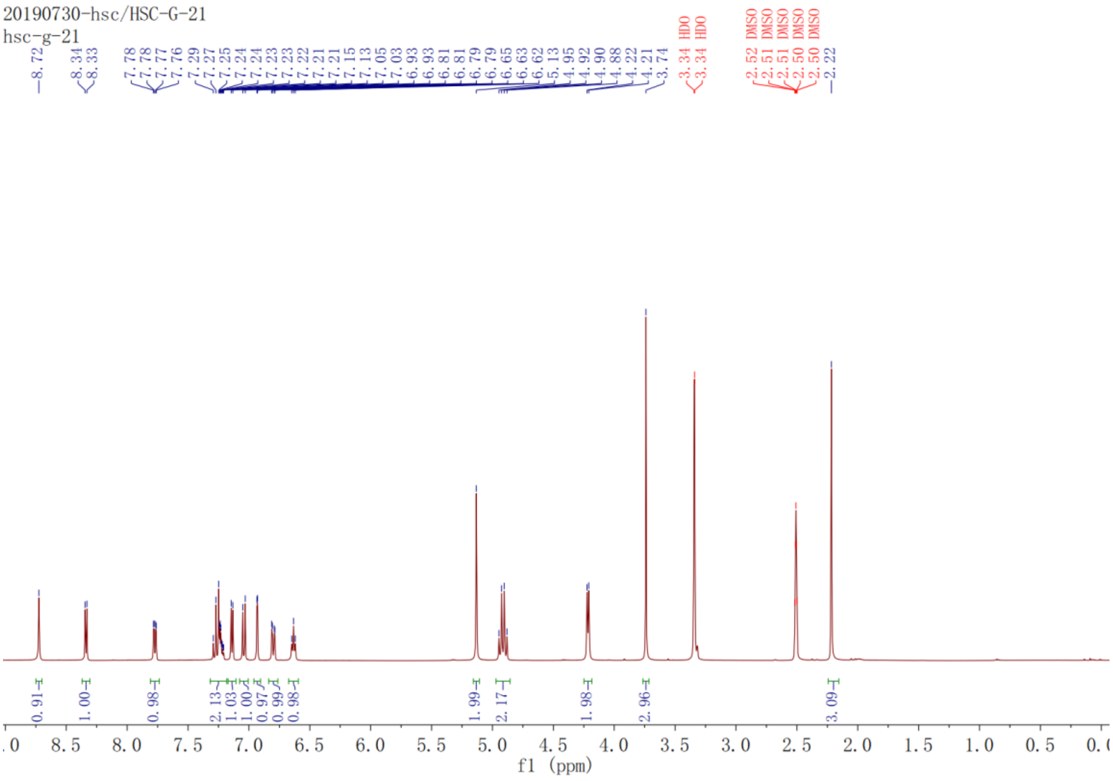

Figure S09: ^{1}H-NMR spectra of the target compound 8b

molecules

MDPI

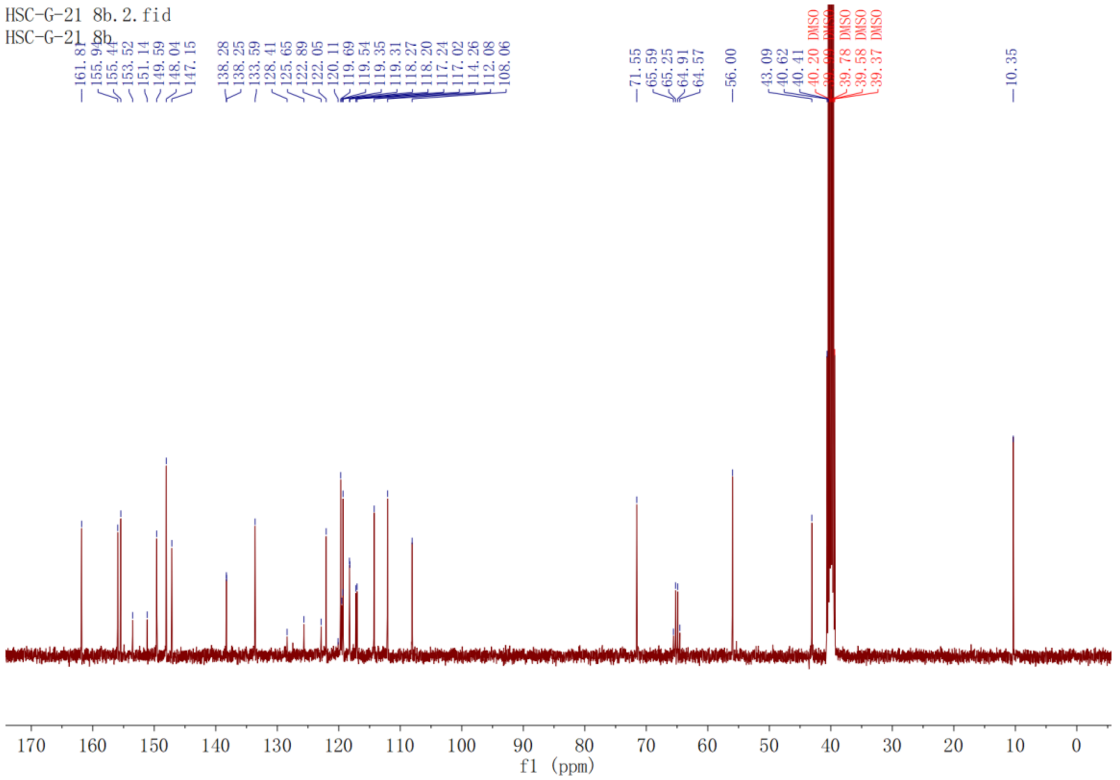

Figure S10: ^{13}C-NMR spectra of the target compound 8b

molecules MDPI

Mass Spectrum SmartFormula Report

Analysis Info

Analysis Name	D:\data\20210317\HSC-G-22 neg_000001.d	Acquisition Date	3/17/2021 6:48:00 PM
Method	100-1200_neg_20210312	Operator	
Sample Name		Instrument	solariX
Comment			

Acquisition Parameter

Acquisition Mode	Single MS	Acquired Scans	3	Calibration Date	Fri Mar 12 09:12:31 2021
Polarity	Negative	No. of Cell Fills	1	Data Acquisition Size	1048576
Broadband Low Mass	100.3 m/z	No. of Laser Shots	200	Data Processing Size (SI)	2097152
Broadband High Mass	1200.0 m/z	Laser Power	20.0 lp	Apodization	Full-Sine
Source Accumulation	0.000 sec	Laser Shot Frequency	0.001 sec		
Ion Accumulation Time	0.050 sec				

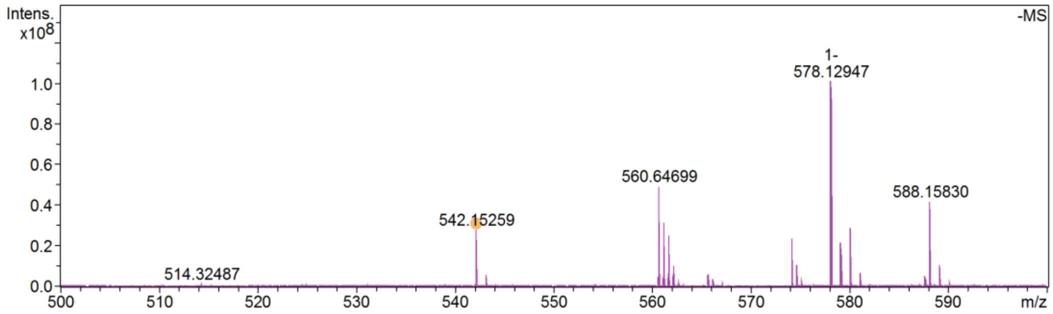

Meas. m/z	#	Ion Formula	Score	m/z	err [ppm]
542.152589	1	C25H22F6N3O4	47.70	542.151999	-1.1

Figure S11: HRMS negative ion spectra of the target compound 8c

Mass Spectrum SmartFormula Report

Analysis Info

Analysis Name	D:\data\20210317\HSC-G-22 pos_000001.d	Acquisition Date	3/17/2021 6:41:59 PM
Method	100-1200_pos_20210312	Operator	
Sample Name		Instrument	solariX
Comment			

Acquisition Parameter

Acquisition Mode	Single MS	Acquired Scans	3	Calibration Date	Fri Mar 12 09:07:46 2021
Polarity	Positive	No. of Cell Fills	1	Data Acquisition Size	1048576
Broadband Low Mass	100.3 m/z	No. of Laser Shots	500	Data Processing Size (SI)	2097152
Broadband High Mass	1200.0 m/z	Laser Power	20.0 lp	Apodization	Full-Sine
Source Accumulation	0.000 sec	Laser Shot Frequency	0.001 sec		
Ion Accumulation Time	0.050 sec				

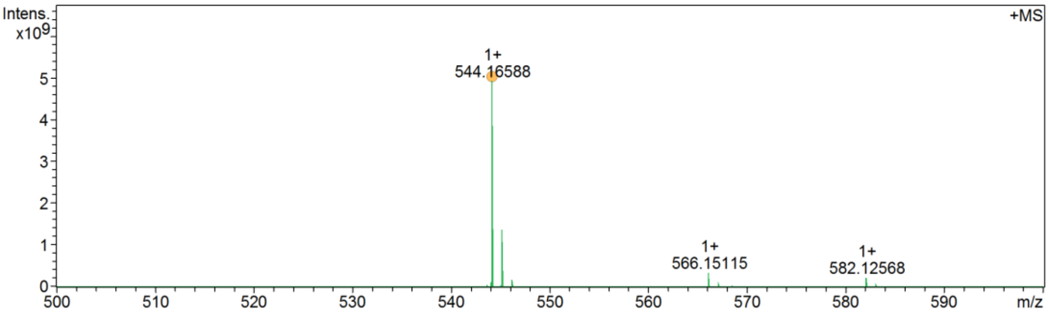

Meas. m/z	#	Ion Formula	Score	m/z	err [ppm]
544.165884	1	C25H24F6N3O4	79.13	544.166552	1.2

Figure S12: HRMS positive ion spectra of the target compound 8c

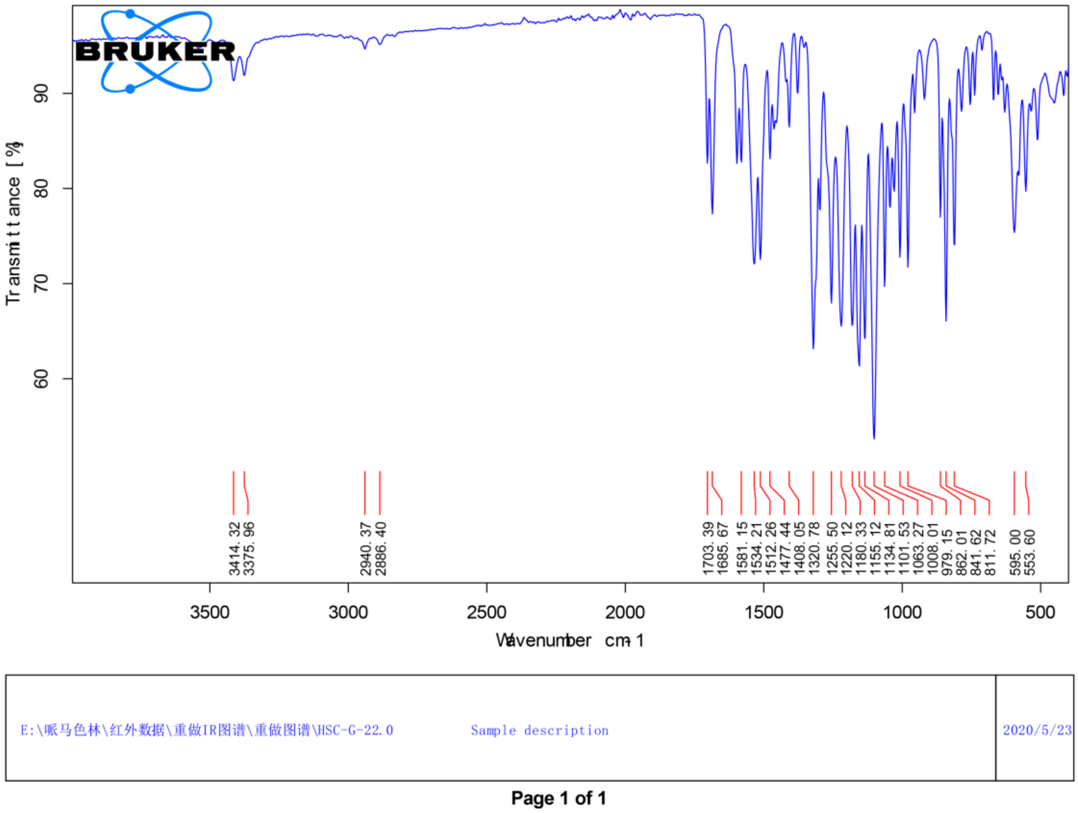

Figure S13: IR spectra of the target compound 8c

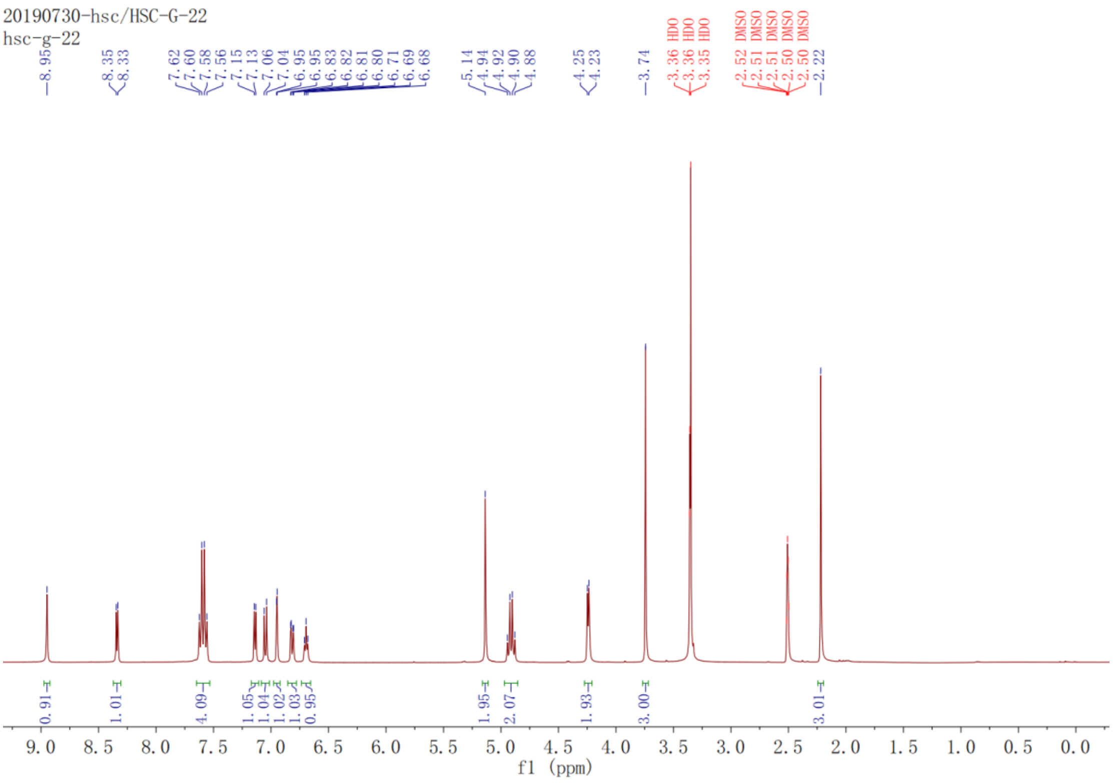

Figure S14: ^{1}H-NMR spectra of the target compound 8c

molecules

MDPI

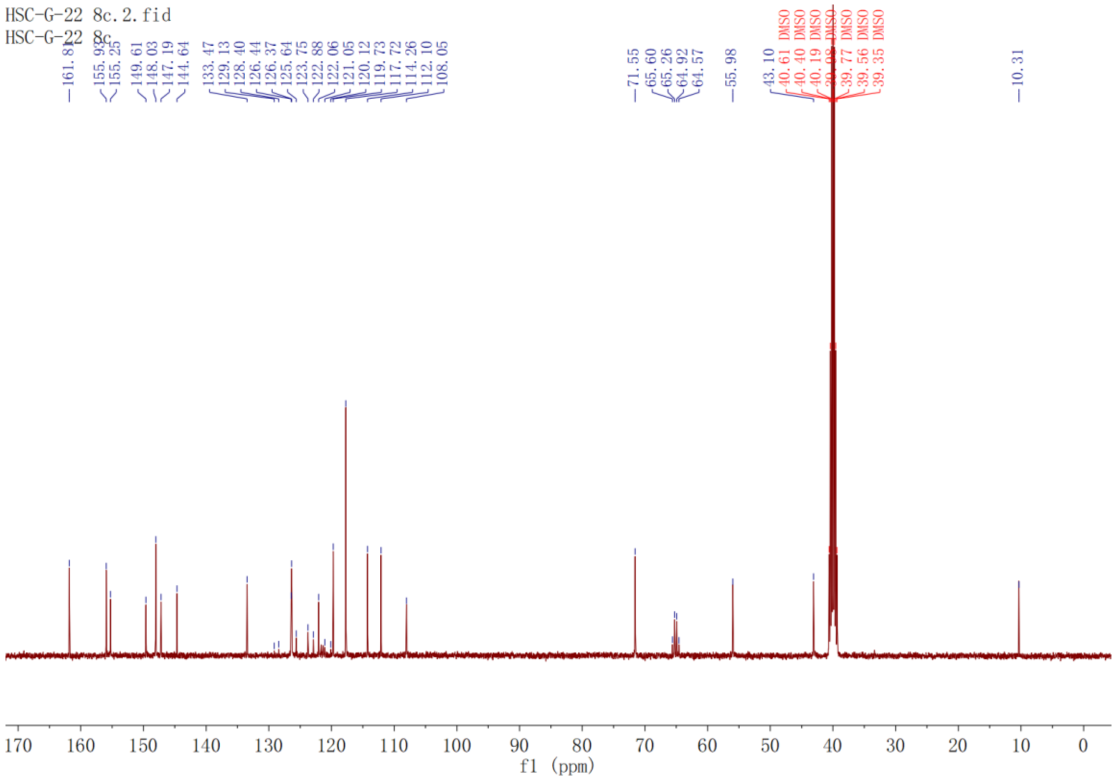

Figure S15: ^{13}C-NMR spectra of the target compound 8c

molecules

Mass Spectrum SmartFormula Report

Analysis Info

Analysis Name	D:\data\20210317\HSC-K-2 neg_000001.d	Acquisition Date	3/17/2021 7:15:20 PM
Method	100-1200_neg_20210312	Operator	
Sample Name		Instrument	solariX
Comment			

Acquisition Parameter

Acquisition Mode	Single MS	Acquired Scans	3	Calibration Date	Fri Mar 12 09:12:31 2021
Polarity	Negative	No. of Cell Fills	1	Data Acquisition Size	1048576
Broadband Low Mass	100.3 m/z	No. of Laser Shots	200	Data Processing Size (SI)	2097152
Broadband High Mass	1200.0 m/z	Laser Power	20.0 lp	Apodization	Full-Sine
Source Accumulation	0.000 sec	Laser Shot Frequency	0.001 sec		
Ion Accumulation Time	0.050 sec				

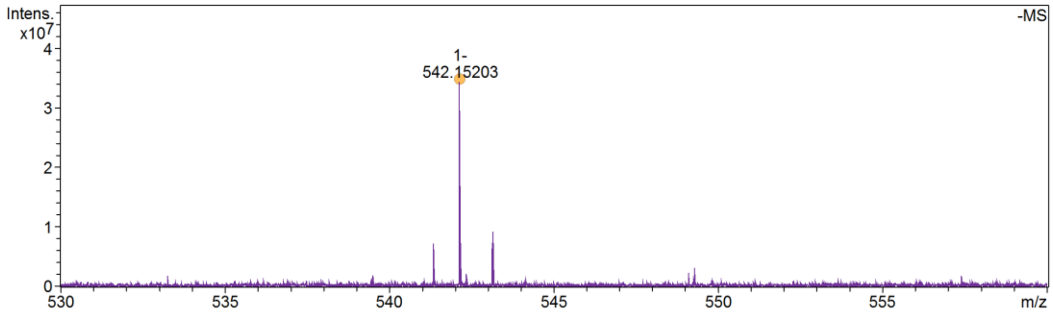

Meas. m/z	#	Ion Formula	Score	m/z	err [ppm]
542.152030	1	C25H22F6N3O4	100.00	542.151999	-0.1

Figure S16; HRSM negative ion spectra of the target compound 8d

molecules MDPI

Mass Spectrum SmartFormula Report

Analysis Info

Analysis Name	D:\data\20210317\HSC-K-2 pos_000002.d	Acquisition Date	3/17/2021 7:12:14 PM
Method	100-1200_pos_20210312	Operator	
Sample Name		Instrument	solariX
Comment			

Acquisition Parameter

Acquisition Mode	Single MS	Acquired Scans	3	Calibration Date	Fri Mar 12 09:07:46 2021
Polarity	Positive	No. of Cell Fills	1	Data Acquisition Size	1048576
Broadband Low Mass	100.3 m/z	No. of Laser Shots	500	Data Processing Size (SI)	2097152
Broadband High Mass	1200.0 m/z	Laser Power	20.0 lp	Apodization	Full-Sine
Source Accumulation	0.000 sec	Laser Shot Frequency	0.001 sec		
Ion Accumulation Time	0.050 sec				

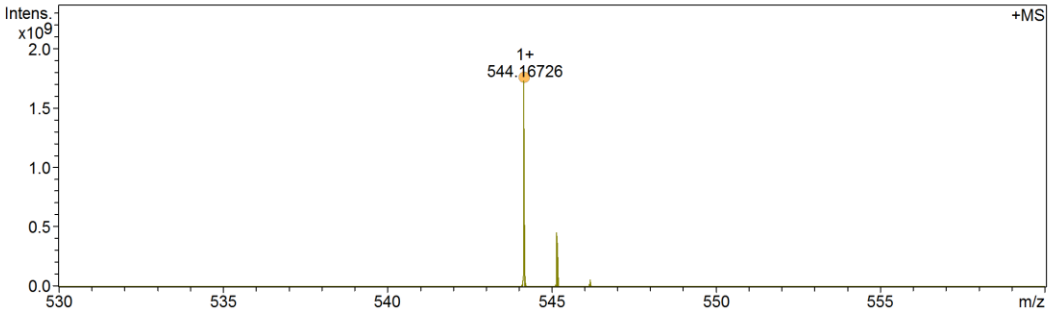

Meas. m/z	#	Ion Formula	Score	m/z	err [ppm]
544.167262	1	C25H24F6N3O4	73.22	544.166552	-1.3

Figure S17: HRMS positive ion spectra of the target compound 8d

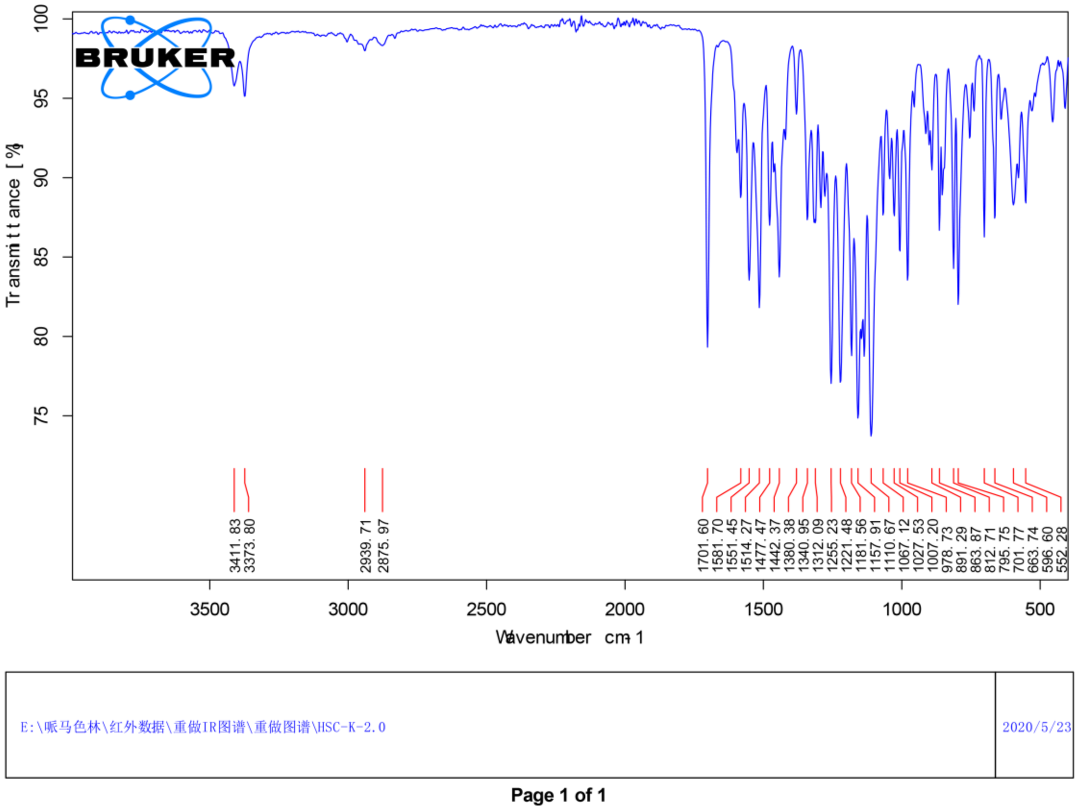

Figure S18: IR spectra of the target compound 8d

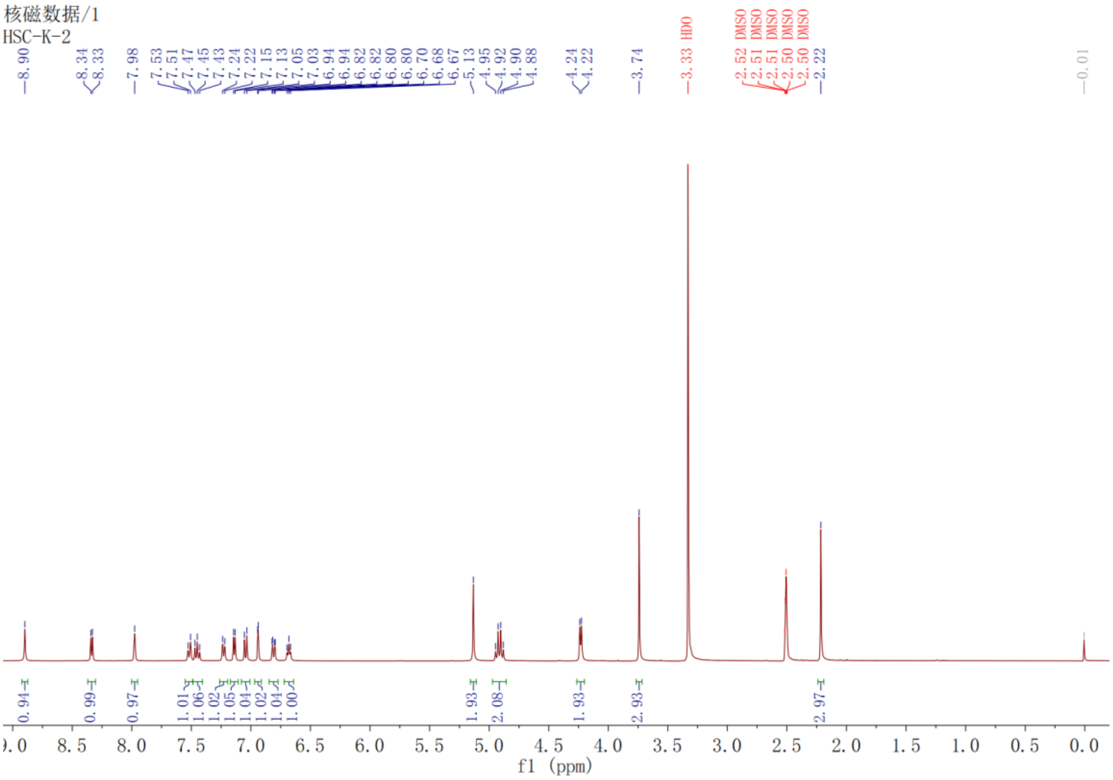

Figure S19: ^{1}H-NMR spectra of the target compound 8d

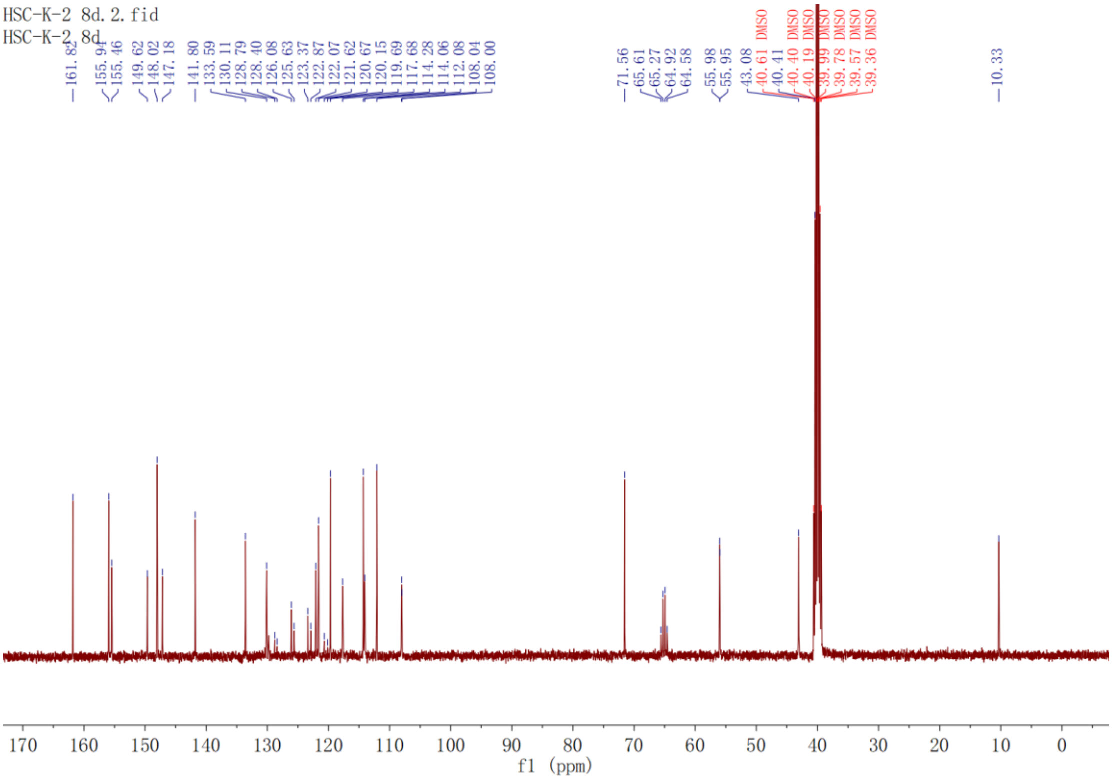

Figure S20: ^{13}C-NMR spectra of the target compound 8d

molecules MDPI

Mass Spectrum SmartFormula Report

Analysis Info

Analysis Name	D:\data\HSC-K-4 neg_000001.d	Acquisition Date	3/17/2021 4:42:02 PM
Method	100-1200_neg_20210312	Operator	
Sample Name		Instrument	solariX
Comment			

Acquisition Parameter

Acquisition Mode	Single MS	Acquired Scans	4	Calibration Date	Fri Mar 12 09:12:31 2021
Polarity	Negative	No. of Cell Fills	1	Data Acquisition Size	1048576
Broadband Low Mass	100.3 m/z	No. of Laser Shots	200	Data Processing Size (SI)	2097152
Broadband High Mass	1200.0 m/z	Laser Power	20.0 lp	Apodization	Full-Sine
Source Accumulation	0.000 sec	Laser Shot Frequency	0.001 sec		
Ion Accumulation Time	0.050 sec				

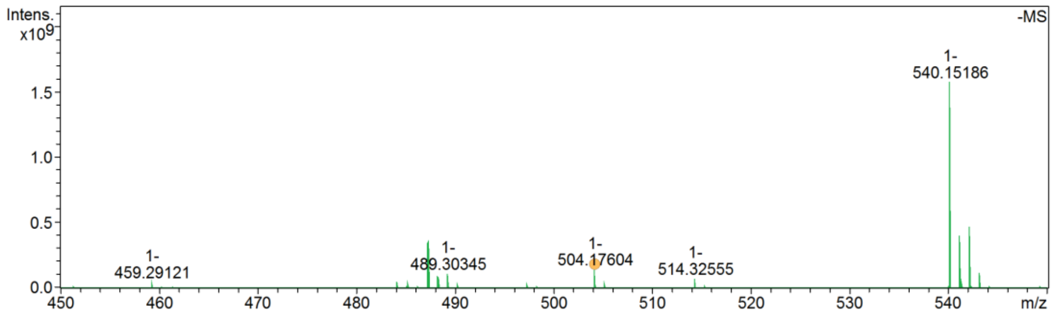

Meas. m/z	#	Ion Formula	Score	m/z	err [ppm]
504.176038	1	C25H25F3N3O5	69.99	504.175179	-1.7

Figure S21: HRMS negative ion spectra of the target compound 8e

molecules MDPI

Mass Spectrum SmartFormula Report

Analysis Info

Analysis Name	D:\data\HSC-K-4 pos_000007.d	Acquisition Date	3/17/2021 4:50:39 PM
Method	100-1200_pos_20210312	Operator	
Sample Name		Instrument	solariX
Comment			

Acquisition Parameter

Acquisition Mode	Single MS	Acquired Scans	6	Calibration Date	Fri Mar 12 09:07:46 2021
Polarity	Positive	No. of Cell Fills	1	Data Acquisition Size	1048576
Broadband Low Mass	100.3 m/z	No. of Laser Shots	500	Data Processing Size (SI)	2097152
Broadband High Mass	1200.0 m/z	Laser Power	20.0 lp	Apodization	Full-Sine
Source Accumulation	0.000 sec	Laser Shot Frequency	0.001 sec		
Ion Accumulation Time	0.050 sec				

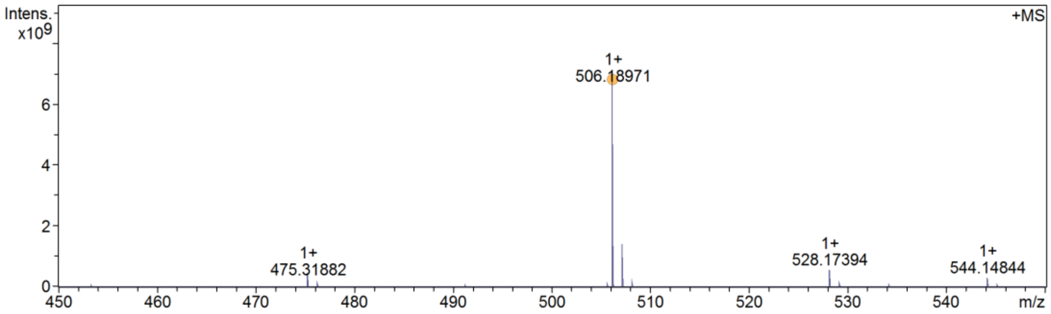

Meas. m/z	#	Ion Formula	Score	m/z	err [ppm]
506.189713	1	C25H27F3N3O5	100.00	506.189732	0.0

Figure S22: HRMS positive ion spectra of the target compound 8e

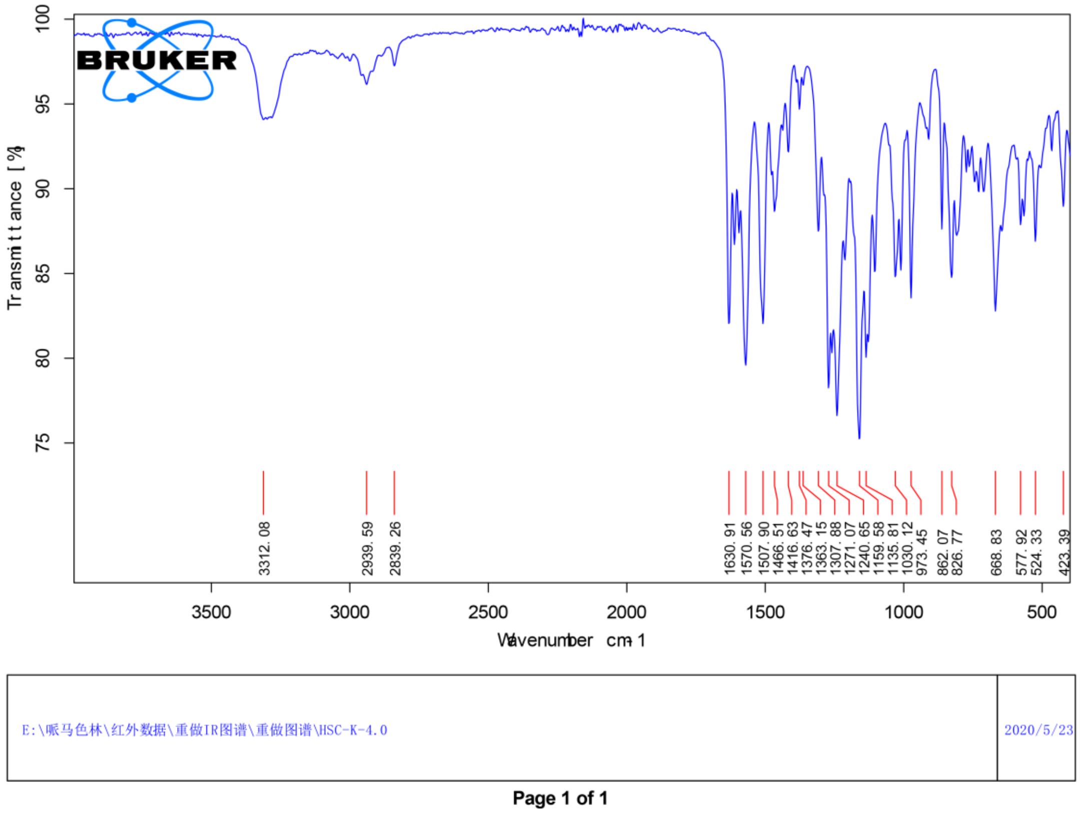

Figure S23: IR spectra of the target compound 8e

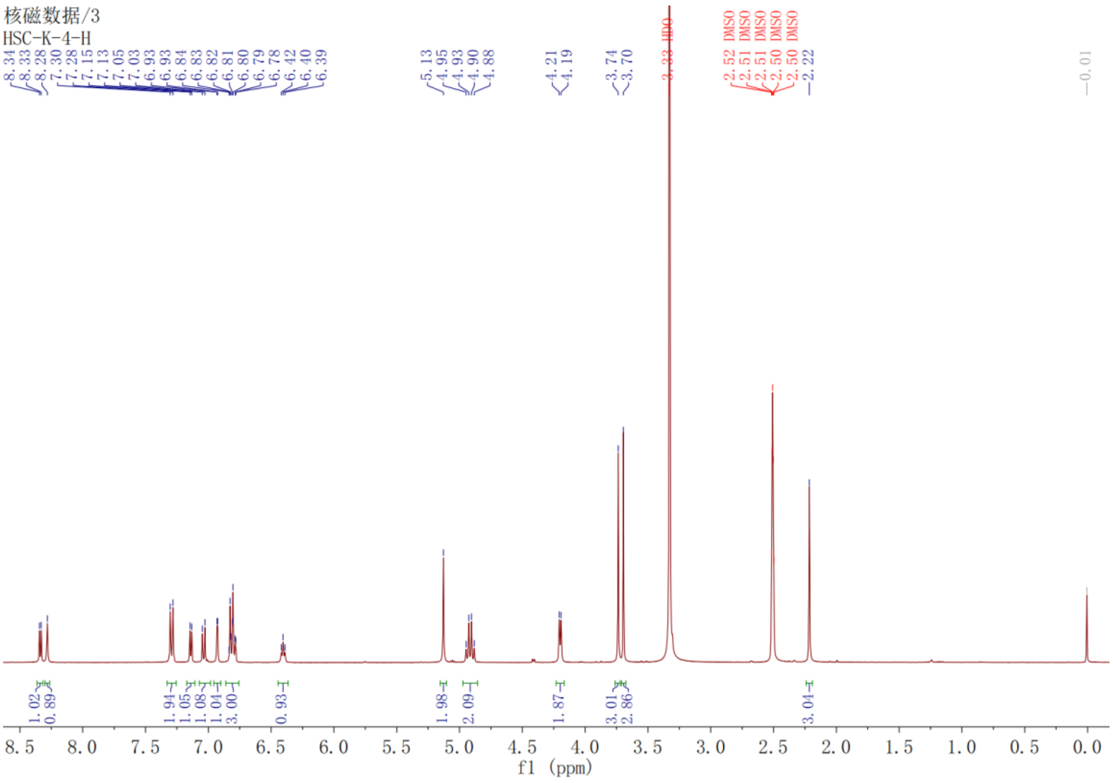

Figure S24: ^{1}H-NMR spectra of the target compound 8e

molecules

MDPI

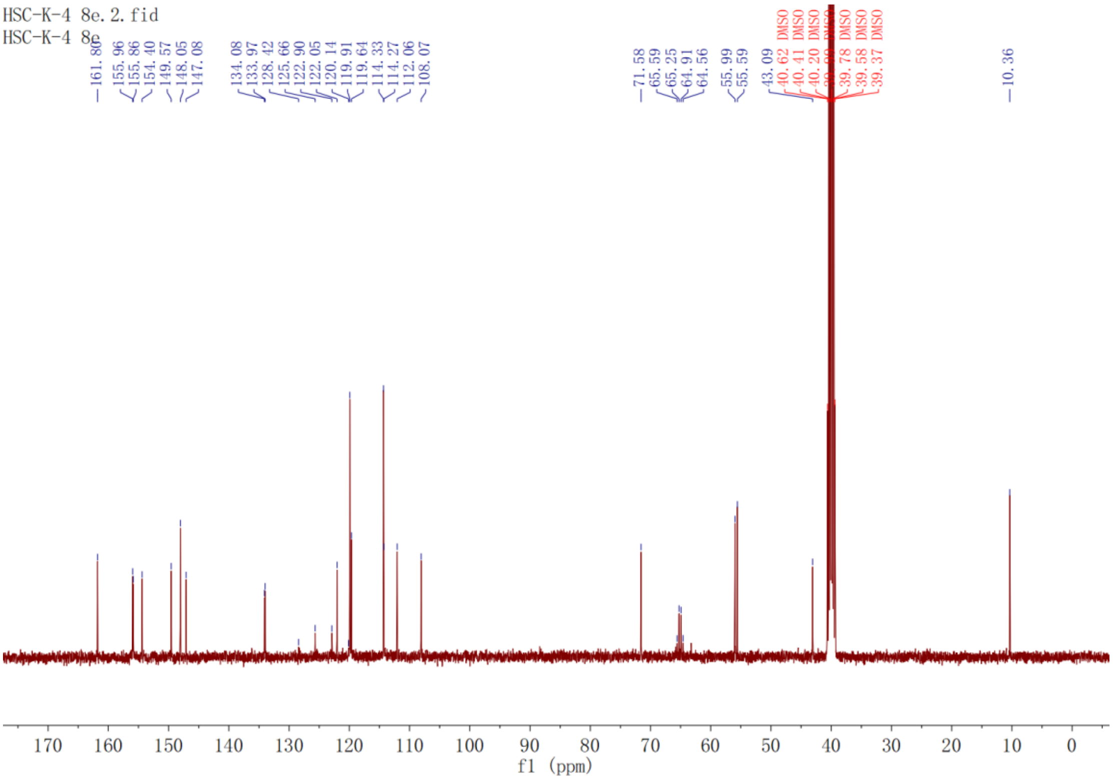

Figure S25: ^{13}C-NMR spectra of the target compound 8e

molecules MDPI

Mass Spectrum SmartFormula Report

Analysis Info

Analysis Name D:\data\HSC-K-5 neg_000001.d
Method 100-1200_neg_20210312
Sample Name
Comment

Acquisition Date 3/17/2021 3:50:59 PM
Operator
Instrument solariX

Acquisition Parameter

Acquisition Mode	Single MS	Acquired Scans	4	Calibration Date	Fri Mar 12 09:12:31 2021
Polarity	Negative	No. of Cell Fills	1	Data Acquisition Size	1048576
Broadband Low Mass	100.3 m/z	No. of Laser Shots	200	Data Processing Size (SI)	2097152
Broadband High Mass	1200.0 m/z	Laser Power	20.0 lp	Apodization	Full-Sine
Source Accumulation	0.000 sec	Laser Shot Frequency	0.001 sec		
Ion Accumulation Time	0.050 sec				

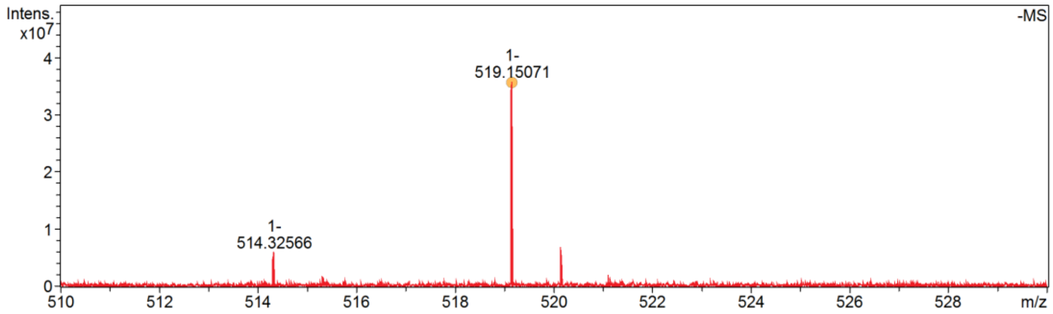

Meas. m/z	#	Ion Formula	Score	m/z	err [ppm]
519.150713	1	C24H22F3N4O6	100.00	519.149693	-2.0

Figure S26: HRMS negative ion spectra of the target compound 8f

molecules

MDPI

Mass Spectrum SmartFormula Report

Analysis Info

Analysis Name	D:\data\HSC-K-5 pos_000006.d	Acquisition Date	3/17/2021 3:59:40 PM
Method	100-1200_pos_20210312	Operator	
Sample Name		Instrument	solariX
Comment			

Acquisition Parameter

Acquisition Mode	Single MS	Acquired Scans	2	Calibration Date	Fri Mar 12 09:07:46 2021
Polarity	Positive	No. of Cell Fills	1	Data Acquisition Size	1048576
Broadband Low Mass	100.3 m/z	No. of Laser Shots	500	Data Processing Size (SI)	2097152
Broadband High Mass	1200.0 m/z	Laser Power	20.0 lp	Apodization	Full-Sine
Source Accumulation	0.000 sec	Laser Shot Frequency	0.001 sec		
Ion Accumulation Time	0.050 sec				

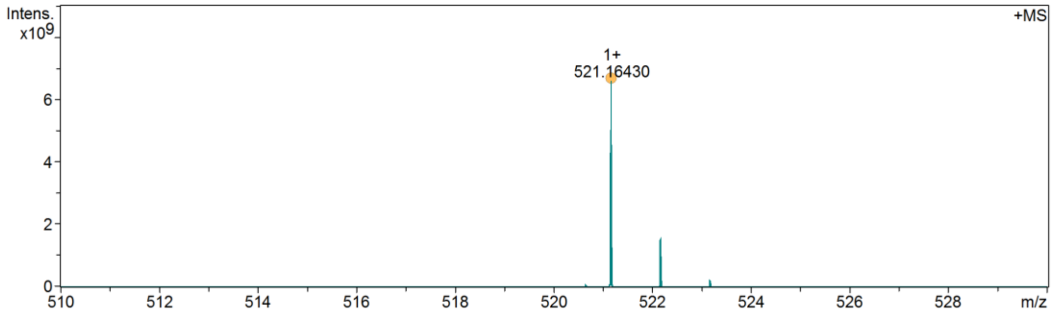

Meas. m/z	#	Ion Formula	Score	m/z	err [ppm]
521.164298	1	C24H24F3N4O6	100.00	521.164246	-0.1

Figure S27: HRMS positive ion spectra of the target compound 8f

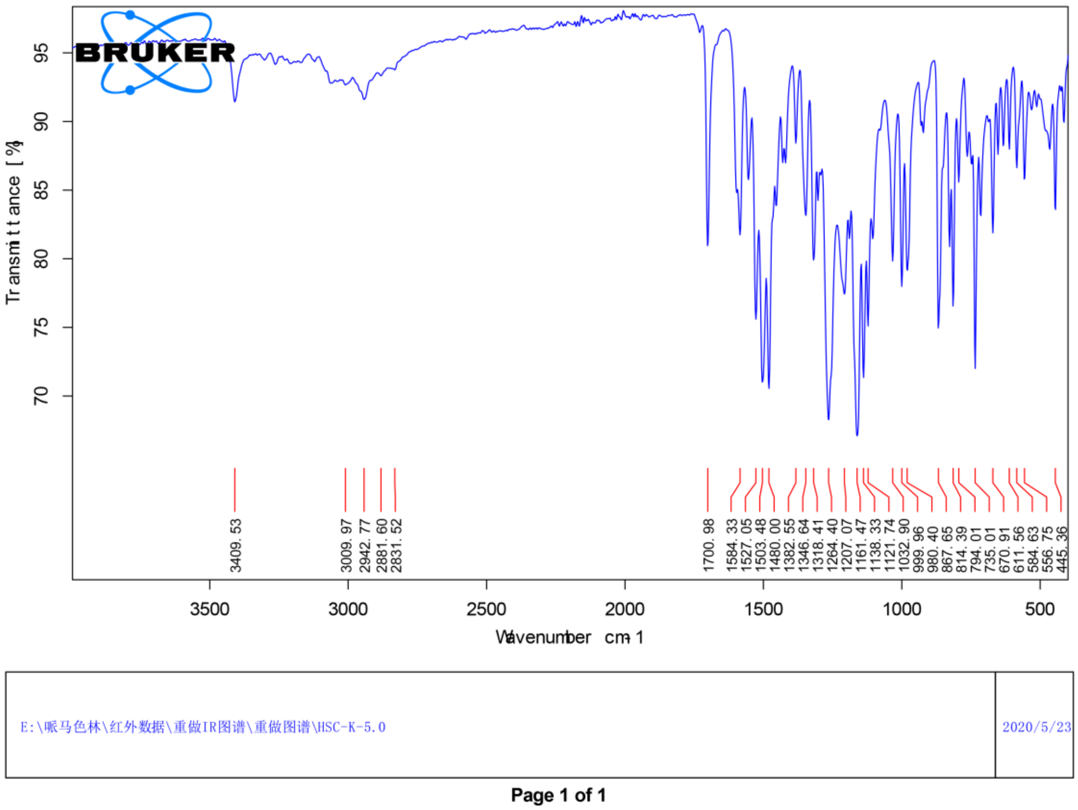

Figure S28: IR spectra of the target compound 8f

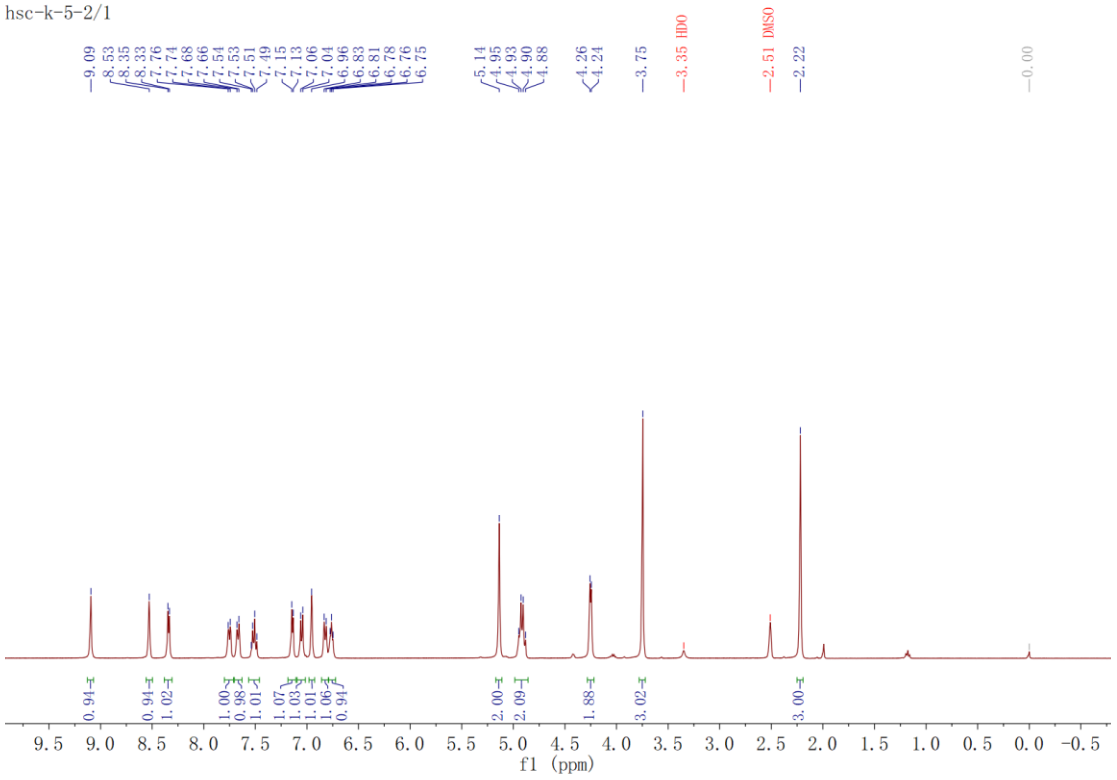

Figure S29: ^{1}H-NMR spectra of the target compound 8f

molecules

MDPI

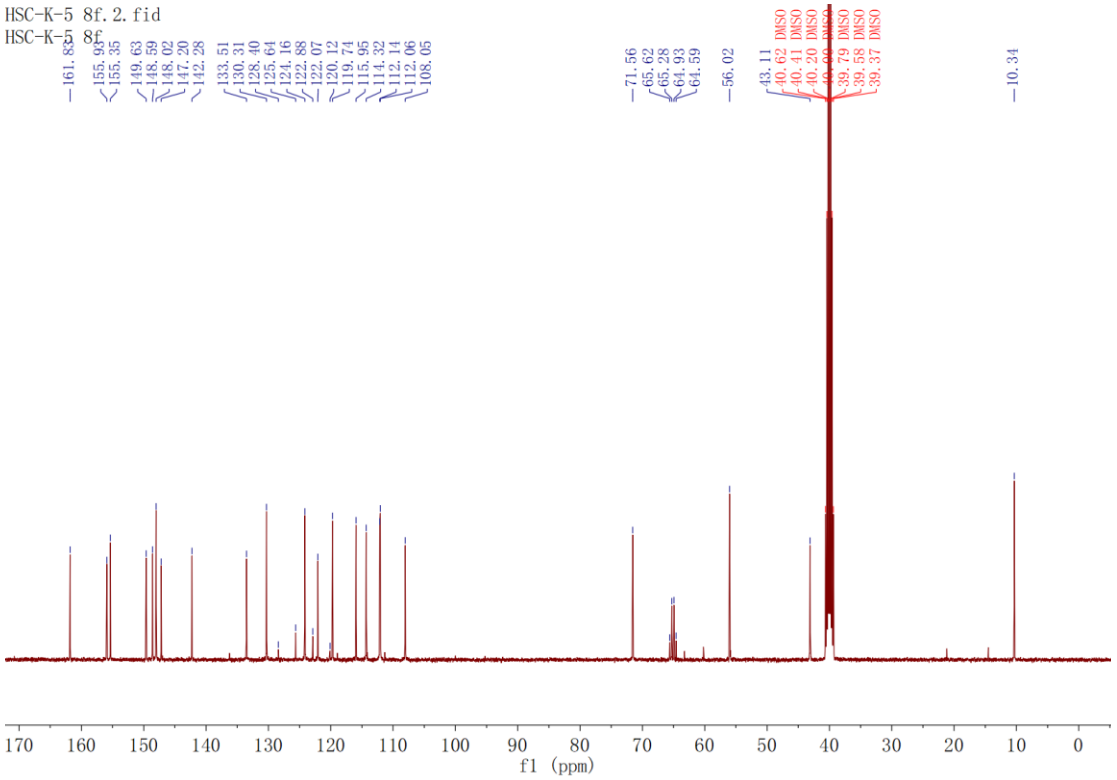

Figure S30: ^{13}C-NMR spectra of the target compound 8f

molecules MDPI

Mass Spectrum SmartFormula Report

Analysis Info

Analysis Name	D:\data\HSC-K-6 neg_000001.d	Acquisition Date	3/17/2021 5:21:07 PM
Method	100-1200_neg_20210312	Operator	
Sample Name		Instrument	solariX
Comment			

Acquisition Parameter

Acquisition Mode	Single MS	Acquired Scans	2	Calibration Date	Fri Mar 12 09:12:31 2021
Polarity	Negative	No. of Cell Fills	1	Data Acquisition Size	1048576
Broadband Low Mass	100.3 m/z	No. of Laser Shots	200	Data Processing Size (SI)	2097152
Broadband High Mass	1200.0 m/z	Laser Power	20.0 lp	Apodization	Full-Sine
Source Accumulation	0.000 sec	Laser Shot Frequency	0.001 sec		
Ion Accumulation Time	0.050 sec				

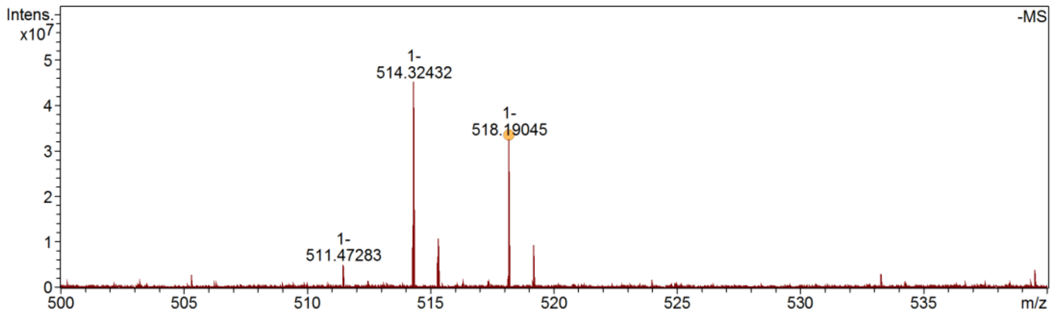

Meas. m/z	#	Ion Formula	Score	m/z	err [ppm]
518.190453	1	C26H27F3N3O5	100.00	518.190829	0.7

Figure S31: HRMS negative ion spectra of the target compound 8g

Mass Spectrum SmartFormula Report

Analysis Info

Analysis Name	D:\data\HSC-K-6 pos_000001.d	Acquisition Date	3/17/2021 5:18:18 PM
Method	100-1200_pos_20210312	Operator	
Sample Name		Instrument	solariX
Comment			

Acquisition Parameter

Acquisition Mode	Single MS	Acquired Scans	2	Calibration Date	Fri Mar 12 09:07:46 2021
Polarity	Positive	No. of Cell Fills	1	Data Acquisition Size	1048576
Broadband Low Mass	100.3 m/z	No. of Laser Shots	500	Data Processing Size (SI)	2097152
Broadband High Mass	1200.0 m/z	Laser Power	20.0 lp	Apodization	Full-Sine
Source Accumulation	0.000 sec	Laser Shot Frequency	0.001 sec		
Ion Accumulation Time	0.050 sec				

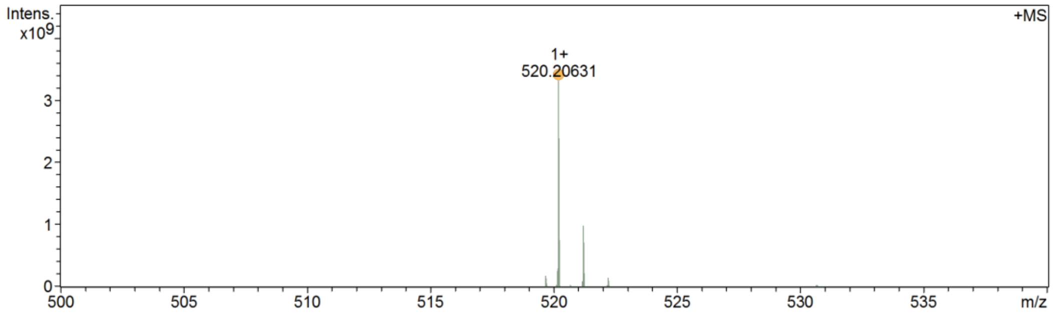

Meas. m/z	#	Ion Formula	Score	m/z	err [ppm]
520.206306	1	C26H29F3N3O5	100.00	520.205382	-1.8

Figure S32: HRMS positive ion spectra of the target compound 8g

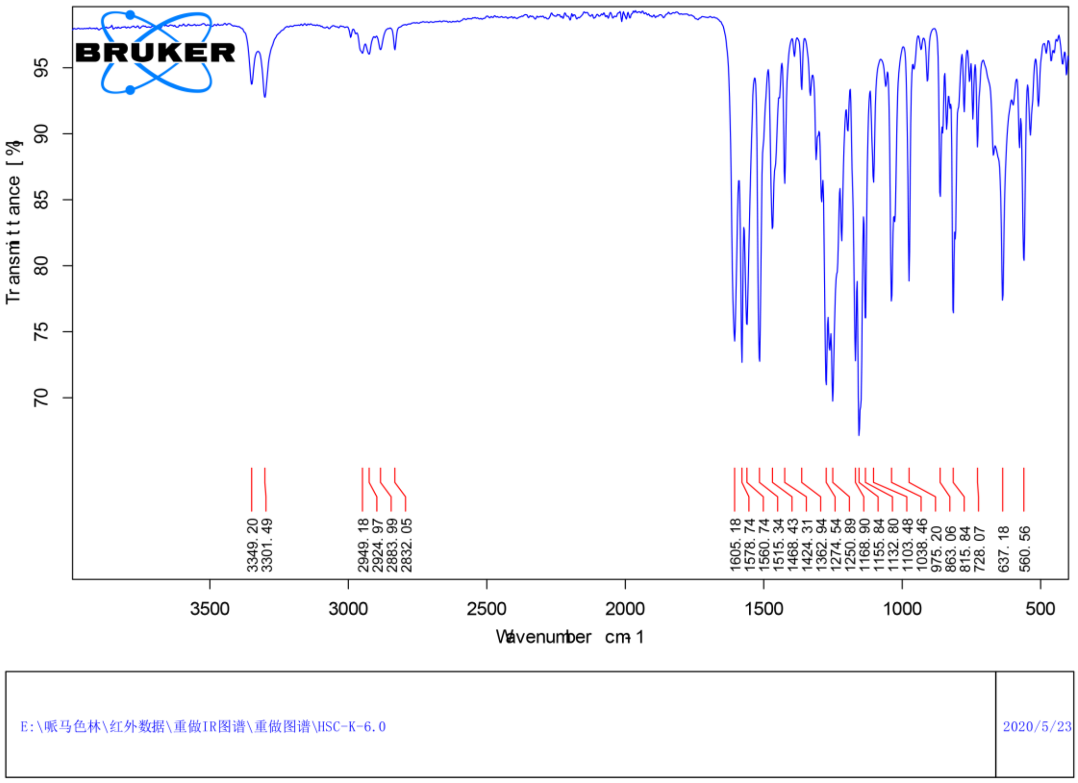

Figure S33: IR spectra of the target compound 8g

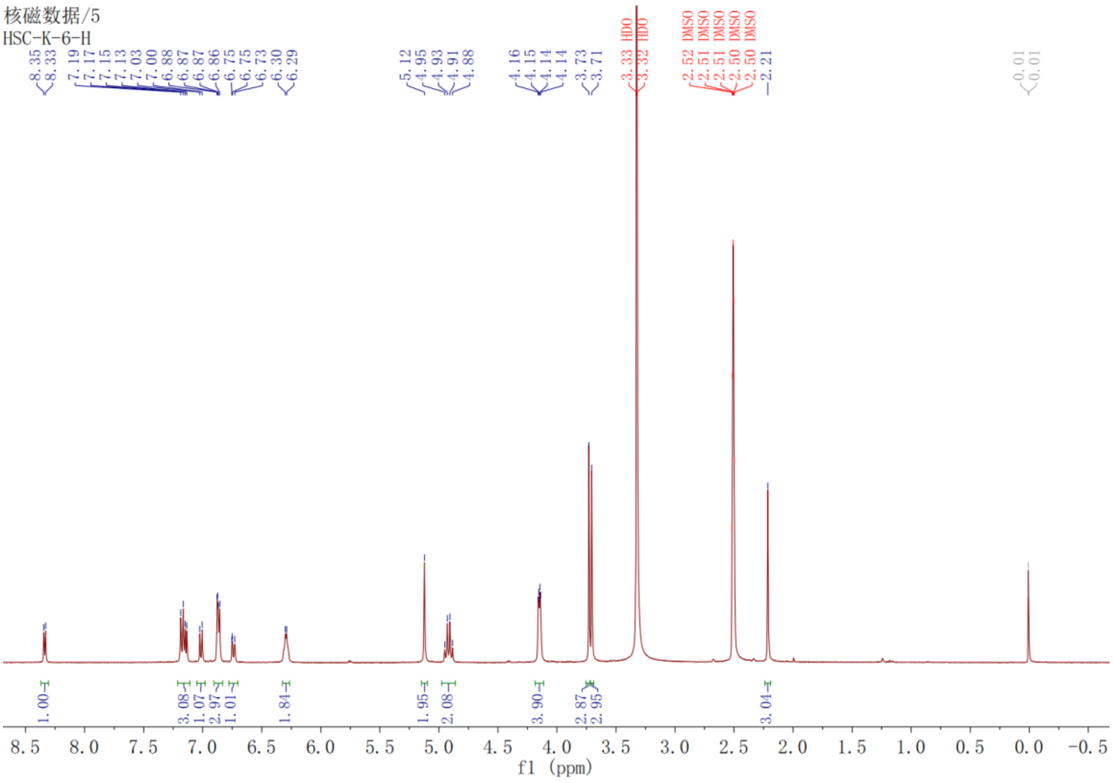

Figure S34: ^{1}H-NMR spectra of the target compound 8g

molecules

MDPI

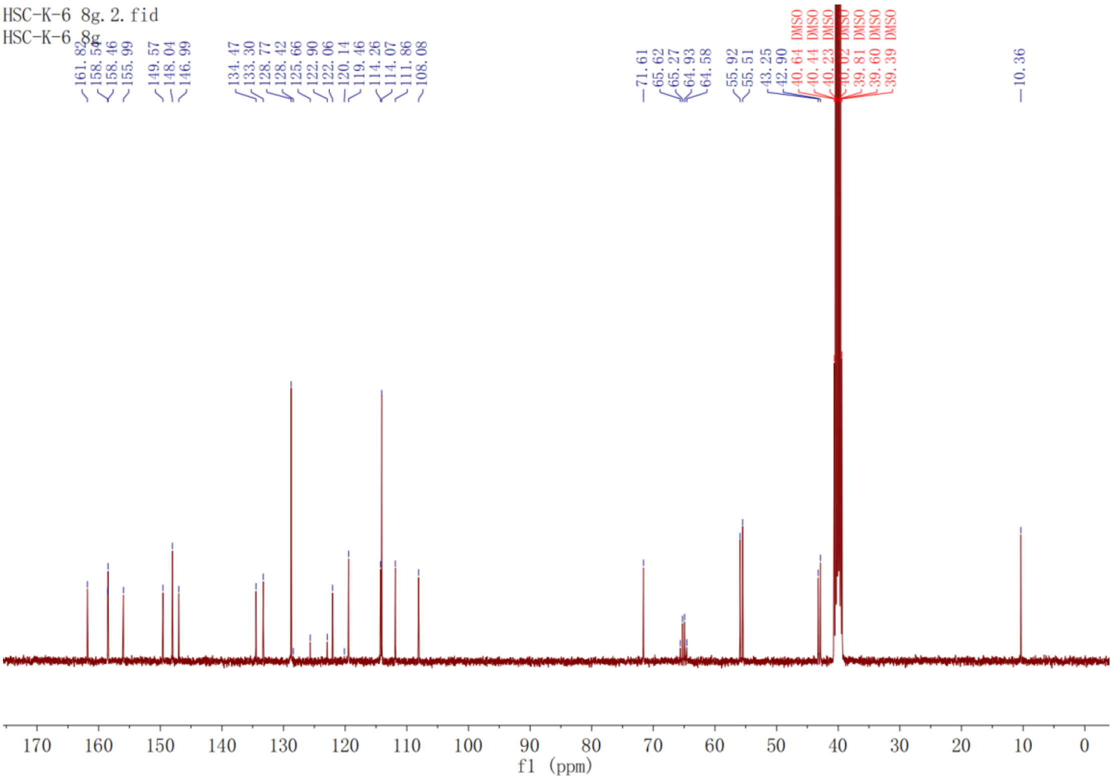

Figure S35: ^{13}C-NMR spectra of the target compound 8g

molecules

MDPI

Mass Spectrum SmartFormula Report

Analysis Info

Analysis Name	D:\data\HSC-G-20 neg_000001.d	Acquisition Date	3/17/2021 4:11:35 PM
Method	100-1200_neg_20210312	Operator	
Sample Name		Instrument	solariX
Comment			

Acquisition Parameter

Acquisition Mode	Single MS	Acquired Scans	3	Calibration Date	Fri Mar 12 09:12:31 2021
Polarity	Negative	No. of Cell Fills	1	Data Acquisition Size	1048576
Broadband Low Mass	100.3 m/z	No. of Laser Shots	200	Data Processing Size (SI)	2097152
Broadband High Mass	1200.0 m/z	Laser Power	20.0 lp	Apodization	Full-Sine
Source Accumulation	0.000 sec	Laser Shot Frequency	0.001 sec		
Ion Accumulation Time	0.050 sec				

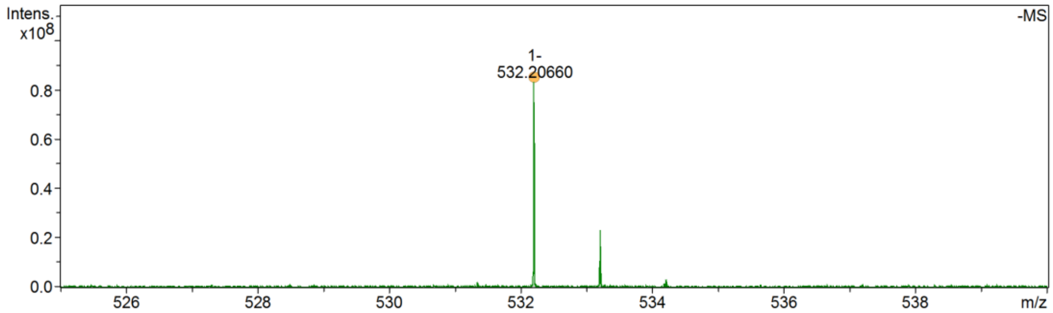

Meas. m/z	#	Ion Formula	Score	m/z	err [ppm]
532.206600	1	C27H29F3N3O5	100.00	532.206479	-0.2

Figure S36: HRMS Negative ion spectra of the target compound 8h

molecules MDPI

Mass Spectrum SmartFormula Report

Analysis Info

Analysis Name	D:\data\HSC-G-20 pos_000001.d	Acquisition Date	3/17/2021 4:14:47 PM
Method	100-1200_pos_20210312	Operator	
Sample Name		Instrument	solariX
Comment			

Acquisition Parameter

Acquisition Mode	Single MS	Acquired Scans	2	Calibration Date	Fri Mar 12 09:07:46 2021
Polarity	Positive	No. of Cell Fills	1	Data Acquisition Size	1048576
Broadband Low Mass	100.3 m/z	No. of Laser Shots	500	Data Processing Size (SI)	2097152
Broadband High Mass	1200.0 m/z	Laser Power	20.0 lp	Apodization	Full-Sine
Source Accumulation	0.000 sec	Laser Shot Frequency	0.001 sec		
Ion Accumulation Time	0.050 sec				

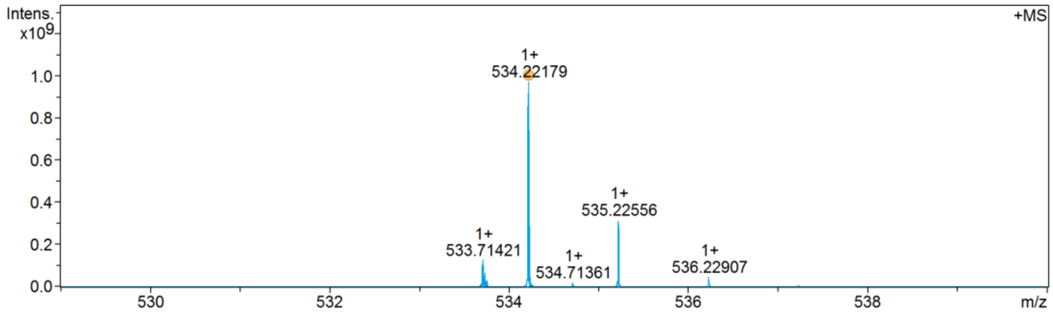

Meas. m/z	#	Ion Formula	Score	m/z	err [ppm]
534.221787	1	C27H31F3N3O5	100.00	534.221032	-1.4

Figure S37: HRMS positive ion spectra of the target compound 8h

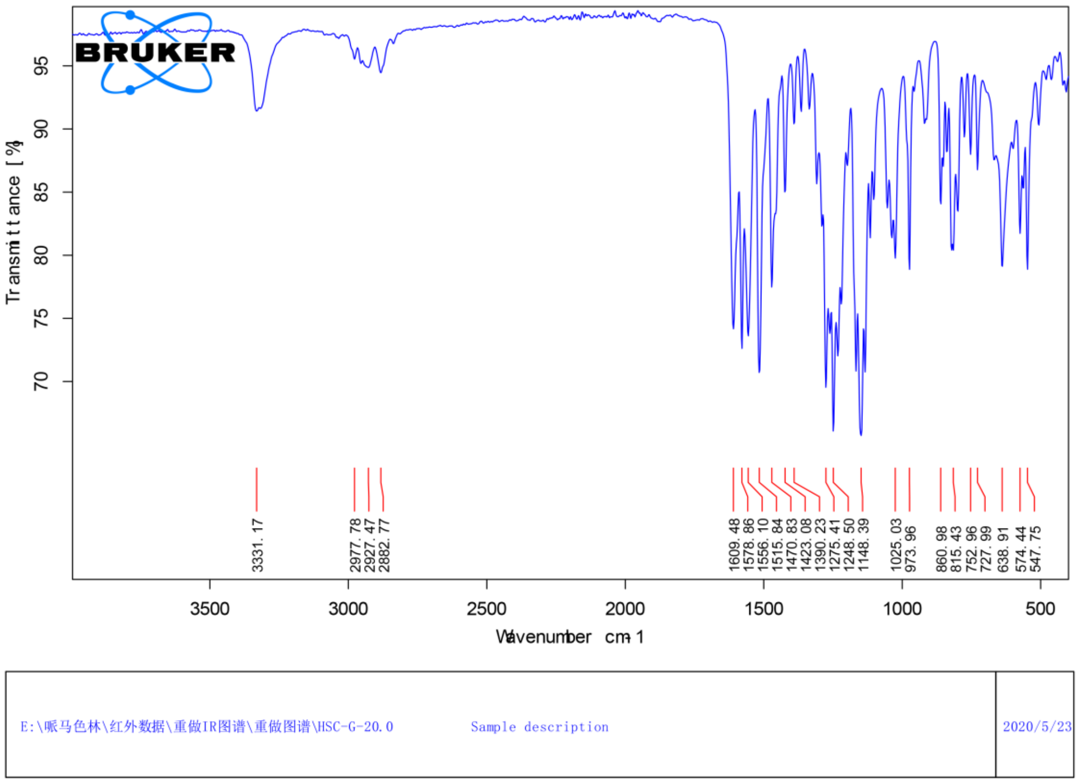

Figure S38: IR spectra of the target compound 8h

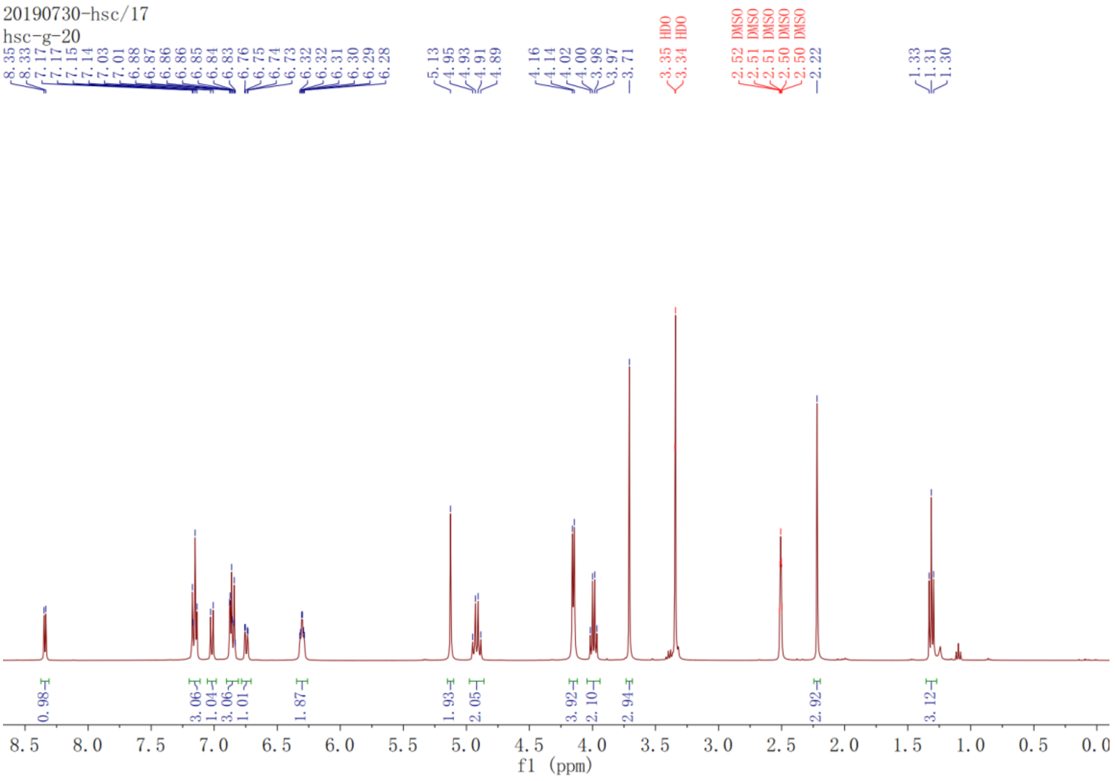

Figure S39: ^{1}H-NMR spectra of the target compound 8h

molecules

MDPI

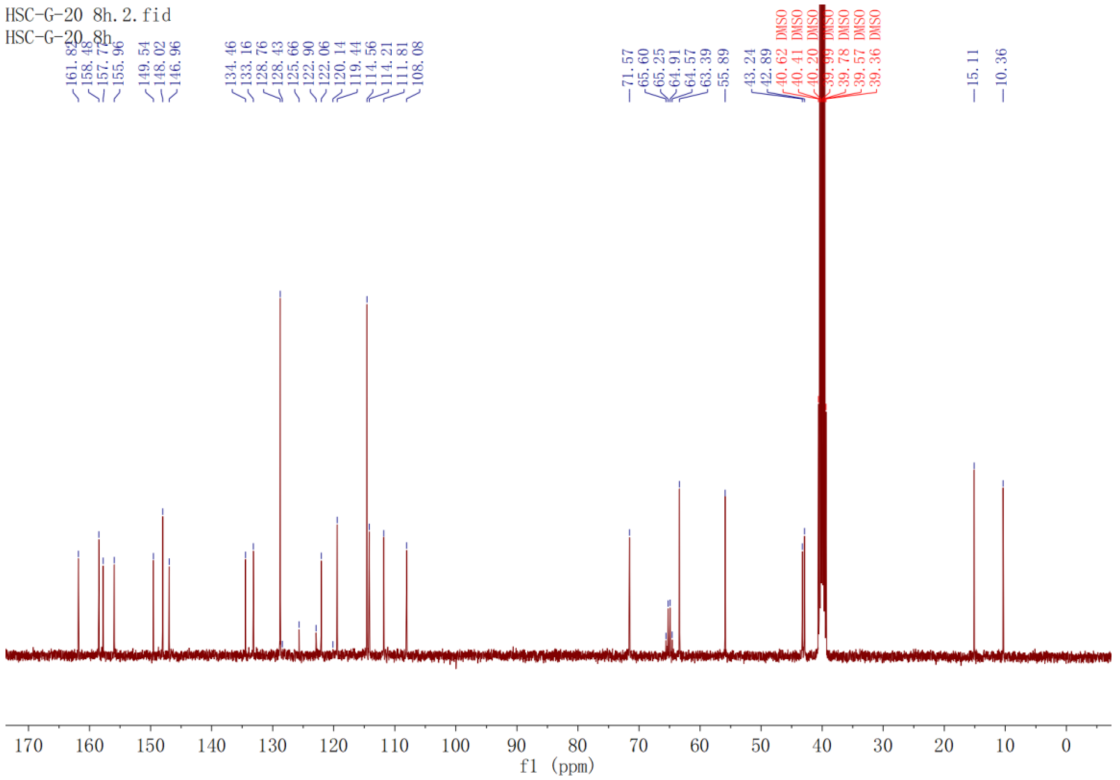

Figure S40: ^{13}C-NMR spectra of the target compound 8h

Mass Spectrum SmartFormula Report

Analysis Info

Analysis Name	D:\data\HSC-G-23 neg_000002.d	Acquisition Date	3/17/2021 5:45:46 PM
Method	100-1200_neg_20210312	Operator	
Sample Name		Instrument	solariX
Comment			

Acquisition Parameter

Acquisition Mode	Single MS	Acquired Scans	2	Calibration Date	Fri Mar 12 09:12:31 2021
Polarity	Negative	No. of Cell Fills	1	Data Acquisition Size	1048576
Broadband Low Mass	100.3 m/z	No. of Laser Shots	200	Data Processing Size (SI)	2097152
Broadband High Mass	1200.0 m/z	Laser Power	20.0 lp	Apodization	Full-Sine
Source Accumulation	0.000 sec	Laser Shot Frequency	0.001 sec		
Ion Accumulation Time	0.050 sec				

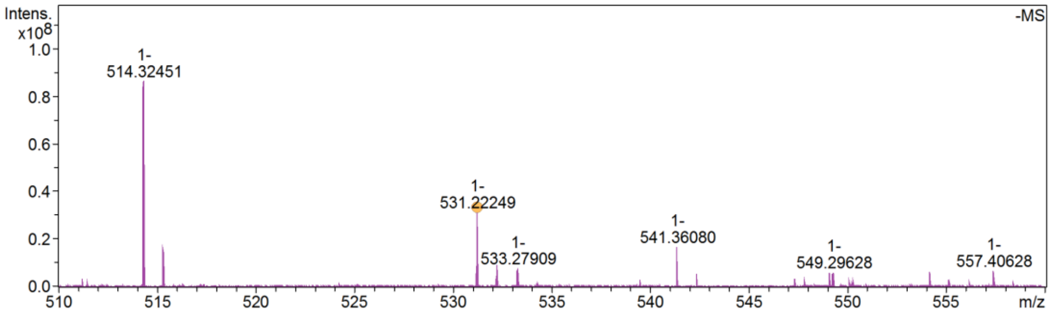

Meas. m/z	#	Ion Formula	Score	m/z	err [ppm]
531.222494	1	C27H30F3N4O4	100.00	531.222464	-0.1

Figure S41: HRMS negative ion spectra of the target compound 8i

molecules MDPI

Mass Spectrum SmartFormula Report

Analysis Info

Analysis Name	D:\data\HSC-G-23 pos_000003.d	Acquisition Date	3/17/2021 5:41:29 PM
Method	100-1200_pos_20210312	Operator	
Sample Name		Instrument	solariX
Comment			

Acquisition Parameter

Acquisition Mode	Single MS	Acquired Scans	2	Calibration Date	Fri Mar 12 09:07:46 2021
Polarity	Positive	No. of Cell Fills	1	Data Acquisition Size	1048576
Broadband Low Mass	100.3 m/z	No. of Laser Shots	500	Data Processing Size (SI)	2097152
Broadband High Mass	1200.0 m/z	Laser Power	20.0 lp	Apodization	Full-Sine
Source Accumulation	0.000 sec	Laser Shot Frequency	0.001 sec		
Ion Accumulation Time	0.050 sec				

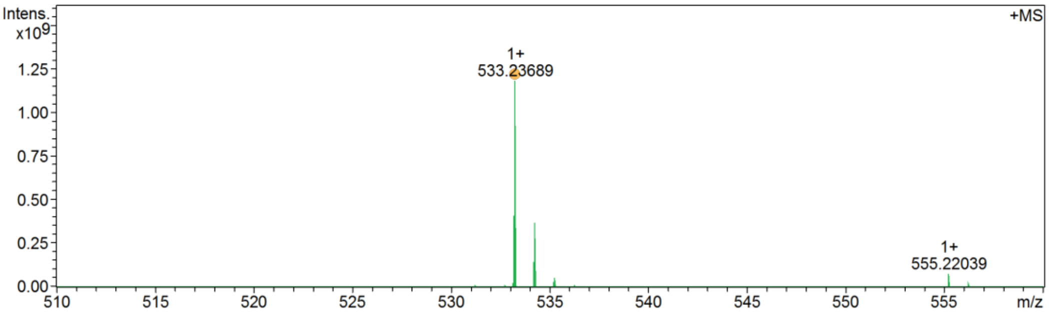

Meas. m/z	#	Ion Formula	Score	m/z	err [ppm]
533.236891	1	C27H32F3N4O4	100.00	533.237017	0.2

Figure S42: HRMS positive ion spectra of the target compound 8i

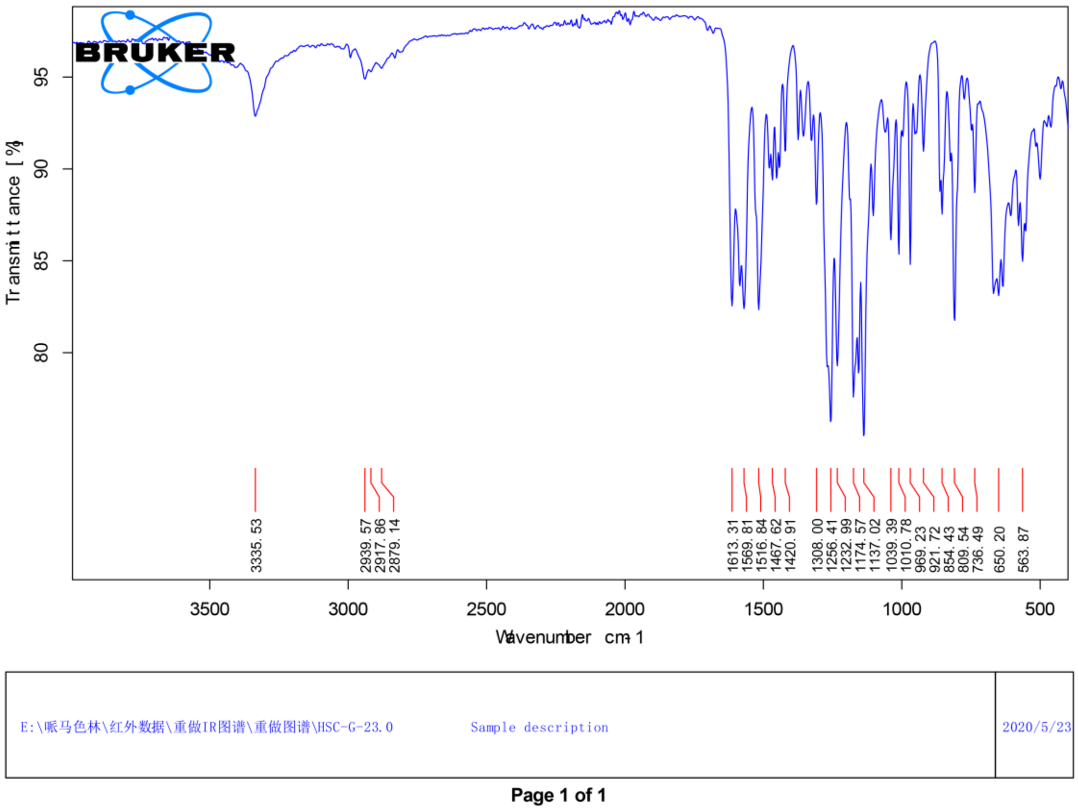

Figure S43: IR spectra of the target compound 8i

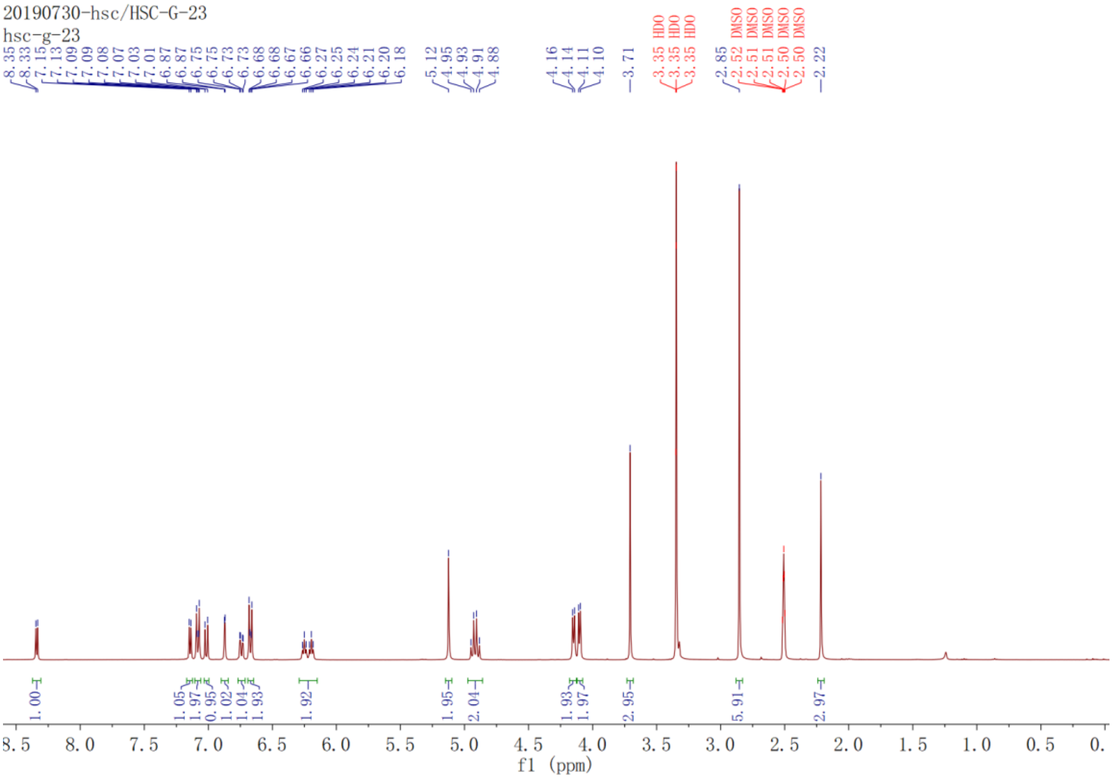

Figure S44: ^{1}H-NMR spectra of the target compound 8i

molecules MDPI

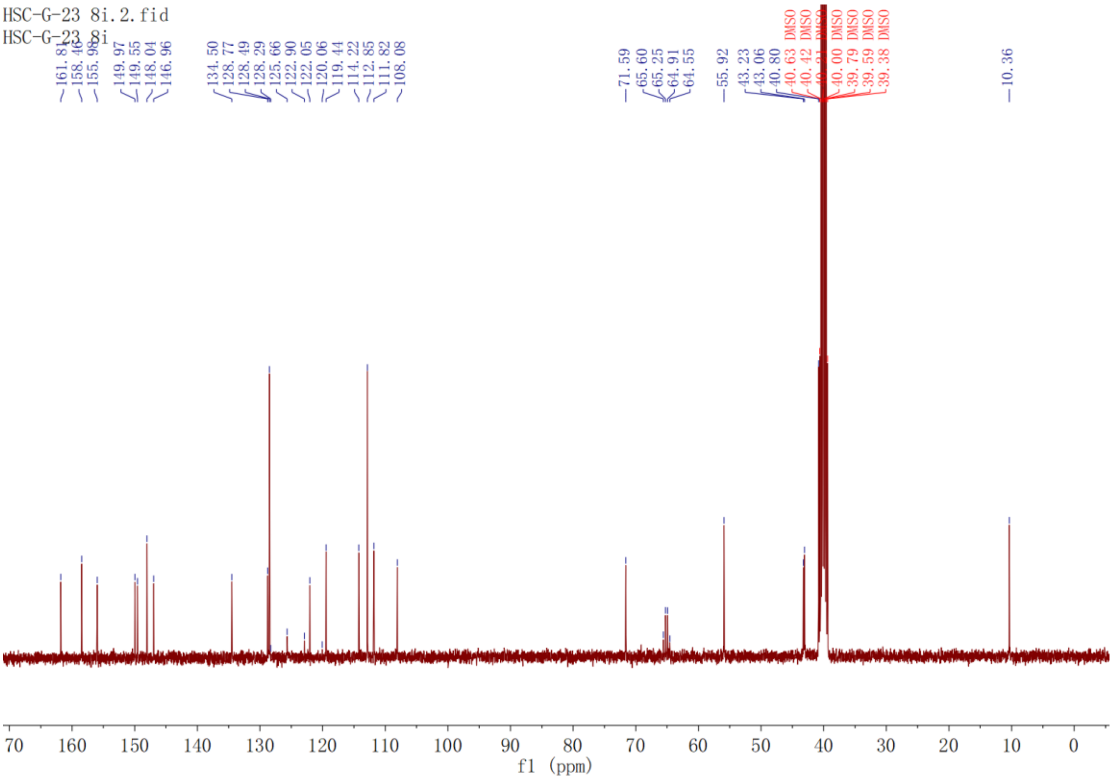

Figure S45: ^{13}C-NMR spectra of the target compound 8i

molecules MDPI

Mass Spectrum SmartFormula Report

Analysis Info

Analysis Name	D:\data\HSC-H-6 neg_000001.d	Acquisition Date	3/17/2021 3:38:30 PM
Method	100-1200_neg_20210312	Operator	
Sample Name		Instrument	solariX
Comment			

Acquisition Parameter

Acquisition Mode	Single MS	Acquired Scans	3	Calibration Date	Fri Mar 12 09:12:31 2021
Polarity	Negative	No. of Cell Fills	1	Data Acquisition Size	1048576
Broadband Low Mass	100.3 m/z	No. of Laser Shots	200	Data Processing Size (SI)	2097152
Broadband High Mass	1200.0 m/z	Laser Power	20.0 lp	Apodization	Full-Sine
Source Accumulation	0.000 sec	Laser Shot Frequency	0.001 sec		
Ion Accumulation Time	0.050 sec				

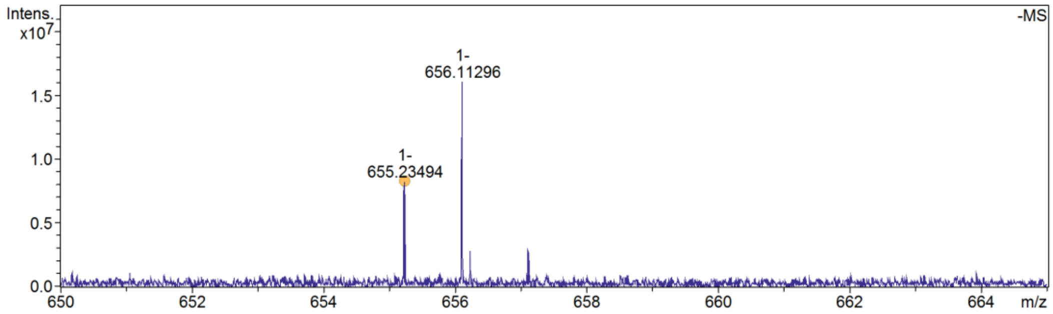

Meas. m/z	#	Ion Formula	Score	m/z	err [ppm]
655.234937	1	C31H33F6N4O5	100.00	655.236063	1.7

Figure S46: HRMS negative ion spectra of the target compound 9a

molecules MDPI

Mass Spectrum SmartFormula Report

Analysis Info

Analysis Name	D:\data\HSC-H-6 pos_000003.d	Acquisition Date	3/17/2021 3:35:46 PM
Method	100-1200_pos_20210312	Operator	
Sample Name		Instrument	solariX
Comment			

Acquisition Parameter

Acquisition Mode	Single MS	Acquired Scans	2	Calibration Date	Fri Mar 12 09:07:46 2021
Polarity	Positive	No. of Cell Fills	1	Data Acquisition Size	1048576
Broadband Low Mass	100.3 m/z	No. of Laser Shots	500	Data Processing Size (SI)	2097152
Broadband High Mass	1200.0 m/z	Laser Power	20.0 lp	Apodization	Full-Sine
Source Accumulation	0.000 sec	Laser Shot Frequency	0.001 sec		
Ion Accumulation Time	0.050 sec				

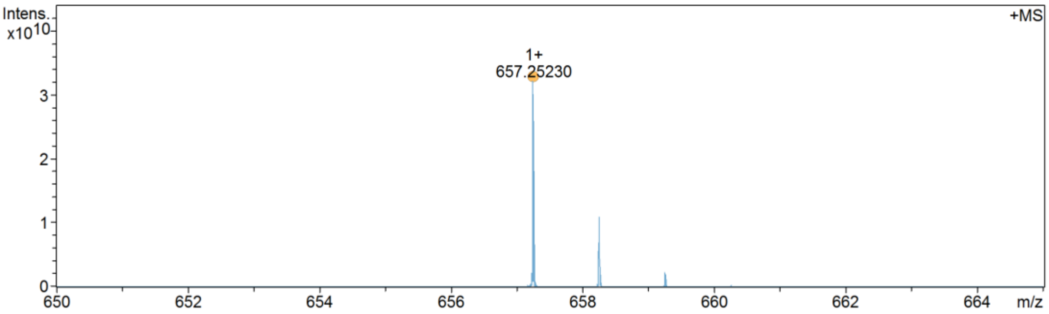

Meas. m/z	#	Ion Formula	Score	m/z	err [ppm]
657.252304	1	C31H35F6N4O5	100.00	657.250616	-2.6

Figure S47: HRMS positive ion spectra of the target compound 9a

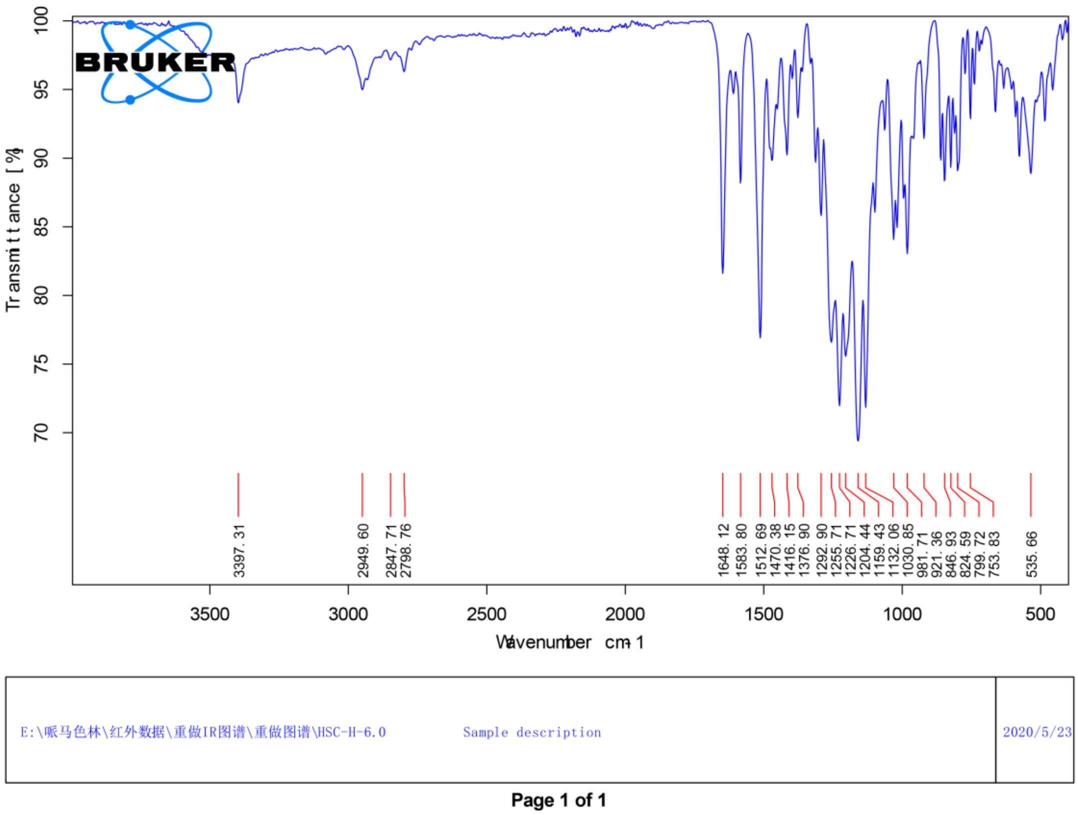

Figure S48: IR spectra of the target compound 9a

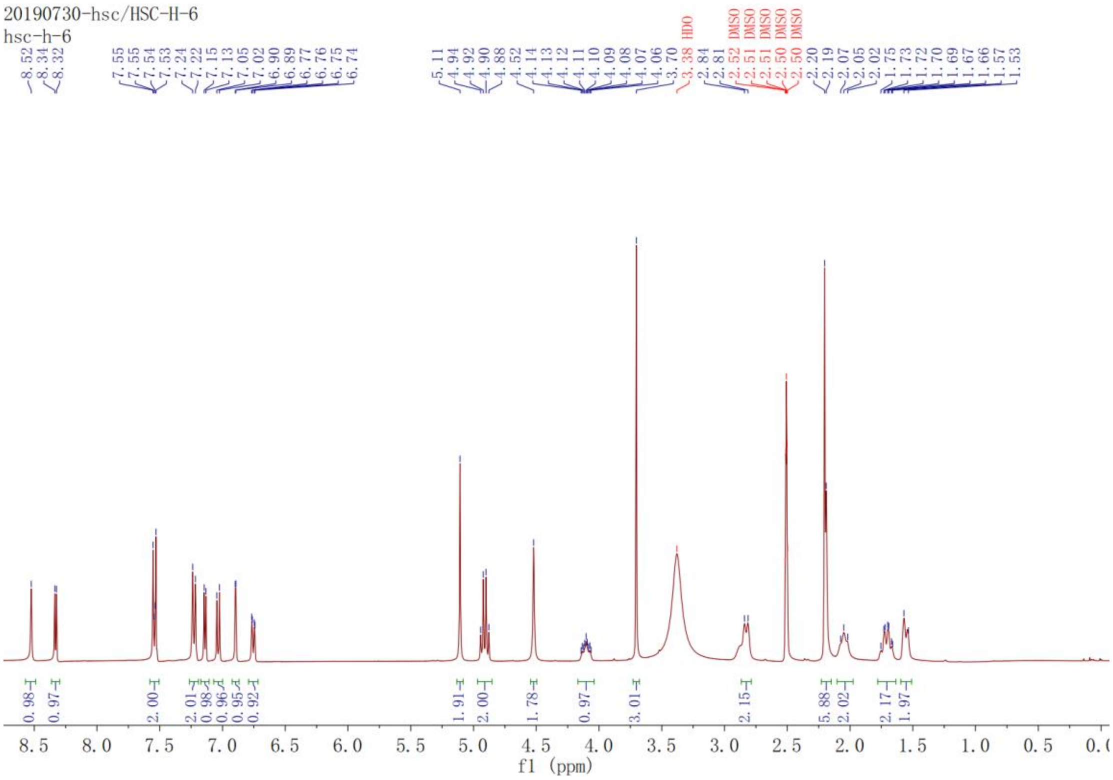

Figure S49: ^{1}H-NMR spectra of the target compound 9a

molecules

MDPI

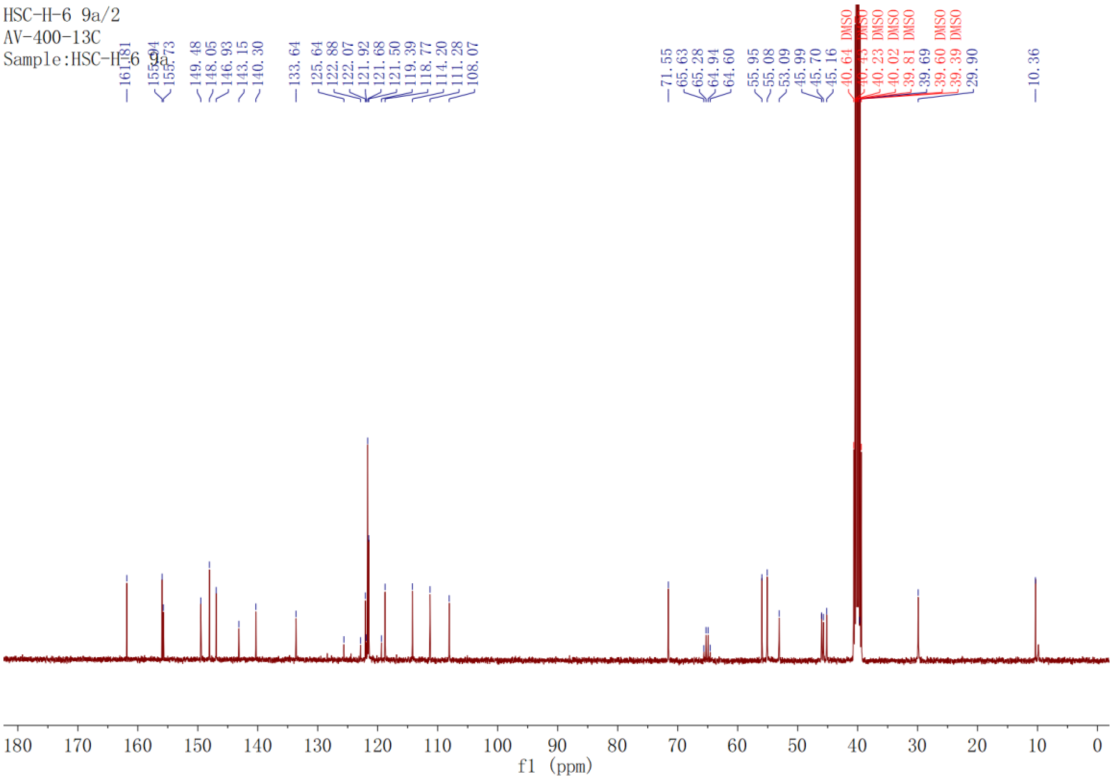

Figure S50: ^{13}C-NMR spectra of the target compound 9a

molecules MDPI

Mass Spectrum SmartFormula Report

Analysis Info

Analysis Name	D:\data\20210317\HSC-H-23 neg_000001.d	Acquisition Date	3/17/2021 6:53:13 PM
Method	100-1200_neg_20210312	Operator	
Sample Name		Instrument	solariX
Comment			

Acquisition Parameter

Acquisition Mode	Single MS	Acquired Scans	4	Calibration Date	Fri Mar 12 09:12:31 2021
Polarity	Negative	No. of Cell Fills	1	Data Acquisition Size	1048576
Broadband Low Mass	100.3 m/z	No. of Laser Shots	200	Data Processing Size (SI)	2097152
Broadband High Mass	1200.0 m/z	Laser Power	20.0 lp	Apodization	Full-Sine
Source Accumulation	0.000 sec	Laser Shot Frequency	0.001 sec		
Ion Accumulation Time	0.050 sec				

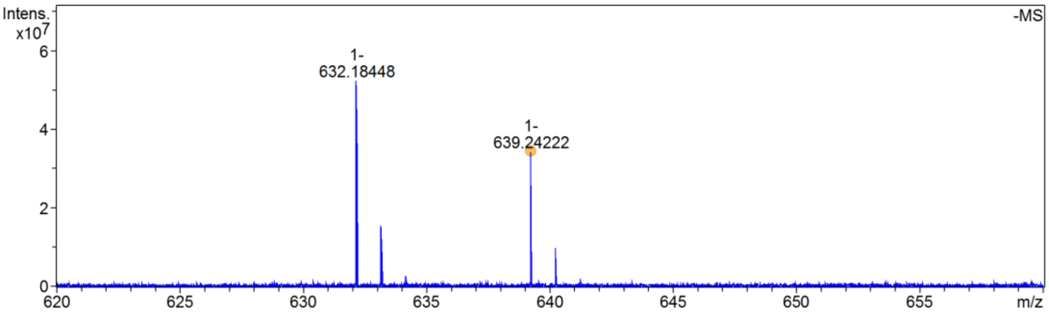

Meas. m/z	#	Ion Formula	Score	m/z	err [ppm]
639.242219	1	C31H33F6N4O4	100.00	639.241148	-1.7

Figure S51: HRMS negative ion spectra of the target compound 9b

molecules MDPI

Mass Spectrum SmartFormula Report

Analysis Info

Analysis Name	D:\data\20210317\HSC-H-23 pos_000006.d	Acquisition Date	3/17/2021 7:06:19 PM
Method	100-1200_pos_20210312	Operator	
Sample Name		Instrument	solariX
Comment			

Acquisition Parameter

Acquisition Mode	Single MS	Acquired Scans	2	Calibration Date	Fri Mar 12 09:07:46 2021
Polarity	Positive	No. of Cell Fills	1	Data Acquisition Size	1048576
Broadband Low Mass	100.3 m/z	No. of Laser Shots	500	Data Processing Size (SI)	2097152
Broadband High Mass	1200.0 m/z	Laser Power	20.0 lp	Apodization	Full-Sine
Source Accumulation	0.000 sec	Laser Shot Frequency	0.001 sec		
Ion Accumulation Time	0.050 sec				

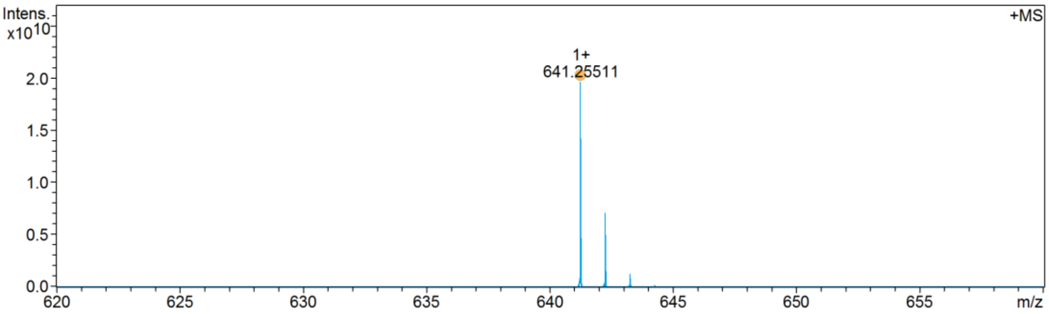

Meas. m/z	#	Ion Formula	Score	m/z	err [ppm]
641.255106	1	C31H35F6N4O4	100.00	641.255701	0.9

Figure S52: HRMS positive ion spectra of the target compound 9b

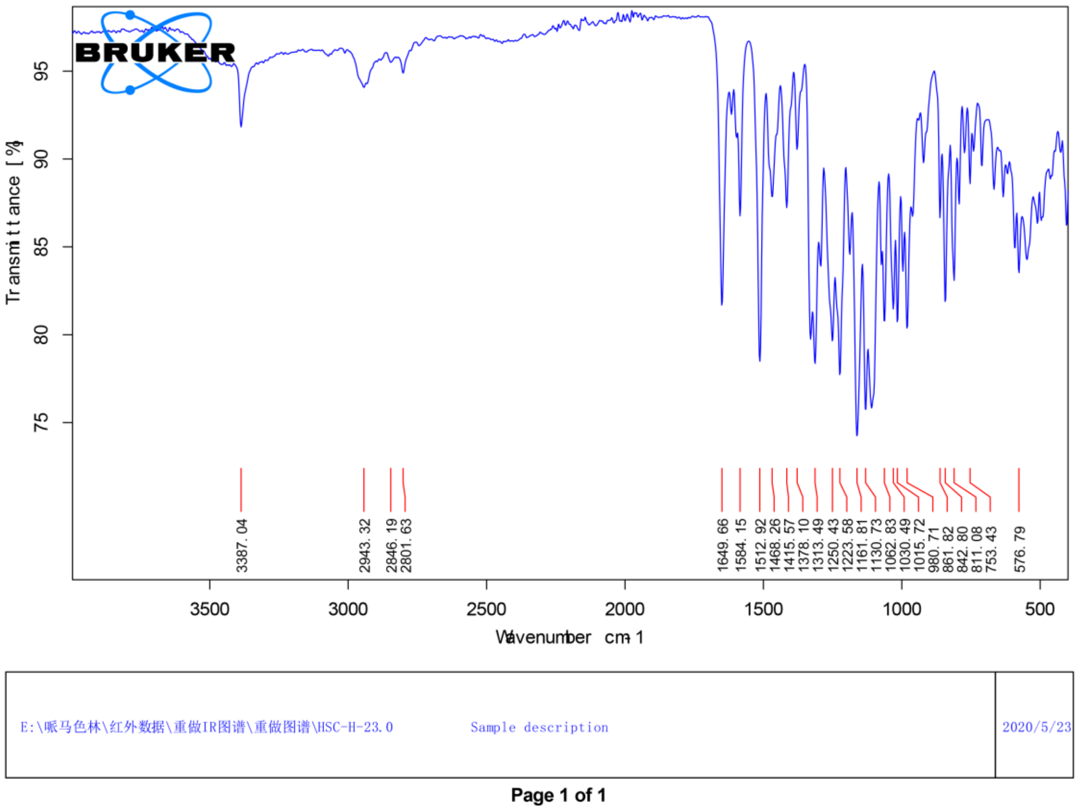

Figure S53: IR spectra of the target compound 9b

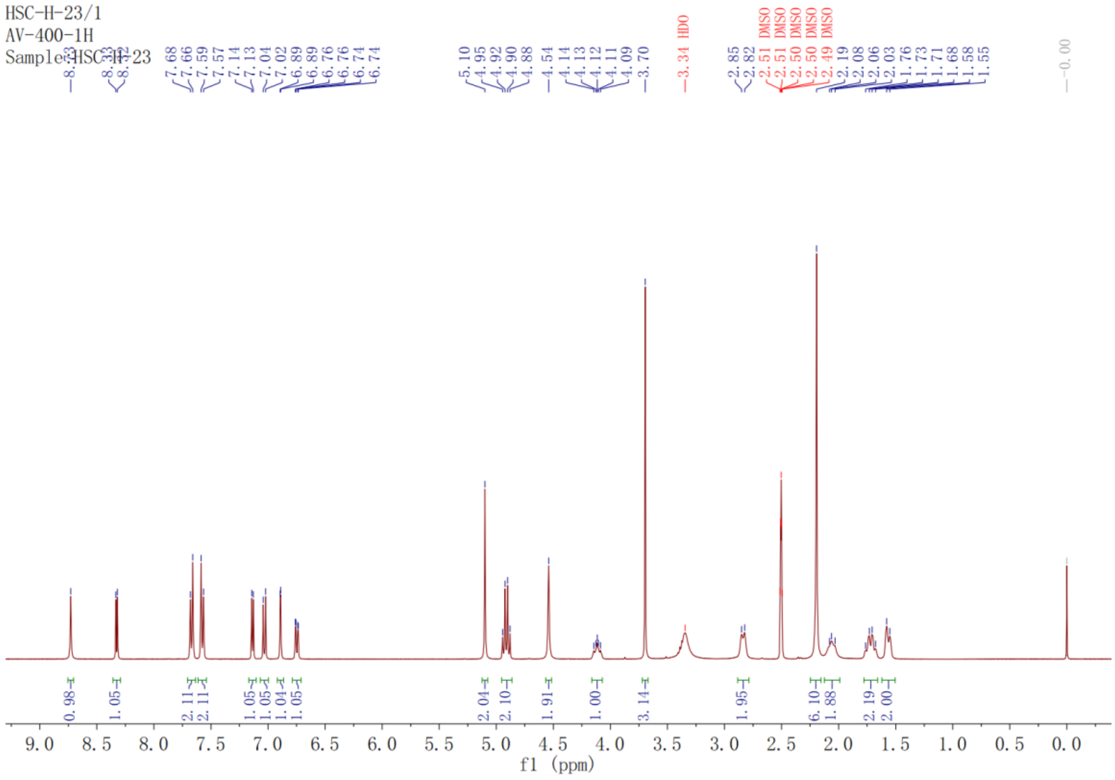

Figure S54: ^{1}H-NMR spectra of the target compound 9b

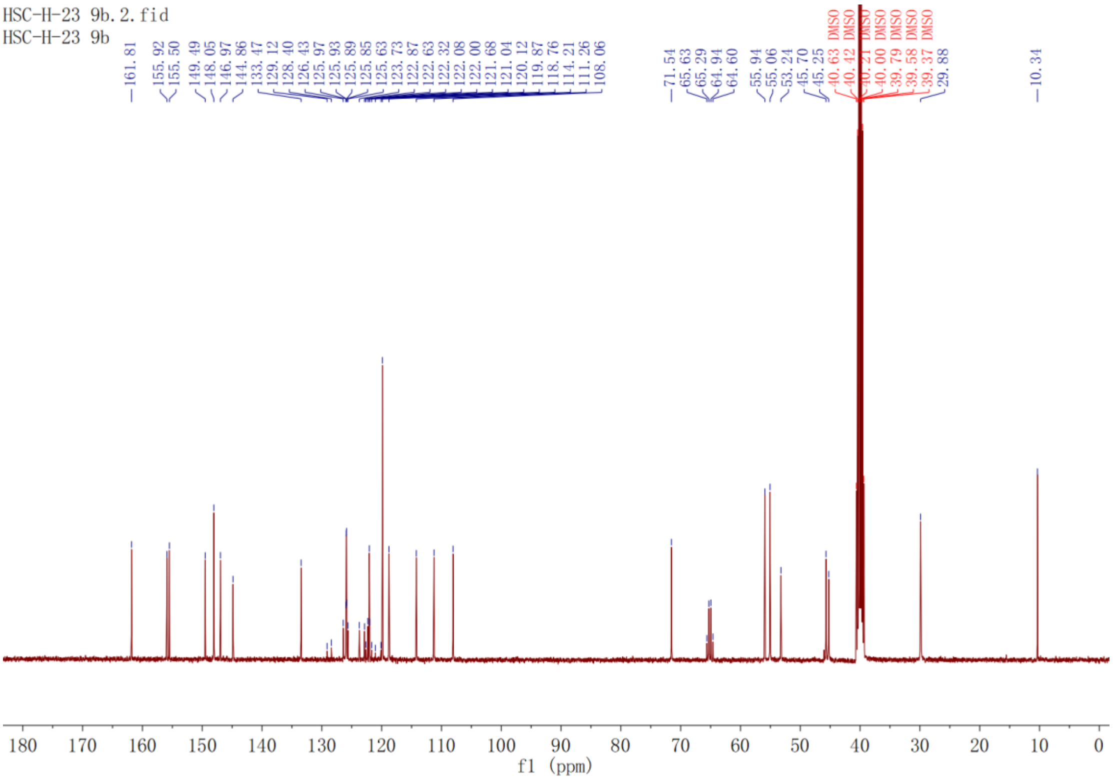

Figure S55: ^{13}C-NMR spectra of the target compound 9b

molecules

MDPI

Mass Spectrum SmartFormula Report

Analysis Info

Analysis Name	D:\data\HSC-H-26 neg_000001.d	Acquisition Date	3/17/2021 5:25:02 PM
Method	100-1200_neg_20210312	Operator	
Sample Name		Instrument	solariX
Comment			

Acquisition Parameter

Acquisition Mode	Single MS	Acquired Scans	6	Calibration Date	Fri Mar 12 09:12:31 2021
Polarity	Negative	No. of Cell Fills	1	Data Acquisition Size	1048576
Broadband Low Mass	100.3 m/z	No. of Laser Shots	200	Data Processing Size (SI)	2097152
Broadband High Mass	1200.0 m/z	Laser Power	20.0 lp	Apodization	Full-Sine
Source Accumulation	0.000 sec	Laser Shot Frequency	0.001 sec		
Ion Accumulation Time	0.050 sec				

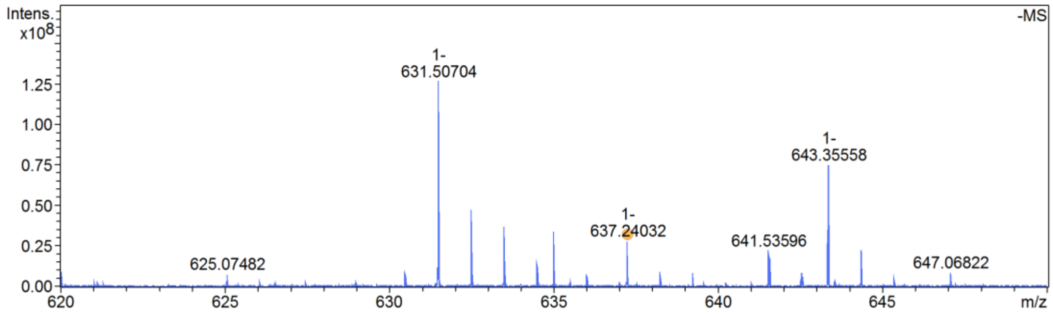

Meas. m/z	#	Ion Formula	Score	m/z	err [ppm]
637.240324	1	C31H37ClF3N4O5	100.00	637.241006	1.1

Figure S56: HRMS negative ion spectra of the target compound 9c

molecules MDPI

Mass Spectrum SmartFormula Report

Analysis Info

Analysis Name	D:\data\HSC-H-26 pos_000003.d	Acquisition Date	3/17/2021 5:33:52 PM
Method	100-1200_pos_20210312	Operator	
Sample Name		Instrument	solariX
Comment			

Acquisition Parameter

Acquisition Mode	Single MS	Acquired Scans	2	Calibration Date	Fri Mar 12 09:07:46 2021
Polarity	Positive	No. of Cell Fills	1	Data Acquisition Size	1048576
Broadband Low Mass	100.3 m/z	No. of Laser Shots	500	Data Processing Size (SI)	2097152
Broadband High Mass	1200.0 m/z	Laser Power	20.0 lp	Apodization	Full-Sine
Source Accumulation	0.000 sec	Laser Shot Frequency	0.001 sec		
Ion Accumulation Time	0.050 sec				

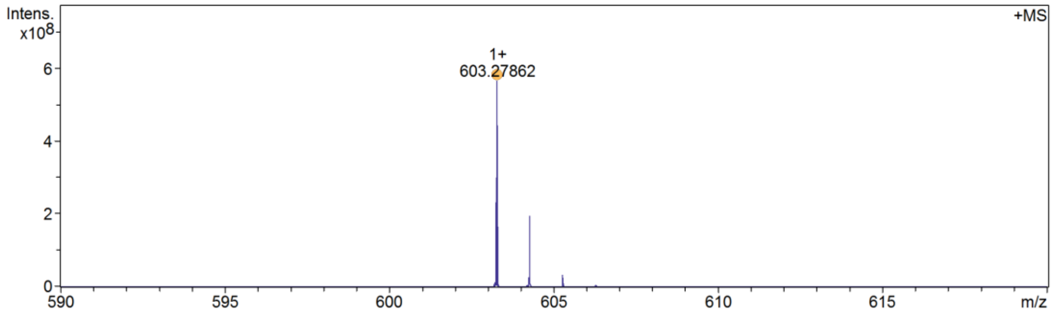

Meas. m/z	#	Ion Formula	Score	m/z	err [ppm]
603.278617	1	C31H38F3N4O5	100.00	603.278881	0.4

Figure S57: HRMS positive ion spectra of the target compound 9c

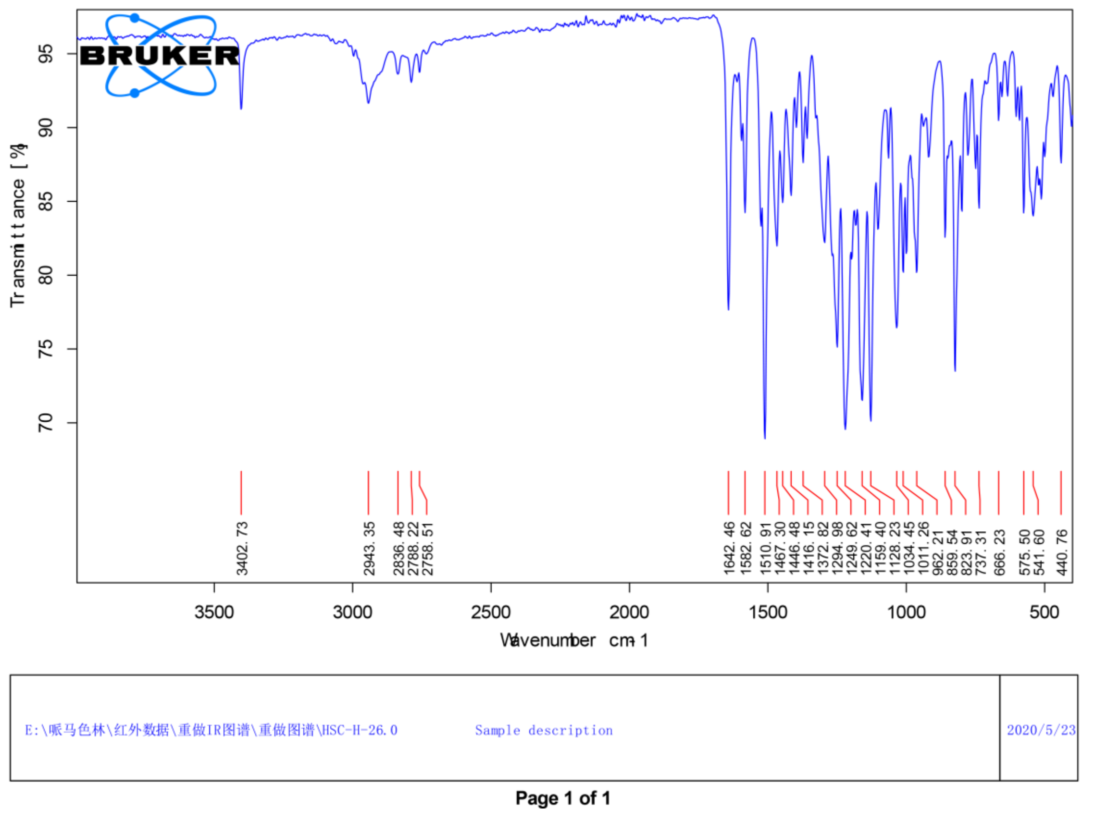

Figure S58: IR spectra of the target compound 9c

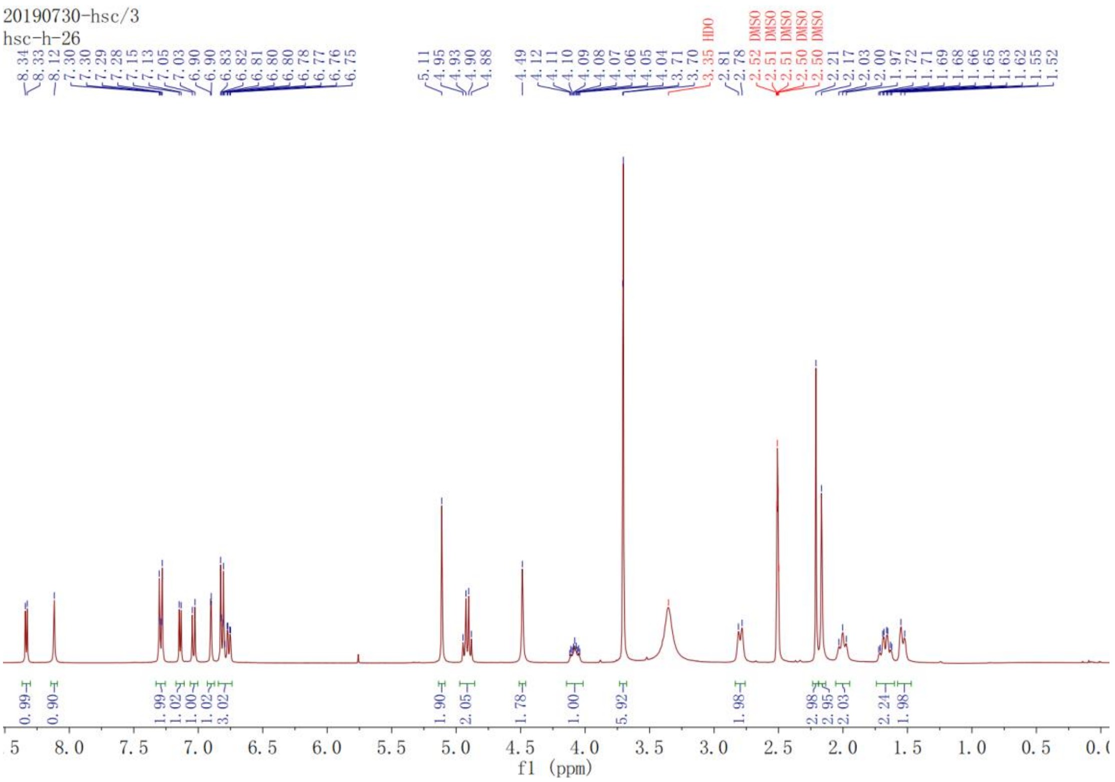

Figure S59: ^{1}H-NMR spectra of the target compound 9c

molecules MDPI

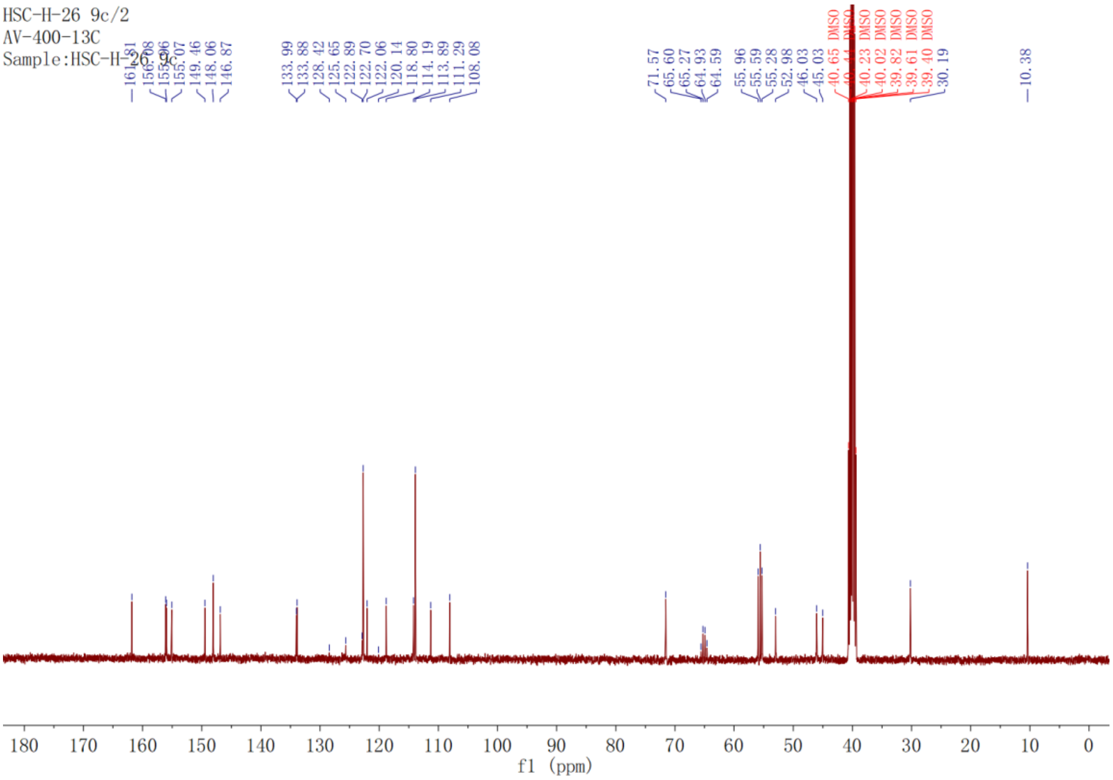

Figure S60: ^{13}C-NMR spectra of the target compound 9c

molecules MDPI

Mass Spectrum SmartFormula Report

Analysis Info

Analysis Name	D:\data\HSC-H-27 neg_000002.d	Acquisition Date	3/17/2021 5:04:11 PM
Method	100-1200_neg_20210312	Operator	
Sample Name		Instrument	solariX
Comment			

Acquisition Parameter

Acquisition Mode	Single MS	Acquired Scans	2	Calibration Date	Fri Mar 12 09:12:31 2021
Polarity	Negative	No. of Cell Fills	1	Data Acquisition Size	1048576
Broadband Low Mass	100.3 m/z	No. of Laser Shots	200	Data Processing Size (SI)	2097152
Broadband High Mass	1200.0 m/z	Laser Power	20.0 lp	Apodization	Full-Sine
Source Accumulation	0.000 sec	Laser Shot Frequency	0.001 sec		
Ion Accumulation Time	0.050 sec				

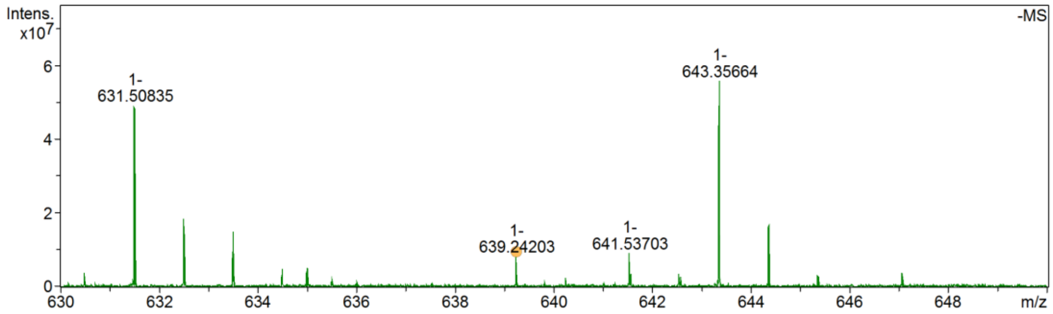

Meas. m/z	#	Ion Formula	Score	m/z	err [ppm]
639.242027	1	C31H33F6N4O4	100.00	639.241148	-1.4

Figure S61: HRMS negative ion spectra of the target compound 9d

Mass Spectrum SmartFormula Report

Analysis Info

Analysis Name	D:\data\HSC-H-27 pos_000017.d	Acquisition Date	3/17/2021 5:10:42 PM
Method	100-1200_pos_20210312	Operator	
Sample Name		Instrument	solariX
Comment			

Acquisition Parameter

Acquisition Mode	Single MS	Acquired Scans	2	Calibration Date	Fri Mar 12 09:07:46 2021
Polarity	Positive	No. of Cell Fills	1	Data Acquisition Size	1048576
Broadband Low Mass	100.3 m/z	No. of Laser Shots	500	Data Processing Size (SI)	2097152
Broadband High Mass	1200.0 m/z	Laser Power	20.0 lp	Apodization	Full-Sine
Source Accumulation	0.000 sec	Laser Shot Frequency	0.001 sec		
Ion Accumulation Time	0.050 sec				

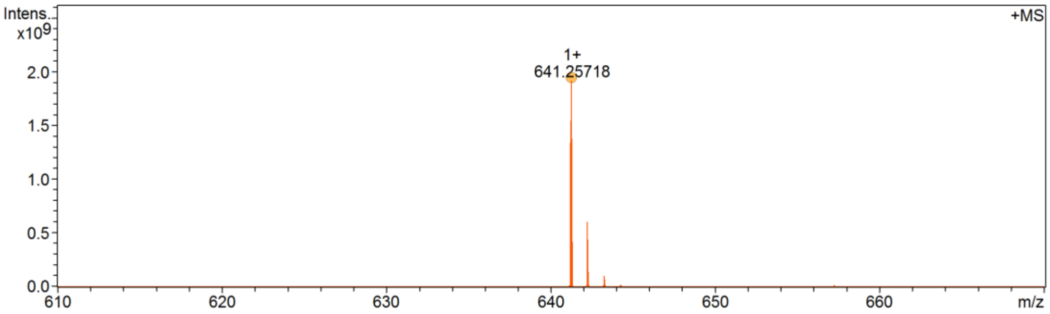

Meas. m/z	#	Ion Formula	Score	m/z	err [ppm]
641.257179	1	C31H35F6N4O4	100.00	641.255701	-2.3

Figure S62: HRMS positive ion spectra of the target compound 9d

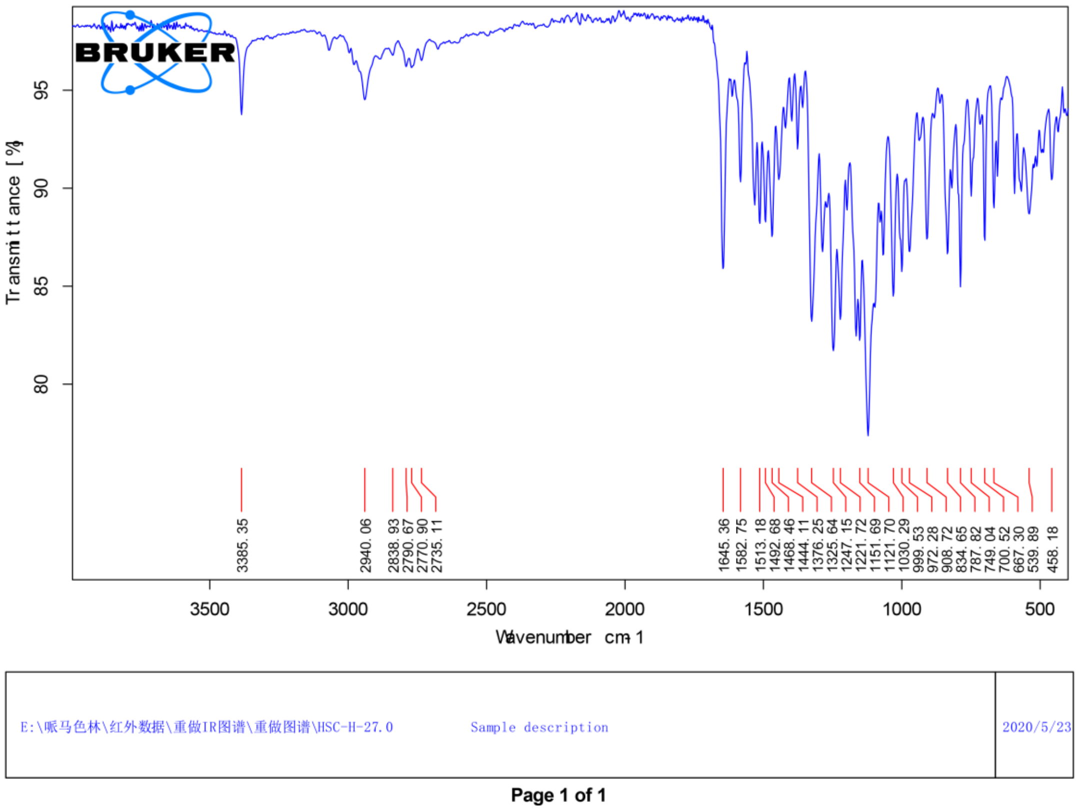

Figure S63: IR spectra of the target compound 9d

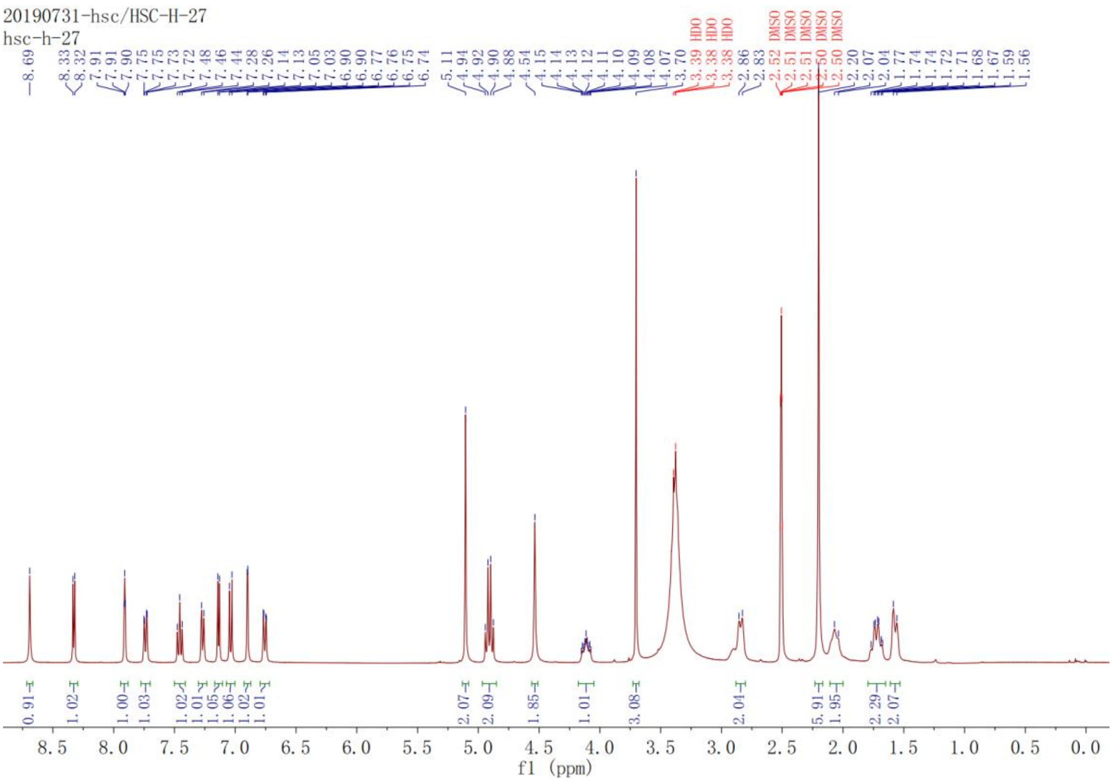

Figure S64: ^{1}H-NMR spectra of the target compound 9d

molecules

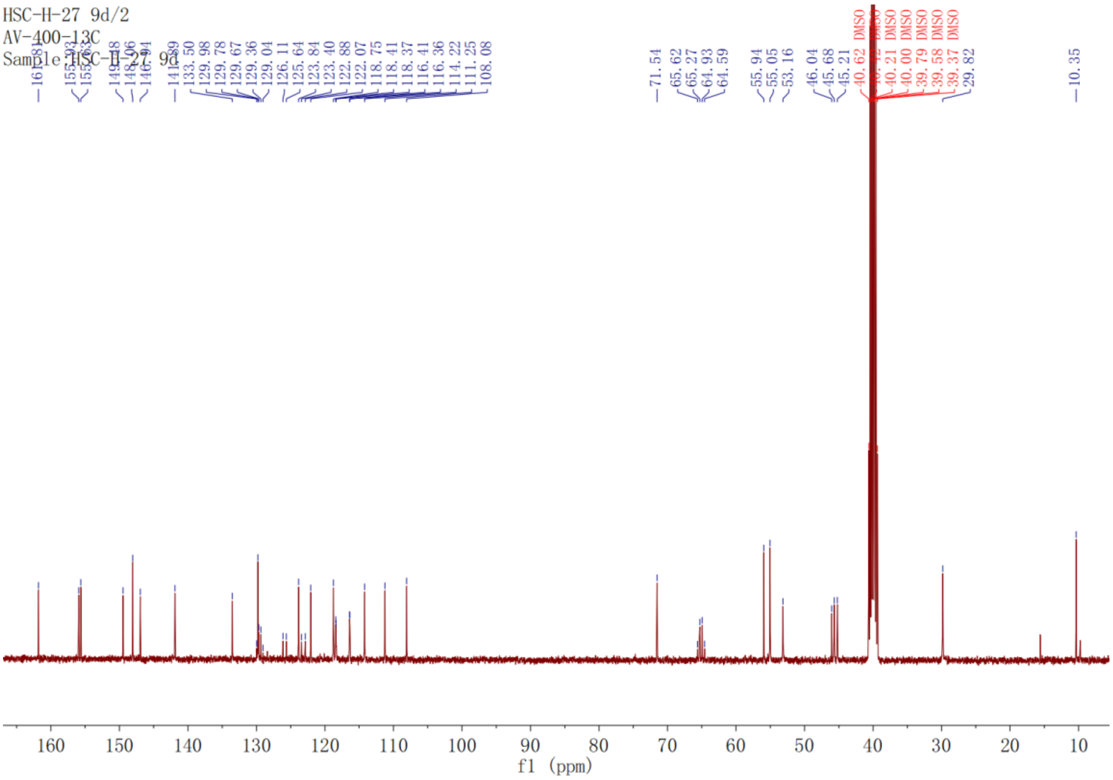

Figure S65: ^{13}C-NMR spectra of the target compound 9d

molecules MDPI

Mass Spectrum SmartFormula Report

Analysis Info

Analysis Name	D:\data\HSC-H-28 neg_000001.d	Acquisition Date	3/17/2021 5:53:37 PM
Method	100-1200_neg_20210312	Operator	
Sample Name		Instrument	solariX
Comment			

Acquisition Parameter

Acquisition Mode	Single MS	Acquired Scans	3	Calibration Date	Fri Mar 12 09:12:31 2021
Polarity	Negative	No. of Cell Fills	1	Data Acquisition Size	1048576
Broadband Low Mass	100.3 m/z	No. of Laser Shots	200	Data Processing Size (SI)	2097152
Broadband High Mass	1200.0 m/z	Laser Power	20.0 lp	Apodization	Full-Sine
Source Accumulation	0.000 sec	Laser Shot Frequency	0.001 sec		
Ion Accumulation Time	0.050 sec				

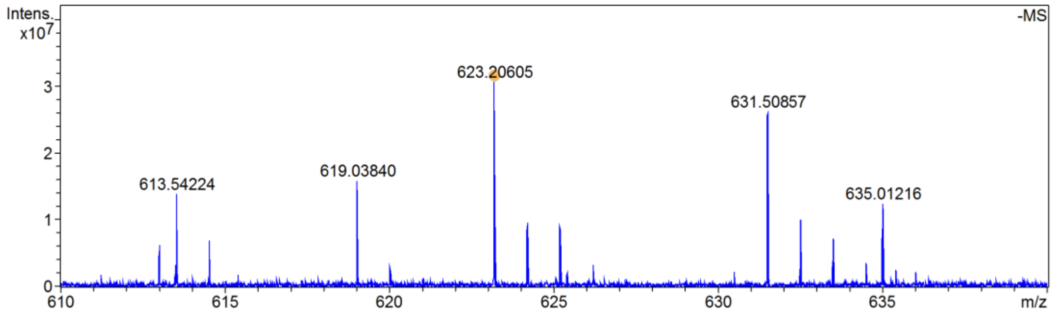

Meas. m/z	#	Ion Formula	Score	m/z	err [ppm]
623.206049	1	C30H32ClF4N4O4	100.00	623.205370	-1.1

Figure S66: HRMS negative spectra of the target compound 9e

Mass Spectrum SmartFormula Report

Analysis Info

Analysis Name	D:\data\HSC-H-28 pos_000006.d	Acquisition Date	3/17/2021 6:00:55 PM
Method	100-1200_pos_20210312	Operator	
Sample Name		Instrument	solariX
Comment			

Acquisition Parameter

Acquisition Mode	Single MS	Acquired Scans	2	Calibration Date	Fri Mar 12 09:07:46 2021
Polarity	Positive	No. of Cell Fills	1	Data Acquisition Size	1048576
Broadband Low Mass	100.3 m/z	No. of Laser Shots	500	Data Processing Size (SI)	2097152
Broadband High Mass	1200.0 m/z	Laser Power	20.0 lp	Apodization	Full-Sine
Source Accumulation	0.000 sec	Laser Shot Frequency	0.001 sec		
Ion Accumulation Time	0.050 sec				

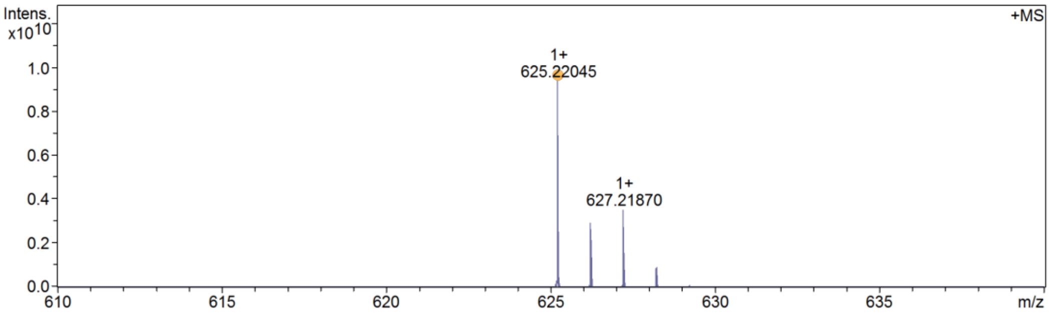

Meas. m/z	#	Ion Formula	Score	m/z	err [ppm]
625.220446	1	C30H34ClF4N4O4	100.00	625.219923	-0.8

Figure S67: HRMS positive ion spectra of the target compound 9e

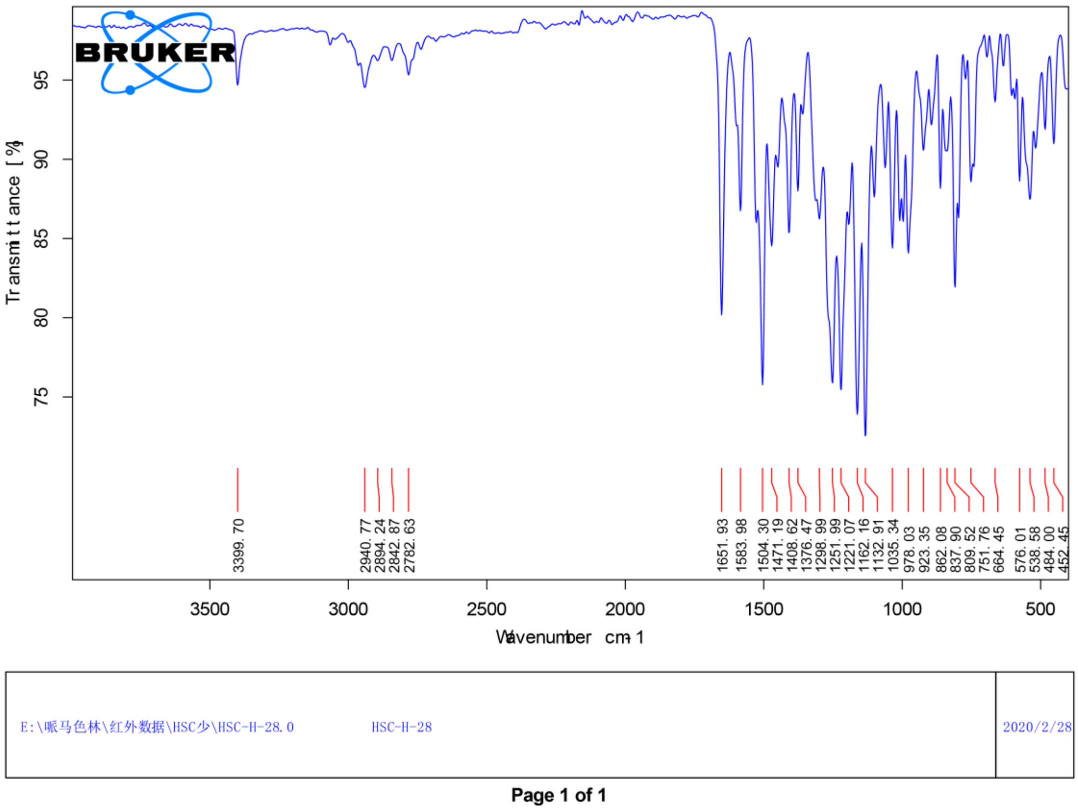

Figure S68: IR spectra of the target compound 9e

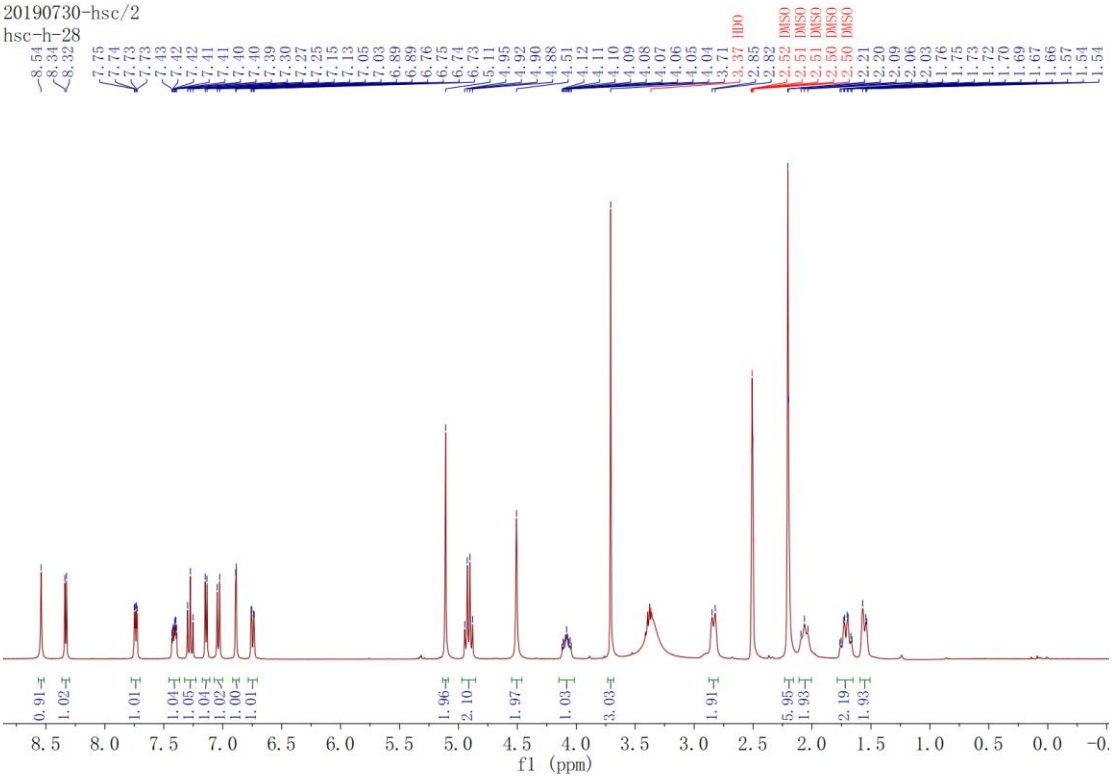

Figure S69: ^{1}H-NMR spectra of the target compound 9e

molecules

MDPI

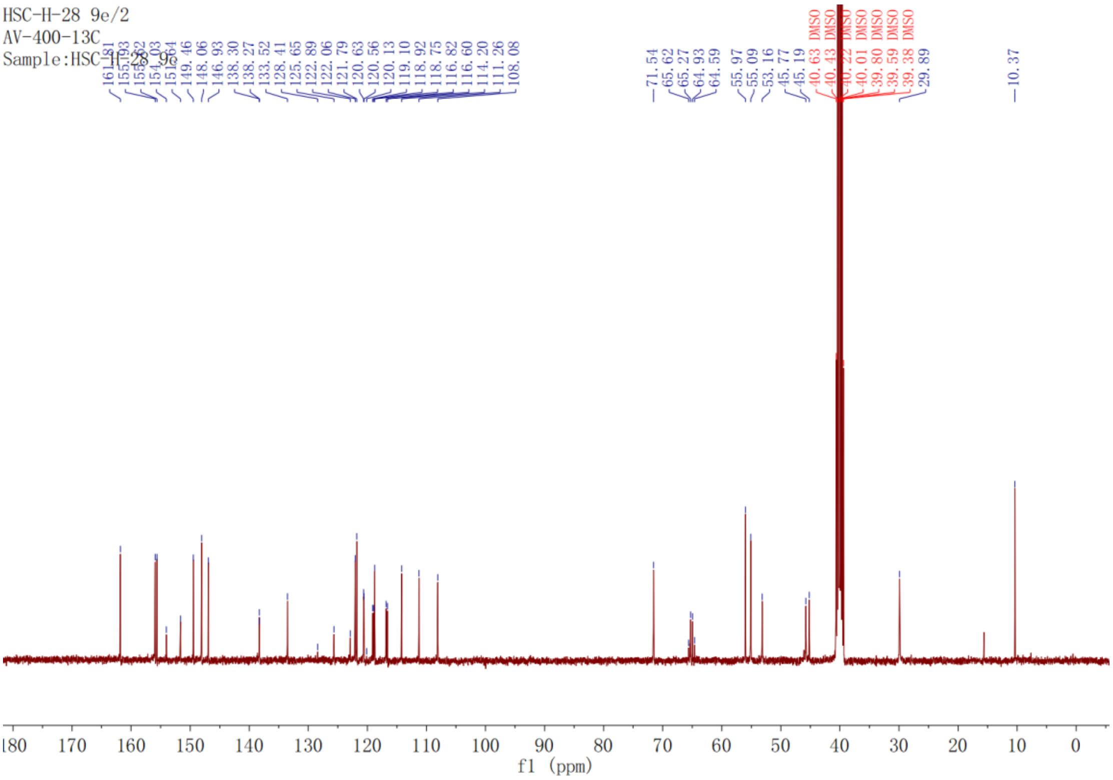

Figure S70: ^{13}C-NMR spectra of the target compound 9e

molecules MDPI

Mass Spectrum SmartFormula Report

Analysis Info

Analysis Name	D:\data\HSC-H-30 neg_000001.d	Acquisition Date	3/17/2021 4:06:15 PM
Method	100-1200_neg_20210312	Operator	
Sample Name		Instrument	solariX
Comment			

Acquisition Parameter

Acquisition Mode	Single MS	Acquired Scans	3	Calibration Date	Fri Mar 12 09:12:31 2021
Polarity	Negative	No. of Cell Fills	1	Data Acquisition Size	1048576
Broadband Low Mass	100.3 m/z	No. of Laser Shots	200	Data Processing Size (SI)	2097152
Broadband High Mass	1200.0 m/z	Laser Power	20.0 lp	Apodization	Full-Sine
Source Accumulation	0.000 sec	Laser Shot Frequency	0.001 sec		
Ion Accumulation Time	0.050 sec				

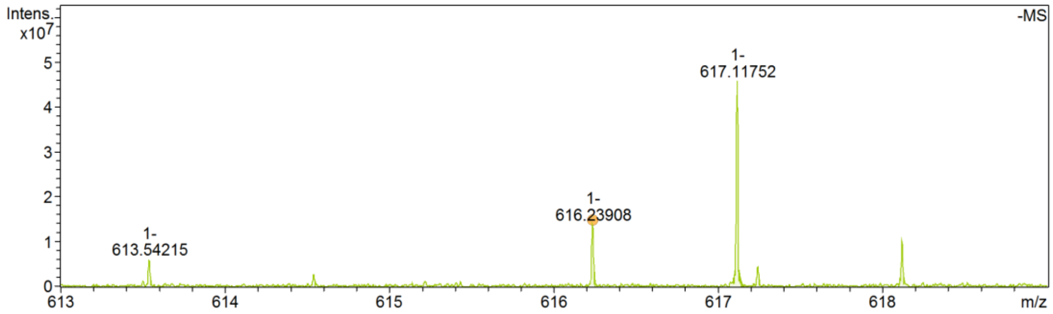

Meas. m/z	#	Ion Formula	Score	m/z	err [ppm]
616.239083	1	C30H33F3N5O6	100.00	616.238842	-0.4

Figuer S71: HRMS negative ion spectra of the target compound 9f

molecules MDPI

Mass Spectrum SmartFormula Report

Analysis Info

Analysis Name	D:\data\HSC-H-30 pos_000001.d	Acquisition Date	3/17/2021 4:03:39 PM
Method	100-1200_pos_20210312	Operator	
Sample Name		Instrument	solariX
Comment			

Acquisition Parameter

Acquisition Mode	Single MS	Acquired Scans	2	Calibration Date	Fri Mar 12 09:07:46 2021
Polarity	Positive	No. of Cell Fills	1	Data Acquisition Size	1048576
Broadband Low Mass	100.3 m/z	No. of Laser Shots	500	Data Processing Size (SI)	2097152
Broadband High Mass	1200.0 m/z	Laser Power	20.0 lp	Apodization	Full-Sine
Source Accumulation	0.000 sec	Laser Shot Frequency	0.001 sec		
Ion Accumulation Time	0.050 sec				

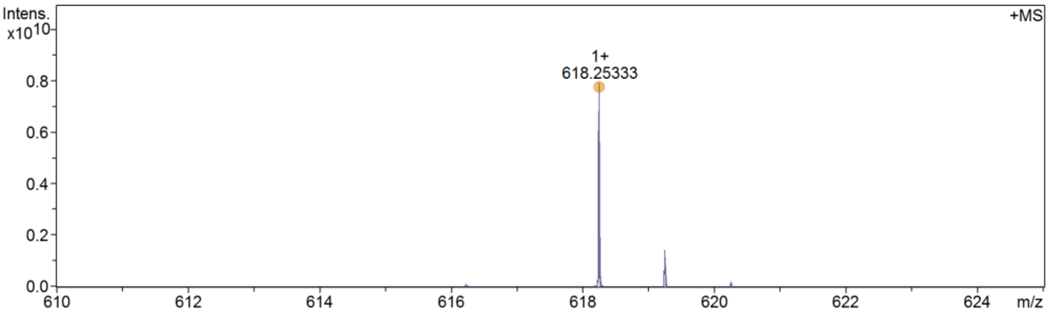

Meas. m/z	#	Ion Formula	Score	m/z	err [ppm]
618.253332	1	C30H35F3N5O6	100.00	618.253395	0.1

Figure S72: HRMS positive ion spectra of the target compound 9f

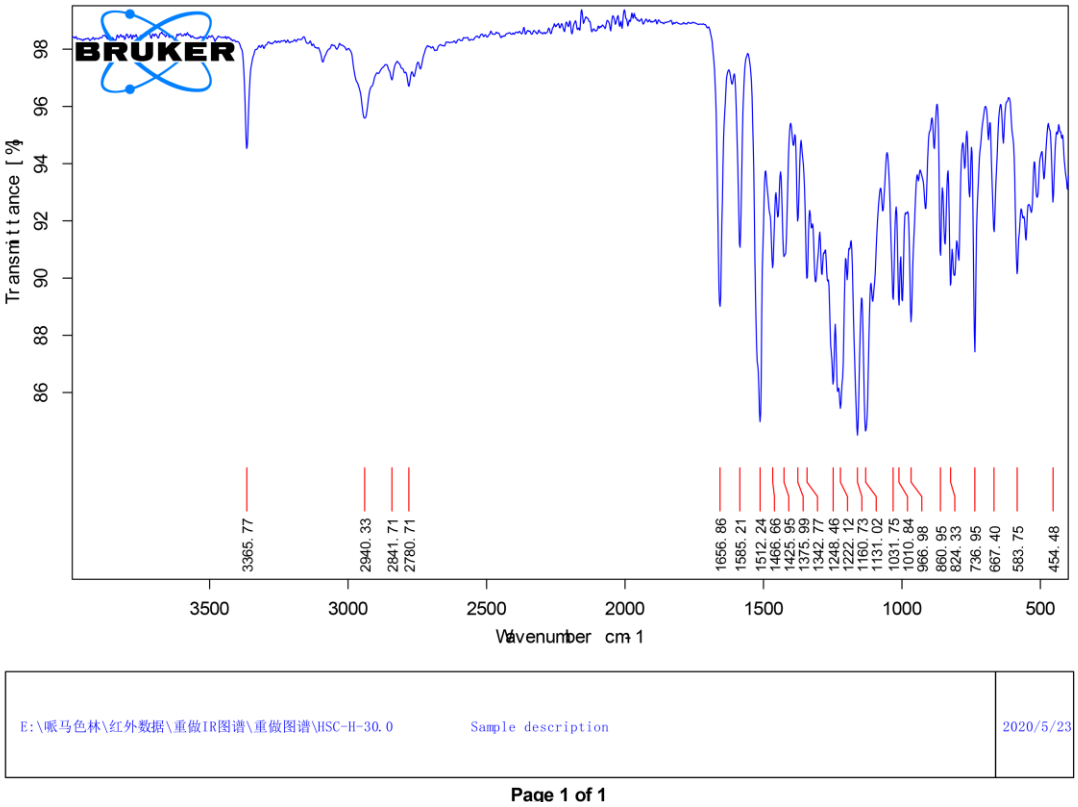

Figure S73: IR spectra of the target compound 9f

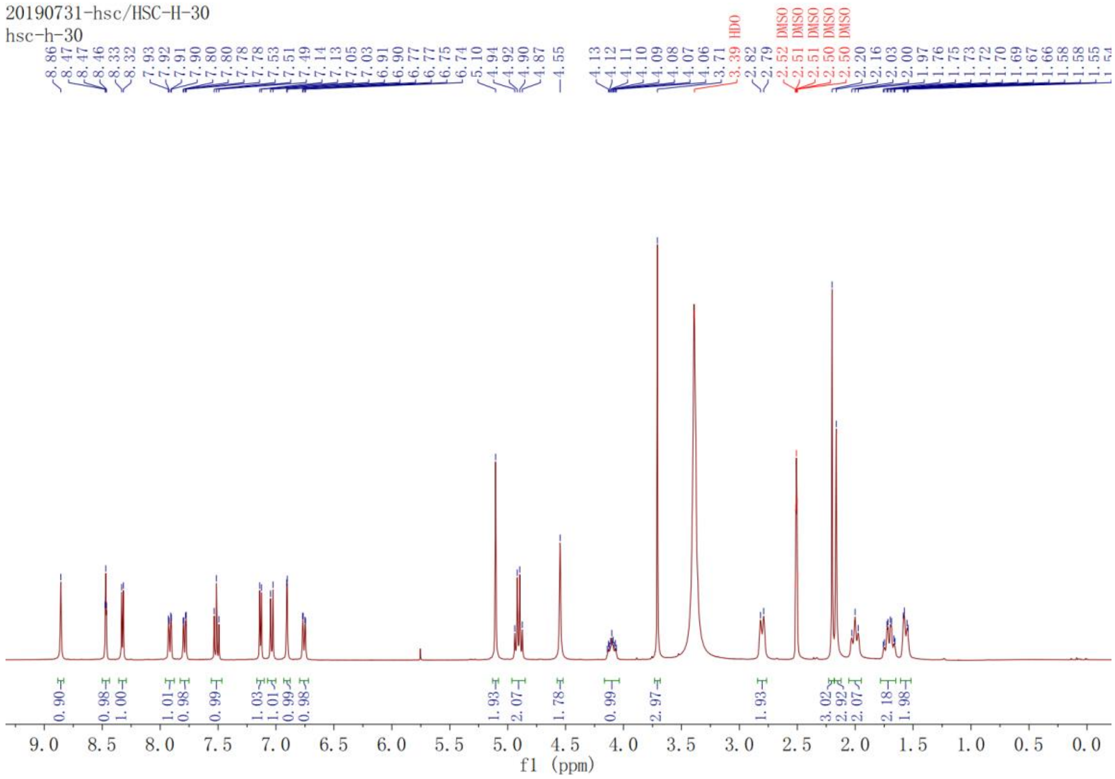

Figure S74: ^{1}H-NMR spectra of the target compound 9f

molecules

MDPI

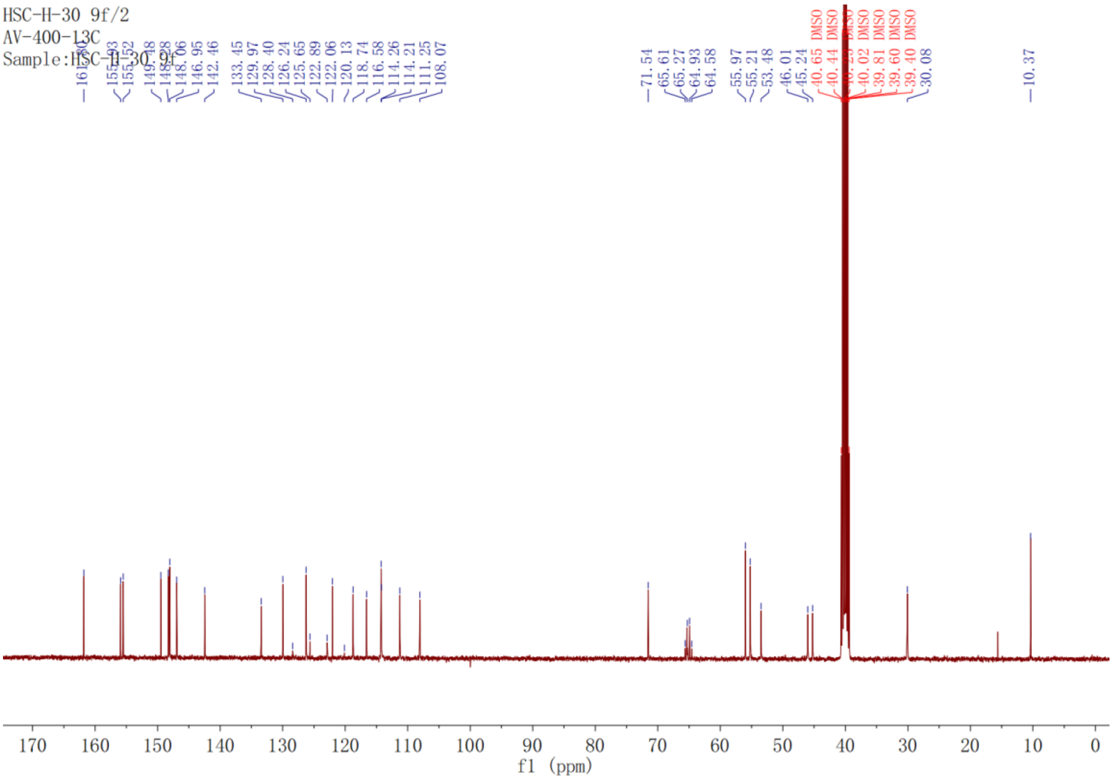

Figure S75: ^{13}C-NMR spectra of the target compound 9f

molecules MDPI

Mass Spectrum SmartFormula Report

Analysis Info

Analysis Name	D:\data\HSC-H-5 neg_000002.d	Acquisition Date	3/17/2021 6:14:17 PM
Method	100-1200_neg_20210312	Operator	
Sample Name		Instrument	solariX
Comment			

Acquisition Parameter

Acquisition Mode	Single MS	Acquired Scans	3	Calibration Date	Fri Mar 12 09:12:31 2021
Polarity	Negative	No. of Cell Fills	1	Data Acquisition Size	1048576
Broadband Low Mass	100.3 m/z	No. of Laser Shots	200	Data Processing Size (SI)	2097152
Broadband High Mass	1200.0 m/z	Laser Power	20.0 lp	Apodization	Full-Sine
Source Accumulation	0.000 sec	Laser Shot Frequency	0.001 sec		
Ion Accumulation Time	0.050 sec				

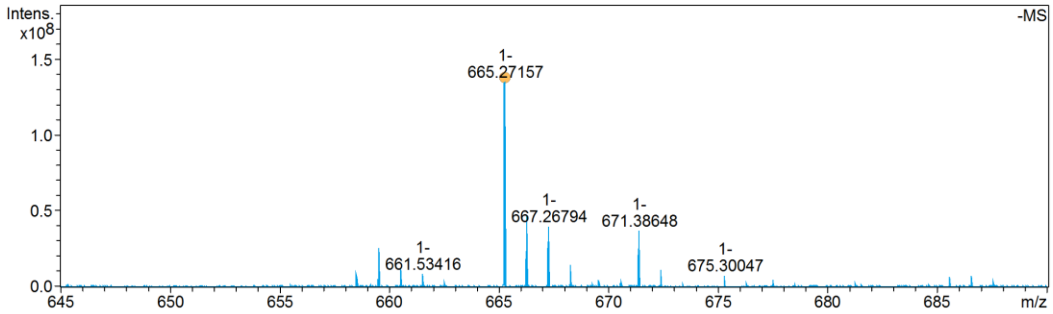

Meas. m/z	#	Ion Formula	Score	m/z	err [ppm]
665.271566	1	C33H41ClF3N4O5	100.00	665.272306	1.1

Figure S76: HRMS negative ion spectra of the target compound 9g

Mass Spectrum SmartFormula Report

Analysis Info

Analysis Name	D:\data\20210317\HSC-H-5 pos_000016.d	Acquisition Date	3/17/2021 6:37:18 PM
Method	100-1200_pos_20210312	Operator	
Sample Name		Instrument	solariX
Comment			

Acquisition Parameter

Acquisition Mode	Single MS	Acquired Scans	2	Calibration Date	Fri Mar 12 09:07:46 2021
Polarity	Positive	No. of Cell Fills	1	Data Acquisition Size	1048576
Broadband Low Mass	100.3 m/z	No. of Laser Shots	500	Data Processing Size (SI)	2097152
Broadband High Mass	1200.0 m/z	Laser Power	20.0 lp	Apodization	Full-Sine
Source Accumulation	0.000 sec	Laser Shot Frequency	0.001 sec		
Ion Accumulation Time	0.050 sec				

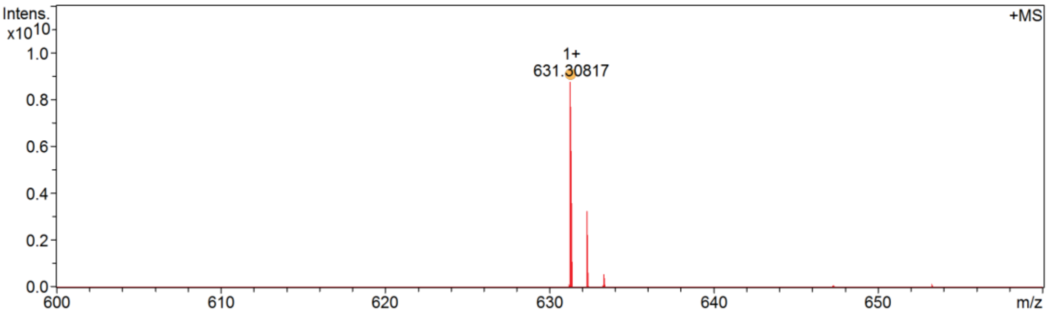

Meas. m/z	#	Ion Formula	Score	m/z	err [ppm]
631.308167	1	C33H42F3N4O5	100.00	631.310182	3.2

Figure S77: HRMS positive ion spectra of the target compound 9g

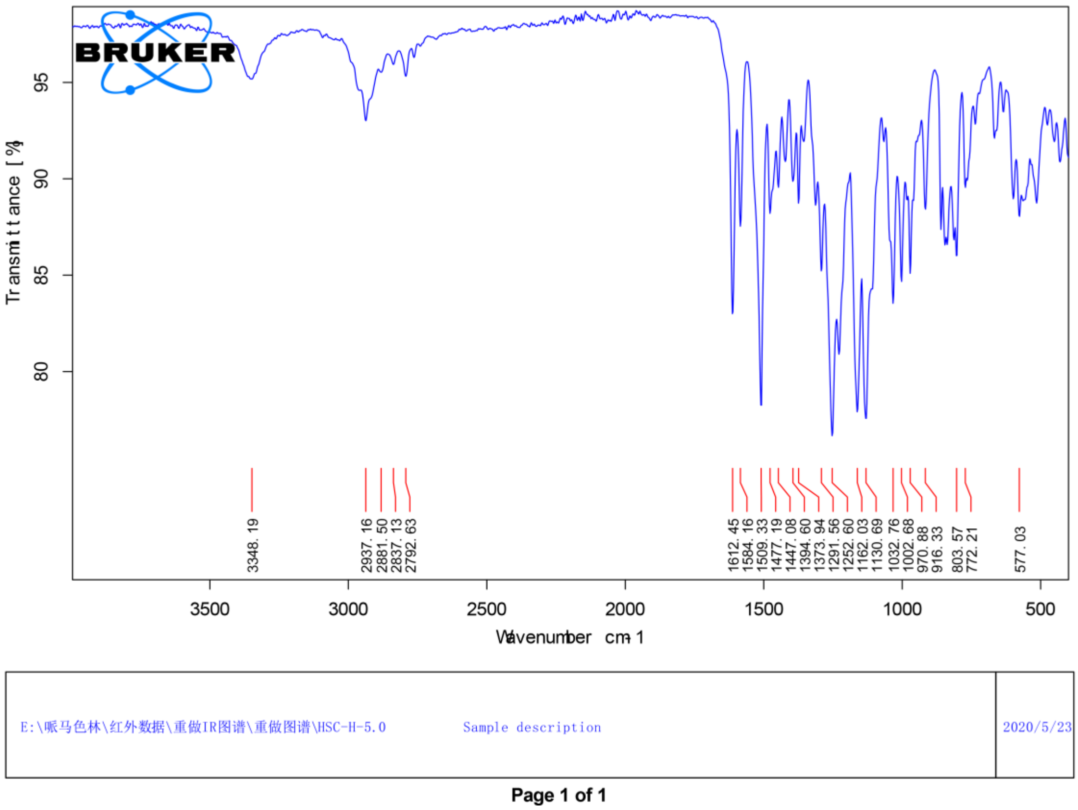

Figure S78: IR spectra of the target compound 9g

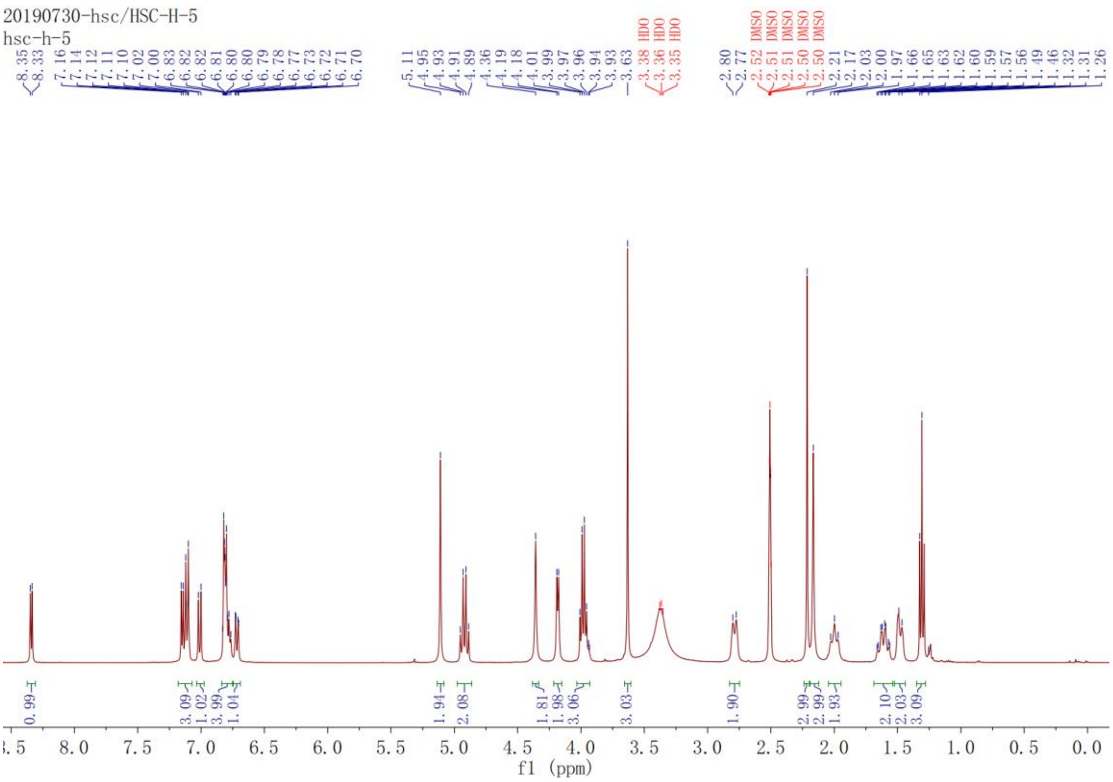

Figure S79: ^{1}H-NMR spectra of the target compound 9g

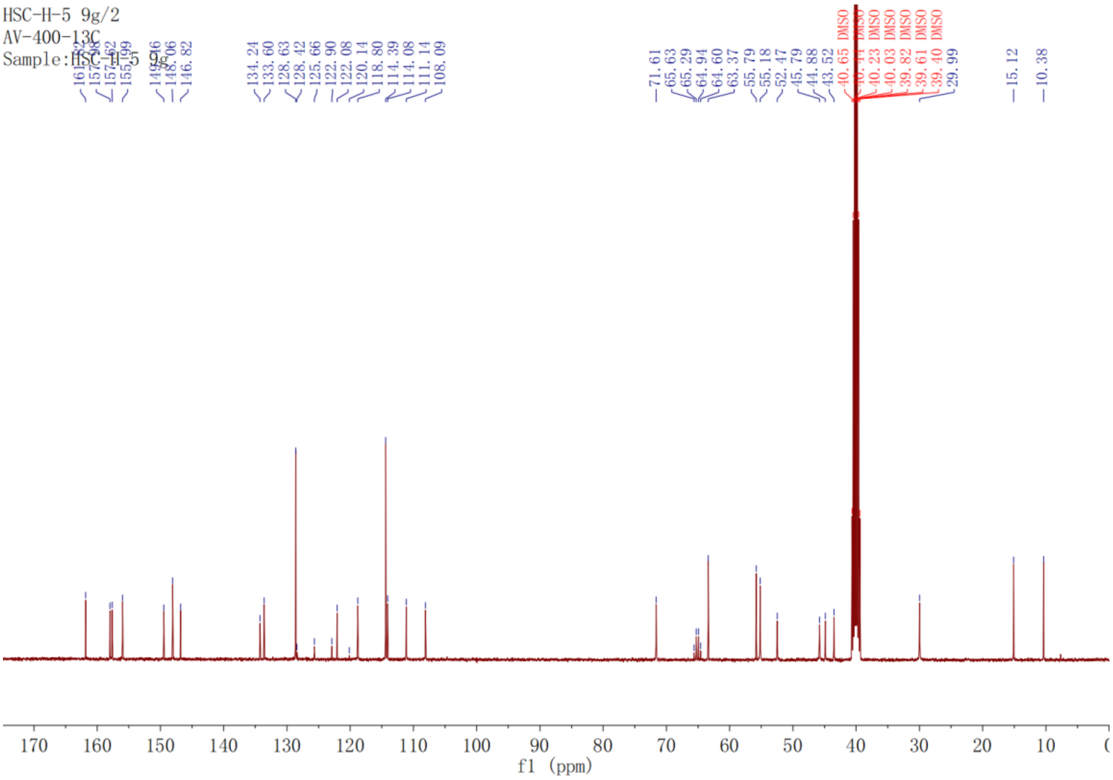

Figure S80: ^{13}C-NMR spectra of the target compound 9g
